# ACKR2 aids the regulation of bone marrow eosinophils to promote monocyte differentiation and anti-bacterial immunity

**DOI:** 10.1038/s41467-026-75964-z

**Published:** 2026-07-22

**Authors:** Gillian J. Wilson, Lily Koumbas Foley, Zuzanna Pocalun, Elise Pitmon, Ayumi Fukuoka, Kit M. Lee, Lauren Fernandez, Heather Mathie, Robin Bartolini, Laura Medina-Ruiz, John J. Cole, Gerard J. Graham

**Affiliations:** 1 Chemokine Research Group, School of Infection and Immunity, University of Glasgow, Glasgow, UK; 2 LifeArc, The Francis Crick Institute, London, UK; 3 Department of Immunology, University of Toronto, Toronto, ON Canada

**Keywords:** Chemokines, Infection

## Abstract

Chemokine receptors control cell migration within the body. Here we reveal interactions between eosinophils and monocytes in the bone marrow, indirectly controlled by the atypical chemokine receptor ACKR2. In the absence of ACKR2, there are decreased numbers of eosinophils in the bone marrow. As a result, eosinophil and monocyte interactions are reduced within the bone marrow niche, and are associated with changes in monocyte gene expression. Monocytes from ACKR2-/- mice are recruited to the tissues but are fundamentally altered in their ability to differentiate into macrophages, in the lung, peritoneal cavity and cavity wall. Bacterial elimination is impaired in ACKR2-/- mice during peritoneal infection. ACKR2 is therefore a key regulator of eosinophil-driven monocyte education in the bone marrow, required for full monocyte differentiation and macrophage function within the tissues.

## Abstract (web summary)

The atypical chemokine receptor ACKR2 aids regulation of eosinophil and monocyte interactions in the bone marrow. Here, the authors show that monocytes from ACKR2-/- mice are fundamentally altered in their ability to differentiate into macrophages and clear bacteria during infection.

## Introduction

Macrophages are key cells within the immune system, protecting the body by phagocytosing pathogens, helping to repair and remodel tissues after injury, and maintaining tissue homoeostasis^1^. Macrophages in the tissues can be broadly divided into resident or recruited cells. Resident macrophages are seeded embryonically and derive from yolk sac and foetal liver progenitors^2^. Recruited/inflammatory macrophages are derived from bone marrow monocytes which differentiate into macrophages following recruitment into tissues^3^.

Monocyte and macrophage movement within the body is largely orchestrated by members of the chemokine family^4,5^. Chemokines are a family of proteins characterised by a conserved cysteine motif and the large chemokine family is subdivided according to cysteine distribution into CC, CXC, XC and CX3C subfamilies. Chemokines act through G-protein-coupled receptors to direct cell migration^6,7^. Monocyte mobilisation and recruitment is predominantly regulated by the CC receptor 2 (CCR2), and CX3C receptor 1 (CX3CR1)^8^. In addition to signalling chemokine receptors, there is a subfamily of atypical chemokine receptors (ACKRs), that lack classical signalling responses to their ligands^7,9^. ACKR2 is a promiscuous scavenger receptor which binds, internalises and targets inflammatory CC-chemokines for degradation within the cell^6^. By modulating the availability of chemokines, ACKRs can regulate chemokine gradients and alter cell recruitment and activation. Together, signalling chemokine receptors and ACKRs coordinate leucocyte migration within the tissues^6^.

We, and others, have demonstrated an essential role for ACKR2 in resolving CC-chemokine driven inflammatory responses and in controlling the development of inflammation dependent cancers^10–15^. In addition, we have demonstrated that ACKR2 plays key roles in development, scavenging chemokines in the placenta, embryonic skin, and the pubertal mammary gland^16–19^. In embryonic skin, ACKR2 is expressed by lymphatic endothelial cells (LECs) and scavenges CCL2 to control the proximity of CCR2-expressing macrophages to developing vessels^18^. In the developing mammary gland, ACKR2 is expressed by fibroblasts and scavenges CCL7 to control CCR1-expressing macrophages and epithelial branching^16,17^.

Eosinophils are granulocytic immune cells derived from haematopoietic stem cells (HSC) in the bone marrow^20^. They are classically associated with the immune response in allergy or parasitic infection, but more recently, their roles in maintaining tissue homoeostasis and shaping development in the thymus have been identified^20,21^. Here we report a previously unknown role for eosinophils in educating monocytes within the bone marrow, indirectly controlled by the atypical chemokine receptor ACKR2. We show that reduced numbers of eosinophils within the bone marrow in ACKR2-/- mice is associated with reduced eosinophil/monocyte interactions within the bone marrow niche, and changes in monocyte gene expression. Monocytes from ACKR2-/- mice are recruited to the tissues but are fundamentally altered in their ability to differentiate into macrophages. Furthermore, in the absence of ACKR2, bacterial killing is impaired during peritoneal infection. Our data therefore demonstrate that ACKR2 is a key regulator of eosinophil-driven monocyte education in the bone marrow, which is required for full monocyte differentiation and ultimately macrophage function during infection.

## Results

### Macrophage numbers are decreased in the peritoneal cavity, cavity wall and lungs of ACKR2-/- mice

**Fig. 1:**
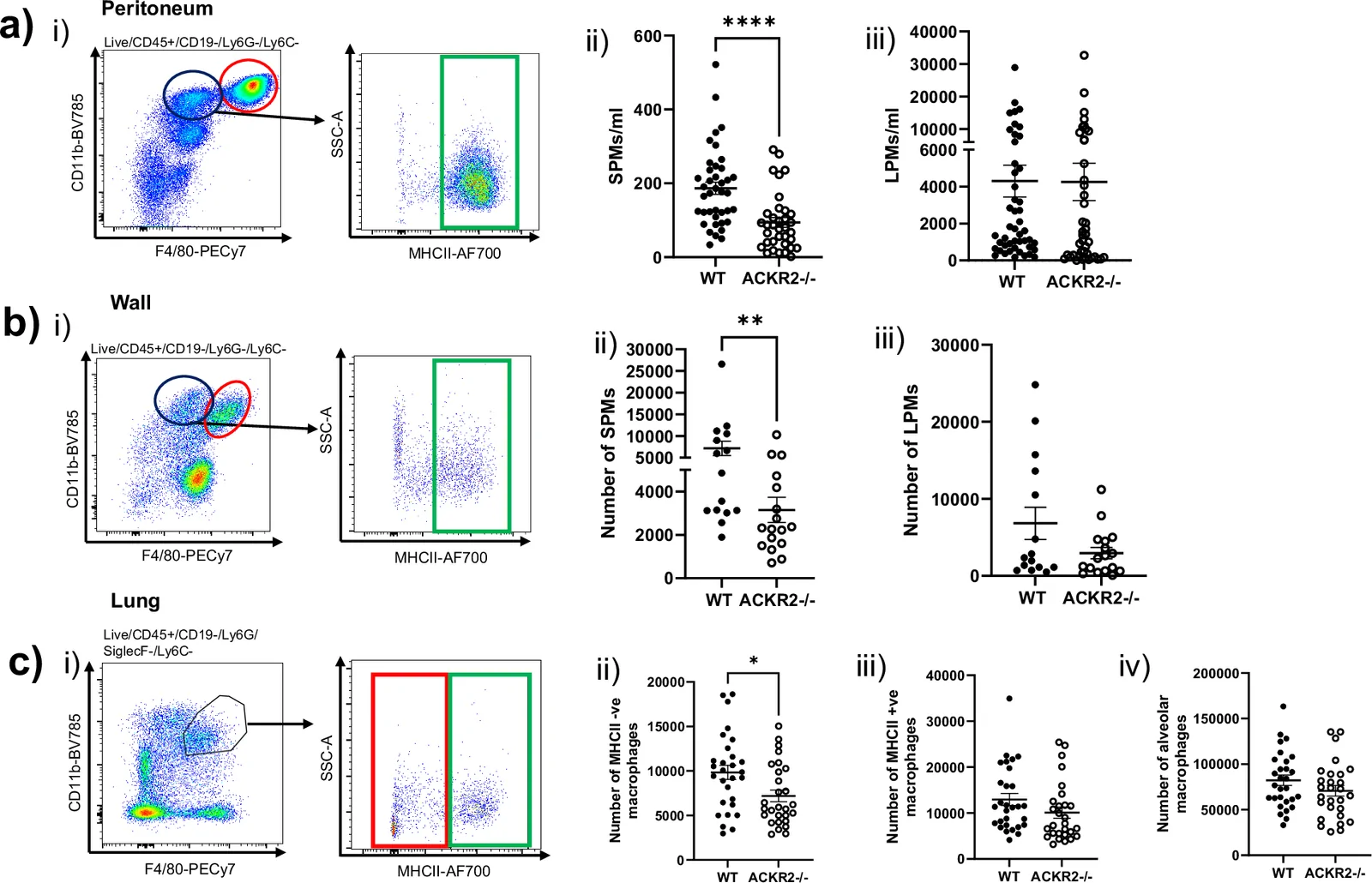
Small peritoneal macrophages and MHCII negative interstitial macrophages are decreased in ACKR2-/- mice. **ai** Gating strategy to define large peritoneal macrophages (LPMs, circled in red) and small peritoneal macrophages (SPMs, green box) in the peritoneal cavity. Flow cytometry was carried out to determine the number of **ii)** SPMs (WT *n* = 40, ACKR2-/- *n* = 35), two-tailed Mann-Whitney test, *p* < 0.0001, and **iii)** LPMs (WT *n* = 48, ACKR2-/- *n* = 42) in the peritoneal cavity. **bi** LPMs, circled in red, and SPMs, boxed in green, in the peritoneal cavity wall. The number of **ii** SPMs, two-tailed Mann-Whitney test, *p* = 0.0041 and **iii)** LPMs, WT *n* = 15, ACKR2-/- *n* = 17, in the peritoneal wall. **ci** Gating of lung interstitial macrophages, MHCII positive (green box) and MHCII negative (red box). The number of **ii** MHCII negative macrophages, WT *n* = 28, ACKR2-/- *n* = 27, two-tailed Mann-Whitney test, *p* = 0.0235. **iii** MHCII positive macrophages, WT *n* = 29, ACKR2-/- *n* = 28, and **iv** alveolar macrophages, WT *n* = 29, ACKR2-/- *n* = 28. Statistical analysis was carried out between WT and ACKR2-/- mice. Significantly different results are indicated (*, *p* ≤ 0.05, **, *p* ≤ 0.01, ***, *p* ≤ 0.001, ****, *p* ≤ 0.0001). Error bars represent SEM. Source data are provided as a Source Data file.

In the peritoneal cavity, there are two main macrophage populations, small peritoneal macrophages (SPMs) which differentiate from CCR2 expressing Ly6C high monocytes, and resident large peritoneal macrophages (LPMs) which are embryonically derived^22–25^. These populations were identified using the flow cytometry gating strategy shown in Supplementary Fig. 1. In the absence of ACKR2, the number of SPMs were reduced in the peritoneal fluid and the cavity wall of 8 week old female mice (Fig. 1a, b). We saw the same reduction in female mice of different ages, 4 and 12 weeks, and in male 8 week old mice (Supplementary Fig. 2). There was no effect on the number of resident LPMs in ACKR2-/- mice (Fig. 1a, b).

In the lung, there are resident alveolar macrophages, and diverse populations of recruited interstitial macrophages^26^. Here, we demonstrate that in the absence of ACKR2, there was again no difference in resident alveolar macrophages, but there were reduced numbers of the monocyte derived MHCII negative interstitial macrophage population (Fig. 1c, gated as shown in Supplementary Fig. 3). No significant differences were observed in resident or monocyte derived macrophages from the pleural cavity, spleen, omentum, skin, or bone marrow (Supplementary Fig. 4). Surface marker expression was distinct between the populations controlled by ACKR2 (Supplementary Fig. 5). SPMs are CD11b^high^, MerTK^+^, CD226^+^ and MHCII^+^, whereas the interstitial macrophages are CD11b^low^, MerTK^-^, CD226^-^, and MHCII^-^ suggesting the cell types are not similar, despite their shared dependence on ACKR2 (Supplementary Fig. 5). In the absence of ACKR2, the number of monocytes were unchanged in peritoneal fluid but modestly reduced in the peritoneal cavity wall (Supplementary Fig. 6). Monocyte numbers remained unchanged in all other tissues investigated (Supplementary Fig. 6). This reveals that ACKR2 deficiency does not affect monocyte migration to all tissues.

### Peritoneal immune response is impaired in ACKR2-/- mice during *Escherichia coli* infection

**Fig. 2:**
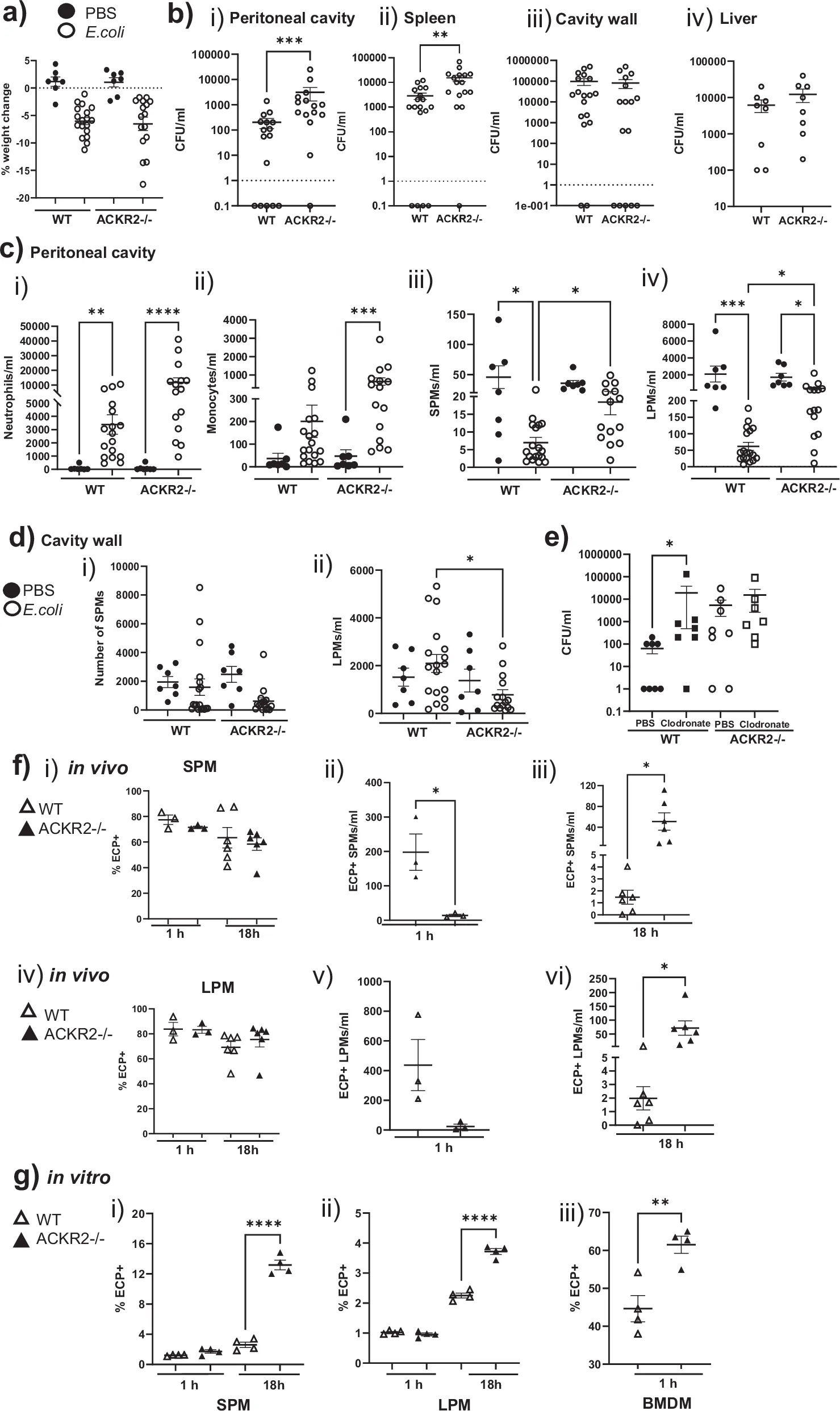
Bacterial elimination during *E.coli* infection is impaired in ACKR2-/- mice. **a** Weight change after i.p. challenge with either PBS (denoted by black circles) or 5 × 10^6^ CFU *E*. *coli* strain CFT073 (white circles), for 18 h (PBS, *n* = 7 per group, WT, *n* = 18, ACKR2-/-, *n* = 16). **b** Colony forming units (CFU) were counted 18 h after infection from **i** peritoneal fluid, WT, *n* = 16, ACKR2-/-, *n* = 15, two-tailed Mann-Whitney, *p* = 0.0009. **ii** spleen, WT, *n* = 17, ACKR2-/-, *n* = 16, two-tailed Mann-Whitney, *p* = 0.0018. **iii** cavity wall, WT, *n* = 18, ACKR2-/-, *n* = 16, and **iv** liver, *n* = 8 per group. **c** Flow cytometry was used to determine the number of cells in the peritoneal cavity 18 h after challenge with PBS (black circles) or 5 × 10^6^ CFU *E*. *coli* strain CFT073 (white circles). **i** neutrophils, PBS, *n* = 7 per group, WT, *n* = 17, ACKR2-/-, *n* = 15, Kruskal Wallis test with Dunn’s correction, WT PBS vs *E.coli*, *p* = 0.0086, ACKR2-/- PBS vs *E.coli*, *p* < 0.0001. **ii** monocytes, PBS, *n* = 7 per group, WT, *n* = 18, ACKR2-/-, *n* = 15, Kruskal Wallis test with Dunn’s correction, ACKR2-/- PBS vs *E.coli*, *p* = 0.0004. **iii** SPMs, PBS, *n* = 7 per group, WT, *n* = 18, ACKR2-/-, *n* = 14, Kruskal Wallis test with Dunn’s correction, WT PBS and *E.coli*, *p* = 0.0154, *E.coli* WT and ACKR2-/-, *p* = 0.048, and **iv)** LPMs, PBS, *n* = 7 per group, WT, *n* = 18, ACKR2-/-, *n* = 15, Kruskal Wallis test with Dunn’s correction, WT PBS and *E.coli* infected, *p* = 0.0001, WT and ACKR2-/- *E.coli*, *p* = 0.0221, ACKR2-/- PBS and *E.coli*, *p* = 0.0372. **d)** The number of cells in the peritoneal cavity wall 18 h after infection. **i** SPMs, PBS, *n* = 7 per group, WT, *n* = 18, ACKR2-/-, *n* = 16 and **ii** LPMs, PBS, *n* = 7 per group, WT, *n* = 18, ACKR2-/-, *n* = 15, Kruskal Wallis test with Dunn’s correction, WT and ACKR2-/- *E.coli*, *p* = 0.0192. **e)** CFU in peritoneal fluid after macrophages were depleted using clodronate liposomes, before an 18 h challenge with 5 × 10^6^ CFU *E. coli* CFT073 (PBS liposomes, denoted by circles, *n* = 8 per group, clodronate liposomes, denoted by squares, *n* = 7 per group, WT, black, ACKR2-/- white), Kruskal Wallis test with Dunn’s correction, WT control liposomes and clodronate, *p* = 0.0389. **f** Percentage of uptake by **i** SPMs and **iv** LPMs, after i.p. challenge with 200 µg *E.coli* particles, *n* = 3 per group. WT, denoted by white triangles, and ACKR2-/- denoted by black triangles. Number of ECP+ SPMs after **ii** 1 h, *n* = 3, unpaired, two-tailed *t* test, *p* = 0.0254, and **iii** 18 h, *n* = 6, unpaired, two-tailed *t* test, *p* = 0.0147 and number of ECP+ LPMs after **v** 1 h, *n* = 3, and **vi** 18 h, *n* = 6, unpaired, two-tailed *t* test, *p* = 0.0243. **g** Percentage of uptake by naïve **i** SPMs, *n* = 4 per group, Ordinary one way ANOVA with Bonferroni’s multiple comparison test, *p* < 0.0001 and **ii** LPMs, *n* = 4 per group, 1 h and 18 h after incubation with 1 µg/ml *E.coli* particles in vitro, Ordinary one way ANOVA with Bonferroni’s multiple comparison test, *p* < 0.0001, *n* = 4. **iii** Bone marrow derived macrophages (BMDM) 1 h after incubation with 1 µg/ml *E.coli* particles, in vitro, *n* = 4 per group, unpaired, two-tailed *t* test, *p* = 0.0065. Significantly different results are indicated (*, *p* ≤ 0.05, **, *p* ≤ 0.01, ***, *p* ≤ 0.001, ****, *p* ≤ 0.0001). Error bars represent SEM. Source data are provided as a Source Data file.

Next, we investigated whether reduced macrophage numbers as a result of ACKR2 deficiency had functional consequences during peritoneal infection with *E.coli*. Mice were injected intraperitoneally (i.p.) with 5 × 10^6^ colony forming units (CFU) of the uropathogenic (UPEC) isolate CFT2073. Weight loss was comparable between WT and ACKR2-/- animals 18 h after infection (Fig. 2a). However, the number of bacteria was increased in the peritoneal fluid and spleen of ACKR2-/- mice indicating that bacterial elimination was impaired (Fig. 2bi, ii). Bacterial burden was unchanged in the peritoneal cavity wall and liver during infection (Fig. 2biii, iv). During peritoneal infection there was increased recruitment of neutrophils to the peritoneal cavity of both WT and ACKR2-/- animals (Fig. 2ci), and monocytes in ACKR2-/- animals (Fig. 2cii).

The formation of mesothelia-bound multilayered cellular aggregates is an important component of bacterial elimination during peritoneal infection^27^. Here, we see the characteristic macrophage disappearance/ adherence reaction 18 h after infection in WT mice^24,27^, with a reduction in SPMS and LPMs in the peritoneal cavity (Fig. 2ciii, iv). However, in ACKR2-/- mice, increased numbers of SPMs and LPMs remain within the peritoneal cavity during infection, compared with WT, suggesting that the disappearance reaction is reduced (Fig. 2ciii, iv). When WT naïve and PBS injected animals are compared (Figs. 1aii, and 2ciii, respectively), there is also a reduction in the number of SPMs in the peritoneal cavity, after injection alone. We believe the injection itself may lead to a disappearance reaction in WT mice, which is reduced in ACKR2-/- animals. In the peritoneal wall there is no change in SPM number between WT and ACKR2-/- mice during infection (Fig. 2di). However, there are reduced numbers of LPMs in the wall of ACKR2-/- mice during infection, suggesting that the adherence reaction has been diminished, which may contribute to insufficient bacterial clearance (Fig. 2dii). However, it is notable that despite altered LPM numbers in the cavity wall of WT and ACKR2-/- mice, there is no difference in bacterial burden (Fig. 2aiii). This suggests that LPMs are not the key cell type responsible for bacterial elimination in the cavity wall.

To confirm the importance of macrophages in bacterial infection of the peritoneal cavity, macrophages were depleted using clodronate liposomes before bacterial challenge. The bacterial burden within the peritoneal cavity was increased in WT and unaffected in ACKR2-/- mice, indicating that macrophages are key to the observed phenotype (Fig. 2e). Notably, clodronate-mediated depletion of peritoneal macrophages was effective in WT, but not ACKR2-/- mice, suggesting the ability of macrophages from ACKR2-/- mice to phagocytose, release clodronate and cause cell death is impaired (Supplementary Fig. 7).

Next, we looked at the ability of macrophages to phagocytose pHRODO *E.coli* particles (ECP), which detect acidification after uptake. Initially, we challenged WT and ACKR2-/- mice i.p. with 200 µg of ECP. We found no difference in the percentage of peritoneal macrophages which phagocytosed particles 1 and 18 h post challenge, suggesting there was no defect in initial binding or uptake between SPMs or LPMs from WT and ACKR2-/- mice in vivo (Fig. 2fi, iv). However, cell migration and the macrophage disappearance/adherence reaction, which we have shown are altered in ACKR2-/- mice in vivo (Fig. 2c), are likely to affect this observation. When we investigated the total number of macrophages which had phagocytosed particles, we found fewer ECP+ SPMs after 1 h in ACKR2-/- mice (Fig. 2fii), likely due to decreased levels of SPMs in the peritoneal cavity, prior to infection (Fig. 1aii). Later, we detected increased numbers of ECP+ macrophages in ACKR2-/- mice, likely due to increased levels of SPMs and LPMs remaining in the peritoneal cavity of ACKR2-/- mice 18 h after challenge (Fig. 2ciii, vi).

To study particle uptake without the macrophage disappearance/adherence reaction, we also carried out ECP challenge of all cells within the peritoneal lavage in vitro. When macrophage numbers are constant in vitro, we observed no change in the percentage of ECP+ macrophages after 1 h, but an increased proportion of ECP+ macrophages after 18 h in ACKR2-/- mice (Fig. 2gi, ii).This suggests that while binding and initial uptake are unaffected, breakdown of pHRODO particles is reduced in the peritoneal macrophages from ACKR2-/- mice (Fig. 2g). When ECP particles are phagocytosed and destroyed by the macrophage, fluorescence is lost, therefore a higher percentage of ECP positive cells indicates deficient phagocytosis. We also generated bone marrow derived macrophages (BMDM) from WT and ACKR2-/- mice and challenged with ECP in vitro. There was an increased percentage of ECP+ macrophages in BMDMs from ACKR2-/- mice, revealing the destruction of particles is impaired, 1 h after challenge (Fig. 2giii). ECP particle breakdown by peritoneal macrophages appears to be more efficient in vitro than in vivo (Fig. 2f, g), which may be due to a number of factors such as different culture conditions, enhanced proximity of ECP particles to the cell in vitro, and cell turnover in vivo.

### ACKR2 has no detectable effect on chemokine levels in the peritoneal cavity

**Fig. 3:**
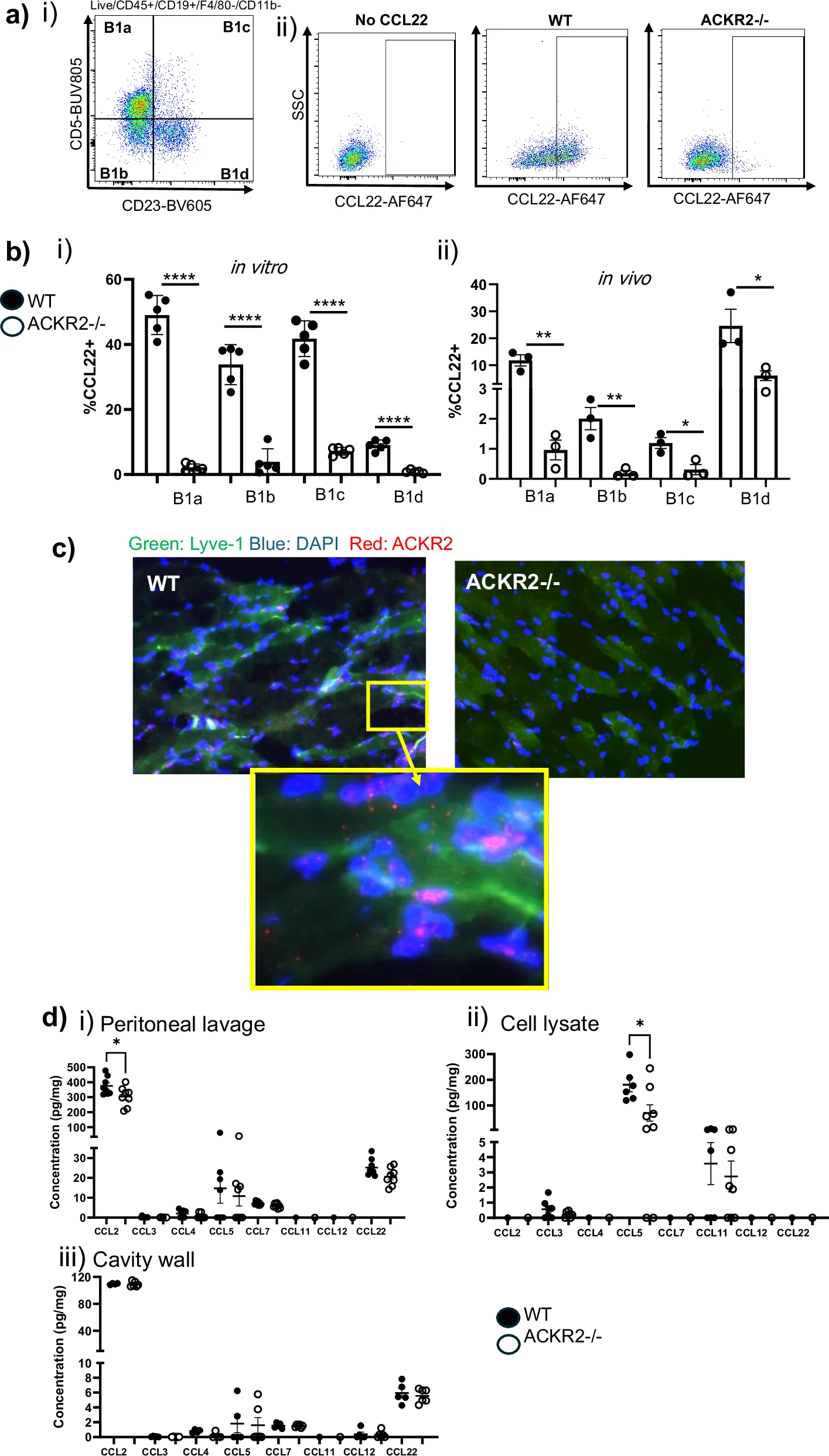
ACKR2 is expressed but has no detectable effect on chemokine scavenging in the peritoneal cavity. **a** Flow cytometry gating to define **i** B1 cell subsets within the peritoneal cavity based on CD5 and CD23 expression, and **ii** ACKR2 expression by CCL22 uptake. **b** Percentage of WT and ACKR2-/- B1 cells which are CCL22 positive after **i** incubation with 0.25 µg/ml AF647 labelled CCL22 in vitro, *n* = 5 per group, unpaired two-tailed *t*-tests, WT vs ACKR2-/-, a-d, *p* < 0.0001 and **ii** injection with 0.5 µg AF647 CCL22 in vivo, *n* = 3 per group, unpaired two-tailed *t*-tests, B1a, *p* = 0.00271, B1b *p* = 0.0089, B1c *p* = 0.0231 and B1d, *p* = 0.0465. **c** RNAscope in situ hybridisation of ACKR2 (red) and antibody staining of Lyve-1 (green) in the peritoneal cavity wall. Nuclear staining with DAPI (blue). **d** Luminex analysis was carried out to determine protein levels in **i** peritoneal lavage fluid (*n* = 8, per group), CCL2, unpaired two-tailed *t*-test, *p* = 0.0395 **ii** cell lysates (*n* = 8, per group), CCL5, two-tailed Mann-Whitney test *p* = 0.0413, and **iii** cavity wall lysates (WT *n* = 5, ACKR2-/- *n* = 6). Significantly different results are indicated (*, *p* ≤ 0.05, **, *p* ≤ 0.01, ***, *p* ≤ 0.001, ****, *p* ≤ 0.0001). Black circles denote WT and open circles, ACKR2-/-. Error bars represent SEM.

Next, we investigated the expression of ACKR2 by cells in the peritoneal cavity. ACKR2 expression has previously been demonstrated on peritoneal B1 cells^28^. As ACKR2 antibodies are known to be unreliable, we employed our well validated, functional chemokine uptake assay^17,29,30^. Uptake of AF647 labelled ligand, CCL22, by peritoneal lavage cells, was compared between WT and ACKR2-/- mice (Fig. 3a). We confirmed that ACKR2 is expressed by B1 cell subsets, B1a, b, c and d, in the peritoneal cavity using in vitro CCL22 uptake assays (Fig. 3b). The highest ACKR2 expression in vivo was identified on B1a and B1d cells, with between 10 and 20% CCL22 uptake by these populations, which account for around 30% and 10% of peritoneal cavity B cells, respectively. The number of B1 a, b, c and d cells were unchanged between WT and ACKR2-/- mice, however there was an increase in B1a and b cells between WT male and female mice, that was not observed in ACKR2-/- animals (Supplementary Fig. 8). ACKR2 expression in the peritoneal cavity wall was detected using RNAscope in situ hybridisation. ACKR2 expression was co-localised with Lyve-1+ stromal cells, indicating that ACKR2 is expressed by LECs in the cavity wall (Fig. 3c). Expression of ACKR2 by LECs in other tissues has been extensively characterised^18,31,32^.

Next, we looked at the effect of ACKR2 deficiency on ACKR2 ligands. We carried out Luminex protein analysis of supernatants from peritoneal lavage, and lysates from total peritoneal cells and the peritoneal cavity wall. Surprisingly, in the absence of ACKR2 we were unable to detect any defect in scavenging of any of the ACKR2 ligands investigated (Fig. 3d). When ACKR2 actively scavenges in vivo, the levels of ACKR2 ligands are altered between WT and ACKR2-/- mice. That we have not observed any increase in ACKR2 ligands in ACKR2-/- mice, suggests that ACKR2 expression is low and not functionally relevant in the peritoneal cavity. We found a decrease in CCL2 in the peritoneal lavage of ACKR2-/- mice, likely caused by decreased numbers of SPMs in the peritoneal cavity (Fig. 1aii). Similarly, 18 h after challenge with live *E.coli* or ECP, there are notable changes in ACKR2 ligands such as CCL3, CCL5, CCL12 and CCL22 but there was no defect observed in scavenging by ACKR2 (Supplementary Fig. 9), indicating that ACKR2 is not actively scavenging ligands during peritoneal cavity infection. These data imply that the SPM defect in ACKR2-/- mice relates to mechanisms operating outside the peritoneal cavity.

### Monocyte differentiation into macrophages is reduced in ACKR2-/- mice

**Fig. 4:**
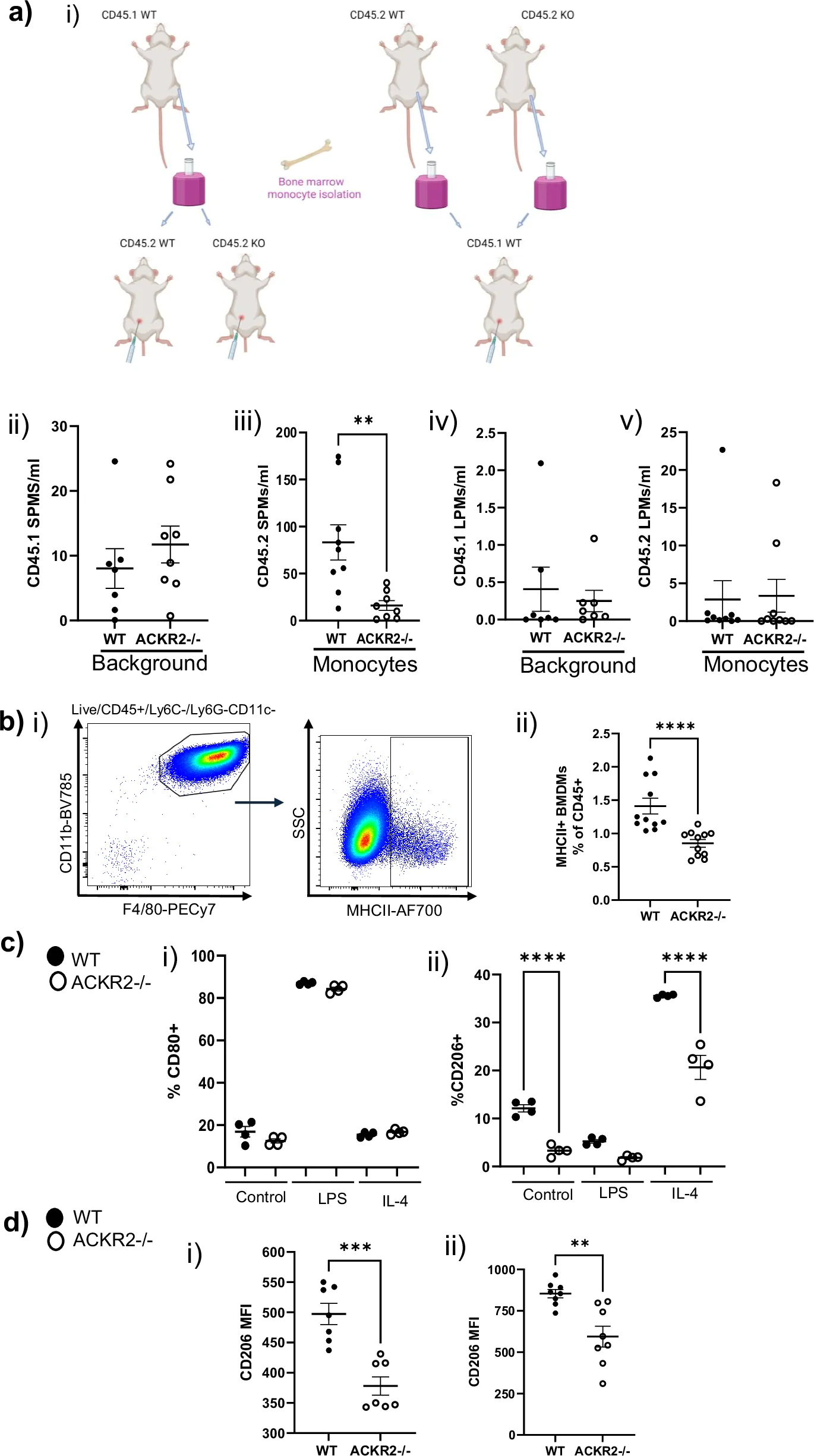
Monocyte differentiation into macrophages is reduced in the absence of ACKR2. **ai** Schematic of bone marrow monocyte adoptive transfer experiments. Created in BioRender. Wilson, G. (2026) https://BioRender.com/6b2reqv. **ii** Number of SPMs detected after 1 × 105 WT CD45.1 monocytes were injected i.p. into WT, *n* = 7 and ACKR2-/-, *n* = 8, CD45.2 mice. **iii** 1 × 10^5^ WT (*n* = 9) and ACKR2-/- (*n* = 8) CD45.2 monocytes were injected i.p. into WT CD45.1 mice, unpaired two-tailed *t*-test, *p* = 0.0052. Number of LPMs detected after **iv** CD45.1 *n* = 7, per group and **v** CD45.2 monocyte injection, *n* = 8. Cells were collected 2 days later by peritoneal lavage. **b** Bone marrow derived macrophages (BMDMs) were generated from whole bone marrow cultures. **i** BMDM gating is shown. **ii** Percentage of MHCII expressing BMDMs (*n* = 11 per group, where each point represents a culture dish, combined from at least 4 mice) on day 5 of culture, two-tailed Mann-Whitney, *p* < 0.0001. **ci** CD80 and **ii** CD206 expression on BMDM in response to LPS and IL-4, added overnight on day 5 of culture (*n* = 4 per group), ordinary one way ANOVA with Bonferroni’s multiple comparison test, *p* < 0.0001. **d** CD206 expression (MFI) on naïve **i** SPMs, *n* = 7 per group, two-tailed Mann-Whitney, *p* = 0.0006 and **ii** MHCII negative interstitial macrophages *n* = 8 per group, unpaired two-tailed *t*-test, *p* = 0.0019. Significantly different results are indicated (*, *p* ≤ 0.05, **, *p* ≤ 0.01, ***, *p* ≤ 0.001, ****, *p* ≤ 0.0001). Black circles denote WT and open circles, ACKR2-/-. Error bars represent SEM. Source data are provided as a Source Data file.

As we were unable to detect local effects of ACKR2 scavenging in the peritoneal cavity, or a reduction in monocyte numbers, we next investigated whether the reduced number of macrophages in the naïve ACKR2-/- peritoneal cavity was due to altered differentiation from monocytes. We investigated the ability of bone marrow monocytes from WT and ACKR2-/- mice to differentiate into macrophages in vivo, by adoptively transferring either CD45.1 or CD45.2 monocytes, and tracking differentiation (Fig. 4ai). To assess the ability of monocytes from WT mice to differentiate into SPMs in an ACKR2 deficient environment, we injected WT (CD45.1) monocytes i.p. into either WT (CD45.2) or ACKR2-/- (CD45.2) mice. Monocytes isolated from WT mice were fully able to differentiate in the ACKR2-/- peritoneal cavity (Fig. 4aii). Next, we looked at the ability of monocytes from WT (CD45.2) or ACKR2-/- (CD45.2) mice to differentiate into SPMs in a WT (CD45.1) environment. We found that monocytes from ACKR2-/- mice were less able to differentiate into SPMs, irrespective of being within a WT environment (Fig. 4aiii). LPMs are resident macrophages, there is little differentiation from monocytes to LPMs, and no difference between WT and ACKR2-/- mice (Fig. 4aiv, v). These data point to a monocyte/macrophage-autonomous dysfunction, rather than a peritoneal dysfunction, underlying the observed phenotype.

We next studied the ability of monocytes from WT and ACKR2-/- bone marrow to differentiate in vitro. In unstimulated BMDMs generated from ACKR2-/- mice, we observed a decreased proportion which express MHCII compared with WT, after 6D in culture (Fig. 4bii). To investigate whether BMDMs generated from WT and ACKR2-/- mice are altered in their ability to become either pro or anti-inflammatory macrophages, they were stimulated with either LPS or IL-4 overnight on day 5 of culture, and expression of the classical activation marker, CD80 or the alternative activation marker, CD206, was assessed. Upon stimulation, BMDMs from WT and ACKR2-/- mice had equivalent levels of CD80 expression in response to LPS (Fig. 4ci). However, we observed diminished CD206 expression by macrophages generated from ACKR2-/- bone marrow, at rest or in response to IL-4 (Fig. 4cii). Notably, we also observed lower CD206 expression in the key populations in naïve adult ACKR2-/- mice, i.e. SPMs and MHCII negative interstitial macrophages (Fig. 4d).

### Monocytes and macrophages are transcriptionally distinct in the absence of ACKR2

**Fig. 5:**
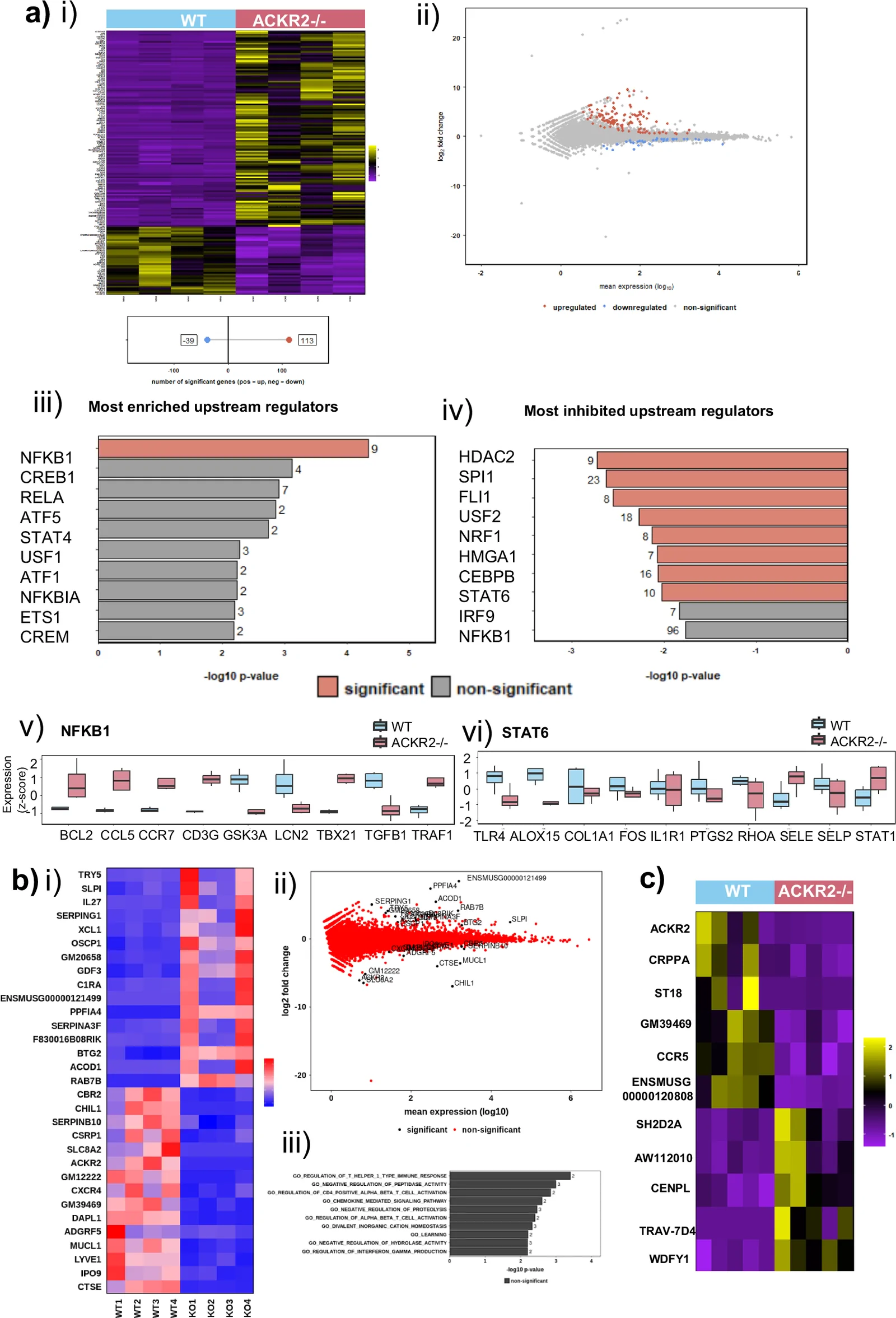
Monocytes and macrophages are transcriptionally distinct between WT and ACKR2-/- mice. **a** Bulk RNA sequencing was carried out of FACS sorted monocytes from the bone marrow of resting WT and ACKR2-/- mice (*n* = 4 per group). **i** Hierarchically clustered heatmaps of the significantly differentially expressed genes between samples. **ii** MA plot to show the expression of each gene and fold change between samples. Pathway analysis of **iii** enriched upstream regulators and **iv** inhibited upstream regulators. Summary boxplots of the **v** enriched upstream regulator NFKB1 and **vi** inhibited upstream regulator STAT6. Blue boxes represent WT and pink boxes, ACKR2-/-. Enrichment was calculated using Hypergeometric test with Benjamini–Hochberg (BH) correction on all significantly differentially expressed genes (*p*.adj <0.05, absolute log2 fold > 0.5). Upstream regulator analysis was performed on the databases TRRUST. Boxplots show the median (centre line), 25th and 75th percentiles (lower and upper hinges), and minimum and maximum values. The upper whisker extends from the hinge to the largest value no further than 1.5 * IQR from the hinge (where IQR is the inter-quartile range, or distance between the first and third quartiles). **b** RNA sequencing of FACS sorted SPMs from the peritoneal cavity of naïve WT and ACKR2-/- mice (*n* = 4 per group). **i** Hierarchically clustered heatmaps, **ii** MA plot and **iii**) pathway analysis. **c** RNA sequencing of FACS sorted MHCII negative interstitial macrophages from the lungs of naïve WT and ACKR2-/- mice (*n* = 5 per group). Bioinformatic and statistical analysis was carried out using Searchlight 2 (v2.0.3), (*p*.adj < 0.05, absolute log2 fold > 0.5)^63^. Sequencing data is available on Genbank, GEO accession number GSE334547.

RNA sequencing of FACS sorted monocytes from the bone marrow, gated as described in Supplementary Fig. 10, was carried out to investigate whether ACKR2 deficiency is associated with fundamental transcriptional differences in monocyte development. Transcriptional analysis revealed substantial changes in monocytes from ACKR2-/- mice, with 113 genes upregulated and 39 downregulated (Fig. 5a). Notably, ACKR2 transcription was not detected on WT monocytes. Pathway analysis revealed a number of changes associated with the NFKB1 pathway, including reduced TGFB1, which is known to play an important role in alternative macrophage activation (Fig. 5aiii, v)^33,34^. There are also key changes in genes, such as ALOX15, associated with the upstream regulator, signal transducer and activator of transcription 6 (STAT6) (Fig. 5aiv, vi), which is critical for IL-4 signalling^35^. We also carried out RNA sequencing of FACS sorted SPMs from the peritoneal cavity of WT and ACKR2-/- mice. In mature macrophages, ACKR2 deficiency led to distinct transcriptional changes. 16 upregulated and 15 downregulated genes were identified in SPMs from ACKR2-/- mice (Fig. 5bi). Pathway analysis revealed no significantly altered pathways (Fig. 5biii).

We also carried out RNA sequencing of FACS sorted MHCII negative macrophages from the lungs of WT and ACKR2-/- mice. Again, we observed a small number of specific transcriptional changes in mature macrophages, including 6 upregulated and 5 downregulated genes (Fig. 5c). Except for ACKR2, there were no differentially expressed genes in common between MHCII negative lung macrophages and SPMs from WT and ACKR2-/- mice (Fig. 5). This suggests that ACKR2 deficiency is associated with limited but specific differential gene regulation in macrophages in different tissues.

ACKR2 expression on SPMs and MHCII negative interstitial macrophages has not previously been detected using the functional CCL22 uptake assay^30^, however RNA sequencing revealed low levels of transcription (Fig. 5b, c). Notably, CXCR4, a key receptor involved in monocyte distribution^36^, is decreased in ACKR2-/- SPMs (Fig. 5b). However, there was no significant difference in CXCR4 levels in monocytes or MHCII negative interstitial macrophages, suggesting this is not central to the observed phenotype (Fig. 5a, c). Transcription of the dominant monocyte chemokine receptor, CCR2, was unchanged in bone marrow monocytes, SPMs or MHCII negative lung interstitial macrophages from WT and ACKR2-/- mice (Fig. 5b, c).

### ACKR2 regulates eosinophils in the bone marrow and blood

**Fig. 6:**
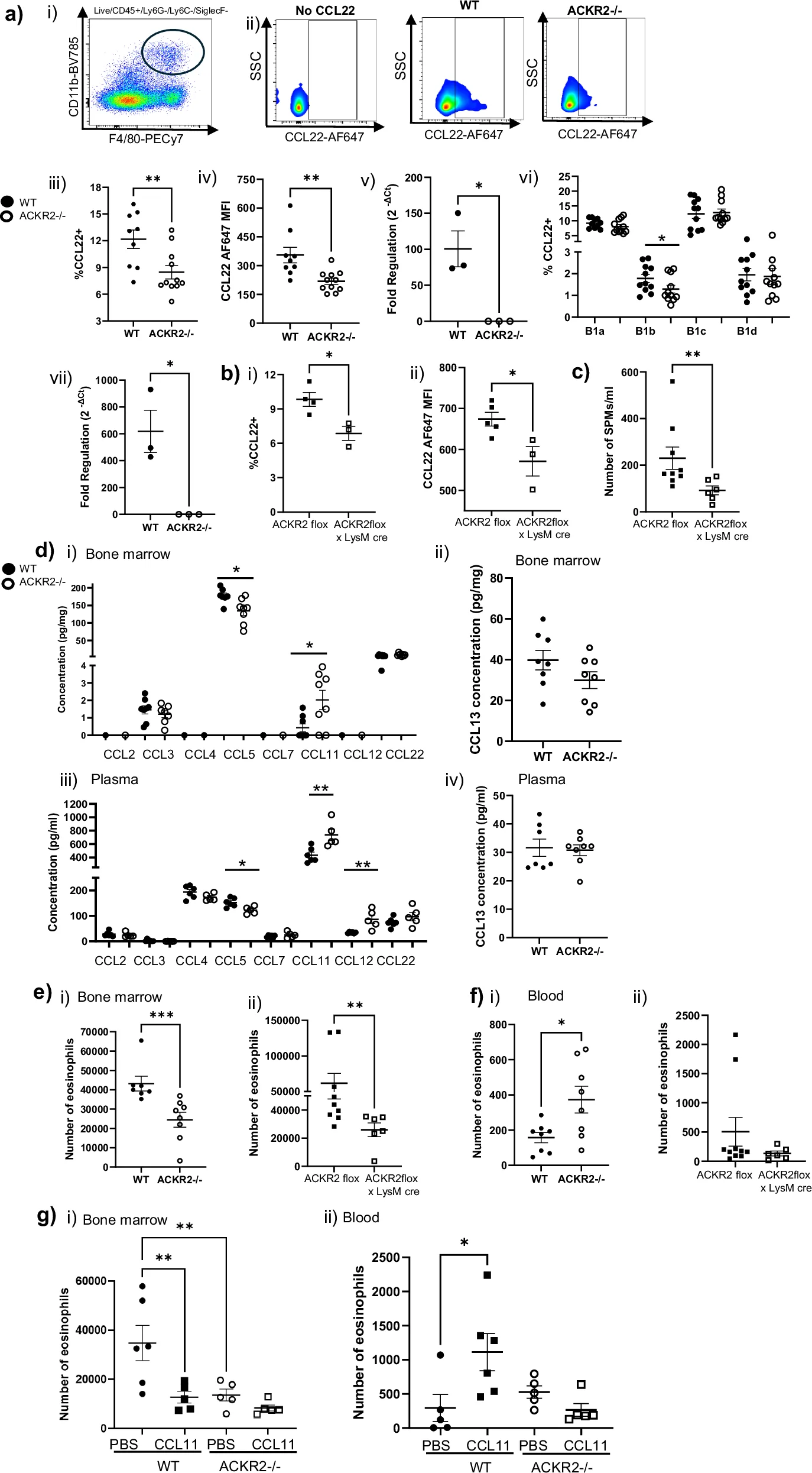
ACKR2 contributes to the regulation of eosinophil levels in the bone marrow and blood. **a** Flow cytometry gating strategy to define **i** CD11b + F4/80+ macrophages within the bone marrow (BM) and **ii** percentage CCL22 uptake. In BM macrophages the **iii** percentage of AF647 positive cells, two-tailed Mann-Whitney, *p* = 0.0097 and **iv** CCL22-AF647 MFIs were quantified, unpaired two-tailed *t*-test, *p* = 0.0034 (WT, *n* = 9, ACKR2-/-, *n* = 11). **v** qRT-PCR analysis of ACKR2 transcription by FACS sorted macrophages, *n* = 3 per group, unpaired two-tailed *t*-test, *p* = 0.0158. Percentage of **vi** AF647 positive B1 cells (*n* = 11), B1b, two-tailed Mann-Whitney, *p* = 0.0418. ACKR2 transcription by FACS sorted **vii** B1b cells, *n* = 3, unpaired, two-tailed *t* test, *p* = 0.0171. **b** ACKR2 expression by BM macrophages **i** percentage CCL22 positivity, unpaired two-tailed *t*-test, *p* = 0.0195, (ACKR2 flox, *n* = 4, ACKR2 flox x LysM cre, *n* = 3) and **ii** CCL22-AF647 MFI, unpaired two-tailed *t*-test, *p* = 0.0243, (ACKR2 flox, *n* = 5, ACKR2 flox x LysM cre, *n* = 3). **c** Flow cytometry was used to determine the number of SPMs in the peritoneal cavity (ACKR2 flox, *n* = 9, ACKR2 flox x LysM cre, *n* = 6), two-tailed Mann-Whitney, *p* = 0.0048. **d** Protein concentration analysis was carried out to determine protein levels in **i** Luminex of BM cell lysates (*n* = 8 per group), WT vs ACKR2-/-, CCL5, unpaired two-tailed *t*-test *p* = 0.0139, CCL11, two-tailed Mann-Whitney *p* = 0.046. **ii** ELISA of BM cell lysates (WT, *n* = 7, ACKR2-/-, *n* = 8), **iii** Luminex of plasma, WT vs ACKR2-/-, CCL5, unpaired two-tailed *t*-test, *p* = 0.0123, CCL11, *p* = 0.009 and CCL12, *p* = 0.0064 (WT, *n* = 5, ACKR2-/-, *n* = 6), and **iv** ELISA of plasma (*n* = 8). **e** Flow cytometry was used to determine the number of eosinophils in the bone marrow of **i** WT (*n* = 7) and ACKR2-/- (*n* = 8) mice, two-tailed Mann-Whitney, *p* = 0.0006, and **ii** ACKR2 flox, (*n* = 9), and ACKR2 flox x LysM cre (*n* = 6) mice, Mann-Whitney, *p* = 0.0076. **f** The number of eosinophils in the blood of **i** WT and ACKR2-/- mice (*n* = 8, per group), unpaired two-tailed *t*-test, *p* = 0.0193 and **ii** ACKR2 flox, (*n* = 10), and ACKR2 flox x LysM cre (*n* = 6) mice. **g** PBS or 1 µg of CCL11 was injected intravenously into WT and ACKR2-/- mice and the number of eosinophils were measured in the **i** bone marrow (*n* = 5 per group except WT PBS, *n* = 6), ordinary one way ANOVA with Bonferroni correction, WT PBS vs WT CCL11 *p* = 0.0073, WT PBS vs ACKR2-/- PBS, *p* = 0.01, and **ii** blood (*n* = 5, except WT CCL11), ordinary one way ANOVA with Bonferroni correction, WT PBS vs WT CCL11, *p* = 0.0043. Significantly different results are indicated (*, *p* ≤ 0.05, **, *p* ≤ 0.01, ***, *p* ≤ 0.001, ****, *p* ≤ 0.0001). Error bars represent SEM. Source data are provided as a Source Data file.

Next, we investigated expression of ACKR2 in the bone marrow, using the AF647-CCL22 uptake assay, gated, as shown in Fig. 6ai, ii. CCL22 uptake was increased in WT, compared with ACKR2-/- CD11b + F4/80+ macrophages, and B1b cells, suggesting that ACKR2 is expressed by these cell types in the bone marrow (Fig. 6aiii, iv, vi). We confirmed ACKR2 was transcribed by macrophages and B1b cells, by performing qRT-PCR analysis on FACS-sorted bone marrow cells (Fig. 6av, vii). A previous study showed transcription of ACKR2 by HSC progenitors in the bone marrow^14^. However, our uptake assay does not show ACKR2 expression by LSK (Lin- Sca1+ c-Kit+) cells or common myeloid progenitors (CMP) (Supplementary Fig. 11).

To test the role of ACKR2 specifically on macrophages, we generated an ACKR2 flox mouse crossed with LysM Cre to delete ACKR2 specifically on myeloid cells. Again, CCL22 uptake is reduced in bone marrow macrophages obtained from these mice, compared with ACKR2 flox control mice (Fig. 6bi, ii). Importantly, SPMs are also reduced in ACKR2 flox/LysM cre mice (Fig. 6c), revealing that ACKR2 expression on macrophages and not B1b cells in the bone marrow is required for this phenotype.

Luminex analysis of bone marrow cell lysates and plasma from WT and ACKR2-/- mice, revealed increased levels of the ACKR2 ligand, and eosinophil chemoattractant, CCL11, in ACKR2-/- animals (Fig. 6di, iii). However, the level of chemokine detected is low, and as the bone marrow data was generated from cell lysates, we are unable to conclude whether CCL11 levels are intracellular or extracellular, and thus whether alterations are specifically a consequence of altered scavenging. The ACKR2 ligand, CCL12 is increased in the plasma of ACKR2-/- mice, suggesting there is scavenging by vascular ACKR2 in WT mice (Fig. 6diii). However, there is no change in CCL12 levels within the bone marrow of ACKR2-/- mice (Fig. 6di), or in the number of monocytes which express the receptor for CCL12, CCR2, in the blood or bone marrow (Supplementary Fig. 6h, i). CCL5 is also an ACKR2 ligand, which is altered in the bone marrow (Fig. 6di). ACKR2 is a scavenging receptor, therefore in ACKR2 deficient tissues elevated protein levels of chemokine ligands may indicate defective scavenging. The decreased levels of CCL5 in ACKR2-/- mice observed here are therefore not due to deficient scavenging by the receptor. RNA sequencing revealed decreased levels of CCL5 transcription by monocytes in ACKR2-/- mice, which may account for reduced levels of CCL5 protein in ACKR2-/- blood and bone marrow (Fig. 5). As CCL5 is produced by T cells and megakaryocytes in the bone marrow and by platelets in the blood we compared the number of these cell types, and found no significant differences between WT and ACKR2-/- animals (Supplementary Fig. 12).

Flow cytometry revealed that in the absence of ACKR2, the number of eosinophils was reduced in the bone marrow and increased in the blood (Fig. 6ei, fi). When ACKR2 is deleted specifically on macrophages, eosinophils are similarly reduced in the bone marrow, revealing that macrophage-expressed ACKR2 is sufficient to maintain eosinophil levels in the bone marrow (Fig. 6eii). As expected, there is no increase in blood eosinophils in ACKR2 flox x LysM cre mice, as ACKR2 expression on stromal cells such as LECs is intact (Fig. 6fii). It has been established previously that CCL11 mobilises eosinophils within the bone marrow to enter the blood^37^. We also demonstrated that increased CCL11 in the bloodstream leads to eosinophil mobilisation from the bone marrow, by injecting 1 µg CCL11 intravenously (i.v.) into WT mice, and measuring levels of eosinophils in the blood and bone marrow after 1 day (Fig. 6g). There was no change observed in any other cell types investigated (Supplementary Fig. 13). CCL11 injection into ACKR2-/- mice does not further increase eosinophil mobilisation from the bone marrow (Fig. 6gi). Eosinophils are not increased in the bloodstream of ACKR2-/- mice after CCL11 injection (Fig. 6gii). It is important to note that a high dose of CCL11 was used, due to the known short half-life of chemokines in vivo^38^, this may not inform the impact of small changes in CCL11 in the bone marrow.

ACKR2 shares 5 ligands with the eosinophil receptor CCR3; CCLs 4, 5, 7, 11 and 13. We carried out Luminex analysis of CCLs 4, 5, 7, and 11, and ELISA of CCL13. Only CCL11 showed increased levels in bone marrow, consistent with deficient scavenging in ACKR2-/- mice, however the amount of chemokine detected was low and we were unable to conclude whether this increase was due to scavenging (Fig. 6di, ii). We obtained bone marrow from WT and CCR3-/- mice and found that in the absence of the CCR3 receptor, eosinophils accumulate in the bone marrow. In particular, a population of mature SiglecF high eosinophils were identified in CCR3-/- mice, which are absent from WT bone marrow (Supplementary Fig. 14).

### Eosinophils promote monocyte to macrophage differentiation

**Fig. 7:**
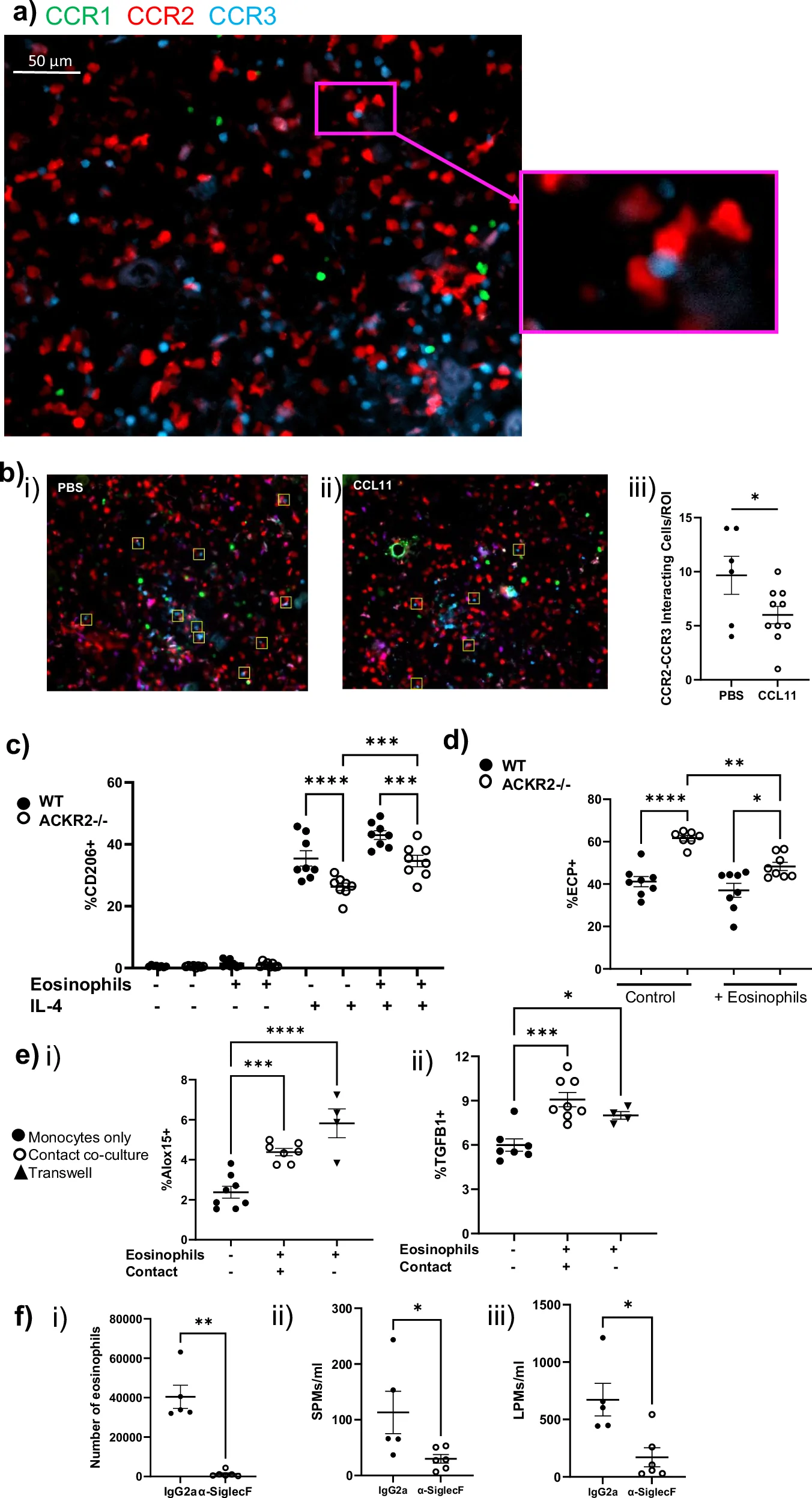
Eosinophils promote monocyte to macrophage differentiation. **a** Representative image of bone marrow from iREP mice imaged at 20 x magnification (*n* = 4). CCR1 expression is green, CCR2 is red, and CCR3 is blue. **b** Representative 20 x images of **i** PBS and **ii** 1 µg CCL11 injected bone marrow after 5 h, PBS, *n* = 3 and CCL11, *n* = 4. CCR2 + /CCR3+ interactions are boxed in yellow, and **iii** CCR2 + /CCR3+ interactions were quantified in defined regions of interest (ROI), PBS, *n* = 6 and CCL11, *n* = 10, two-tailed, unpaired t-test, *p* = 0.0486. **c** WT (black circles) and ACKR2-/- (white circles) BMDMs were generated with and without FACS sorted WT eosinophils (90% purity) on day 0, and with and without IL-4 treatment for 18 h on day 5 (*n* = 8 per group, where each point represents a culture dish, combined from at least 4 mice). On day 5 of culture BMDMs were incubated for 1 h with 1 µg/ml *E.coli* particles in vitro and percentage CD206 positivity was assessed. Ordinary one way ANOVA with Bonferroni correction, WT IL-4 vs ACKR2-/- IL-4 *p* < 0.0001, WT with eosinophils and IL-4 vs ACKR2-/- with eosinophils and IL-4, *p* = 0.002, ACKR2-/- eosinophils and IL-4 vs ACKR2-/- IL-4, *p* < 0.0001. **d** Percentage of pHRODO *E.coli* particle positive BMDMs, with and without eosinophil treatment on day 0 of culture (*n* = 8 per group, except ACKR2-/- control *n* = 7), ordinary one way ANOVA with Bonferroni correction, WT vs ACKR2-/-, *p* < 0.0001, WT with eosinophils vs ACKR2-/- with eosinophils, *p* = 0.0141, ACKR2-/- vs ACKR2-/- with eosinophils, *p* = 0.0041. **e** Intracellular **i** ALOX15 and **ii** TGF-β production after 18 h co-culture of FACS sorted monocytes and eosinophils from WT mice (no eosinophils, Alox15, *n* = 8, TGF-β, *n* = 7, eosinophils and monocytes with contact, Alox15, *n* = 7, TGF-β, *n* = 8), without contact, *n* = 4). Ordinary one way ANOVA with Bonferroni correction, Media vs eosinophil contact, Alox-15, *p* = 0.0009, TGF-β, *p* = 0.0002, media vs eosinophils without contact, Alox-15, *p* < 0.0001, TGF-β, *p* = 0.0275. **f** Number of **i** eosinophils in the bone marrow, two-tailed Mann-Whitney test, *p* = 0.0043, **ii** SPMs, unpaired two-tailed *t*-test, *p* = 0.0432, and **iii** LPMs, two-tailed, Mann-Whitney, *p* = 0.0173, in the peritoneal cavity of WT mice challenged 3 times, every 2 days with 20 µg isotype control (black circles, IgG2a, *n* = 5) or anti-Siglec-F antibody (white circles, α-SiglecF, *n* = 6). Significantly different results are i*n*dicated (*, *p* ≤ *e* 0.05, **, *p* ≤ 0.01, ***, *p* ≤ 0.001, ****, *p* ≤ 0.0001). Error bars represent SEM. Source data are provided as a Source Data file.

We have shown that eosinophil retention in the bone marrow appears to be ACKR2-dependent and CCL11-mobilisable (Fig. 6g). Therefore, we next investigated whether eosinophils and monocytes interact within the bone marrow. Firstly, we used iREP reporter mice, which we have recently generated, where each of the inflammatory chemokine receptors CCR1, 2, 3 and 5 are discretely labelled^39^. Flow cytometric analysis of bone marrow cells from iREP mice revealed that 80% of CCR2-expressing cells are Ly6C+ monocytes, and 97% of CCR3 expressing cells are SiglecF+ eosinophils (Supplementary Fig. 15). We were able to visualise CCR2+ and CCR3+ cells in close proximity within the bone marrow (Fig. 7a), which are likely to represent CCR2+ monocytes and CCR3+ eosinophils.

To investigate whether the ACKR2 ligand CCL11 affects interactions between CCR2+ and CCR3+ cells, 1 µg CCL11 was injected intravenously, and 5 h later bone marrow was obtained for imaging analysis. CCR2 + /CCR3+ cell interactions were counted in defined regions of interest. When CCL11 was injected into the bloodstream, the number of CCR2 + /CCR3+ cell interactions within the bone marrow decreased (Fig. 7b). Due to the short half-life of chemokines in vivo, it is not possible to fully deplete eosinophils in the bone marrow^38^.

To investigate if eosinophils promote monocyte to macrophage differentiation, we added FACS-sorted WT eosinophils on day 0, to WT and ACKR2-/- bone marrow cultures in vitro. Treatment with eosinophils alone did not alter CD206 expression on macrophages, however when stimulated with IL-4, eosinophil treated BMDMs from ACKR2-/- mice were able to respond to IL-4 at increased levels (Fig. 7c). In addition, we have shown that ACKR2-/- BMDMs have a phagocytic defect which results in a higher percentage of ECP+ cells after challenge, due to incomplete breakdown of the pHRODO particles (Fig. 2giii). Treatment with eosinophils on day 0 restored the ability of BMDMs from ACKR2-/- mice to destroy ECP after phagocytosis (Fig. 7d).

We have identified transcriptional changes in monocytes from the bone marrow of ACKR2-/- mice, where monocytes had less exposure to eosinophils, such as reduced transcription of TGFB1 and ALOX15 (Fig. 5aiii, iv). To investigate whether increased exposure to eosinophils was able to drive expression of these genes in monocytes, we co-cultured FACS sorted monocytes and eosinophils for 18 h in vitro. Upon co-culture with eosinophils, monocytes produced more TGFB1 and ALOX15, detected by intracellular flow cytometry (Fig. 7ei, ii). This process was not contact dependent, as eosinophils cultured in a transwell system were also able to drive the gene expression changes in monocytes (Fig. 7ei, ii). To investigate the effect of eosinophils on macrophage differentiation, eosinophils were depleted in WT mice using anti-Siglec-F antibody. Eosinophils were depleted in the bone marrow using this protocol (Fig. 7fi). In the absence of eosinophils there was a marked decrease in both SPMs and LPMs within the peritoneal cavity indicating that eosinophils are important for macrophage differentiation in vivo (Fig. 7fii, iii).

## Discussion

Monocyte-derived macrophages are a key component of the immune response, and are found in most tissues throughout the body^3^. Monocytes are recruited from the bone marrow and differentiate into macrophages at distal sites. Here we describe, a previously unknown role for eosinophils, classically known as mediators of type 2 immunity, in educating monocytes within the bone marrow, prior to recruitment into tissues.

**Fig. 8:**
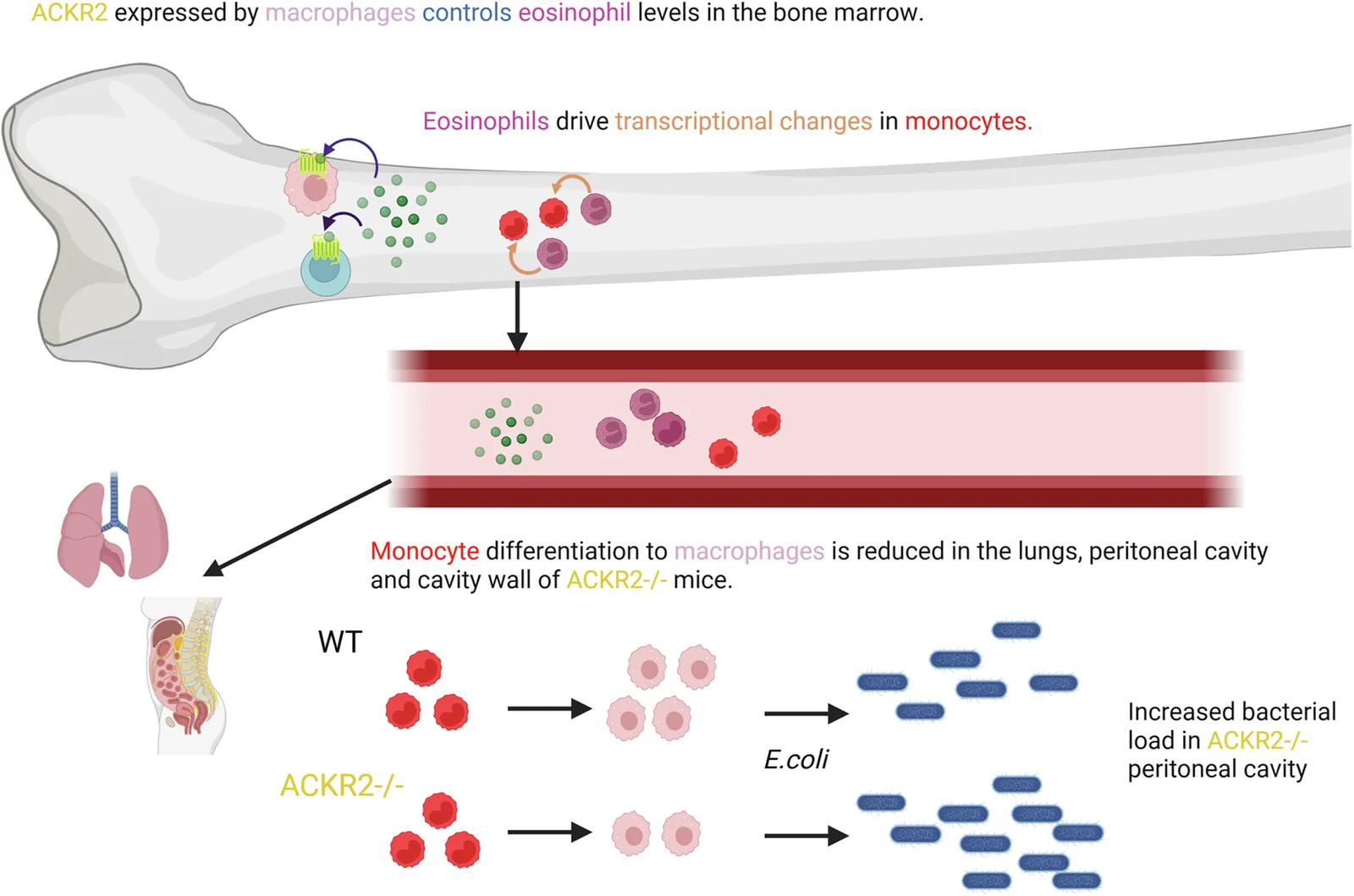
Schematic to illustrate how ACKR2 promotes monocyte to macrophage differentiation by contributing to the regulation of eosinophils in the bone marrow. Created in BioRender. Wilson, G. (2026) https://BioRender.com/kh539gn.

We demonstrate interactions between eosinophils and monocytes in the bone marrow, indirectly controlled by the atypical chemokine receptor ACKR2. In the absence of ACKR2, eosinophils and monocytes have less opportunity to interact within the bone marrow, and changes in monocyte gene expression were observed. Monocytes from ACKR2-/- mice are able to travel to the tissues in equivalent numbers, but their ability to differentiate into macrophages is fundamentally altered, in the lung, peritoneal cavity and peritoneal wall. This mechanism has been illustrated as a schematic in Fig. 8. In this study, we did not observe changes in any of the other tissues we investigated, such as the skin, omentum, or the spleen. Surprisingly, we also did not observe any reduction in pleural cavity SPMs, which are similar to peritoneal SPMs^25^, suggesting that these cells have an altered differentiation pathway. Importantly, BMDMs generated from ACKR2-/- mice exhibit the key differences observed in tissue specific macrophages, such as reduced MHCII expression, impaired breakdown of pHRODO *E.coli* particles, and diminished CD206 expression. This suggests that the effect on monocyte derived macrophages observed in ACKR2-/- mice at rest is stable ex vivo and may not be limited to the peritoneal cavity and lung.

Reduced differentiation of monocytes to macrophages in the peritoneal cavity of ACKR2-/- mice has clear functional implications, exemplified by increased bacterial load in the peritoneal cavity during infection. It is notable that there is a large increase in *E.coli* burden in the ACKR2-/- peritoneal cavity but not in the ACKR2-/- peritoneal cavity wall, a key site of bacterial clearance, which could explain why there is no difference in weight change between WT and ACKR2-/- animals during infection. This may suggest there is a functional difference between the phagocytic capacity of SPMs in the cavity wall and within the peritoneal cavity, or that macrophages phagocytose bacteria differently in the wall environment.

Our transcriptional analysis of peritoneal cavity SPMs revealed highly specific gene changes, which may contribute to the reduced functionality of macrophages from ACKR2-/- mice during peritoneal infection. SPMs from ACKR2-/- mice have reduced transcription of CTSE and Chil1 (Fig. 5bi). CTSE-/- macrophages have decreased bactericidal activity, and CTSE-/- mice are highly susceptible to infection with *Staphylococcus aureus* and *Porphyromonas gingivalis*^40,41^. Similarly, Chil1-/- mice are more susceptible to *Pseudomonas aeruginosa* infection^42^. Additionally, the formation of cellular aggregates of resident macrophages and mesothelia within the peritoneum is important for bacterial killing^27^. Here, we observed reduced numbers of resident macrophages in the wall of ACKR2-/- mice during infection, which may contribute to insufficient bacterial elimination. ACKR2 has previously been shown to be important during infection with *Mycobacterium tuberculosis*, attributed to increased chemokine and cytokine production in ACKR2-/- mice^43^. Here, we reveal a lung macrophage defect in ACKR2-/- mice, which may have contributed to increased pathology. In a caecal ligation and puncture (CLP) sepsis model, there is also evidence of a protective role of ACKR2^44^.

Previously, expression of ACKR2 by osteoclasts and osteoblasts has been demonstrated during bone remodelling^45^. Here we show for the first time, ACKR2 expression by macrophages in the bone marrow, at rest. Previously, ACKR2 expression has mainly been identified in stromal populations throughout the body, such as LECs and fibroblasts, or by peritoneal B1 cells^9^. Notably, several human GWAS studies have revealed SNPs in the ACKR2 gene, which are associated with monocyte (rs2228467, rs1366045, rs136046, rs219224, and rs2228468) and eosinophil counts (rs2228467, rs7433284, rs9872570), lending further support to the idea that ACKR2 plays an important role in controlling human monocyte and eosinophil interactions^46–50^.

Several studies have revealed a role for eosinophils in polarising macrophages towards an M2 or alternatively activated phenotype^51–53^. The transfer of eosinophils during collagen-induced arthritis increased the proportion of CD163+ macrophages and TGF-β production in arthritic joints^51^. In adipose tissue, alternative macrophage activation was impaired in the absence of eosinophils^53^. Here, we reveal an ACKR2-dependent control mechanism and show that eosinophils and monocytes are in close proximity within the bone marrow, and that eosinophils transcriptionally alter monocytes, a change necessary for full CD206 expression in response to IL-4 stimulation. Notably, the co-culture of eosinophils and monocytes was sufficient to recapitulate transcriptional changes observed in ACKR2-/- monocytes. Our transcriptomic data alone do not definitively show that eosinophils are responsible for the differences between WT and ACKR2-/- macrophages. However, the co-culture of eosinophils and monocytes led to altered expression of two key genes, in keeping with our hypothesis. These were significant, but small effects; therefore, the biological significance is unclear. In addition, there are many other transcriptional changes which we have not investigated in this study. We have shown that this process does not require direct contact between the cell types, which suggests that secreted factors or degranulation products may mediate the effect. Future work will be important to determine the nature of the factor(s) responsible.

We used antibody-based eosinophil depletion to investigate the effect of eosinophil deficiency in this model. However, a caveat of this approach is that eosinophil-deficient mice are known to exhibit gut dysbiosis and reduced recruitment of intestinal macrophages^54^, which may impact the peritoneal cavity at rest and during infection. For example, LPMs have been shown to leave the peritoneum to contribute to the repair of intestinal inflammation^55^, thereby extending the immune dysfunction from the intestine to the peritoneal cavity.

This study demonstrates a mechanism whereby the atypical chemokine receptor, ACKR2 maintains a stable level of eosinophils in the bone marrow niche to facilitate key eosinophil and monocyte interactions. Without these interactions, monocytes undergo transcriptional alterations and are impaired in their ability to differentiate into macrophages. In the absence of ACKR2, many transcriptional changes were observed in monocytes, including a number of key genes, such as *CCL5*, which likely contribute to the reduced levels of differentiation to macrophages. Further, these macrophages are impaired in their ability to phagocytose bacteria during infection. Taken together, this study demonstrates, for the first time, that eosinophils play a key role in monocyte education in the bone marrow, thereby contributing to defence against bacterial infection.

## Methods

### Study design

The objective of this study was to reveal how ACKR2 regulated the number of macrophages in different tissues and the response to bacterial infection. A combination of in vitro and in vivo approaches have been used. All mouse experiments were carried out using age and sex matched animals. Unless otherwise stated, each point represents an individual mouse. The number of replicates, *n* has been reported for each experiment. At least three biological replicates per experimental group were used. Required sample size was calculated based on previous similar experiments.

### Ethics statement

Animal experiments were carried out in accordance with UK Home Office regulations (Project Licences PP6655603 and PP1443954). All experiments conformed to animal care and welfare protocols, approved by the Animal Welfare Ethical Review Body at the University of Glasgow. All mice were housed in individually ventilated cages (IVCs) within a controlled animal facility. Animals were maintained on a 12 h light/dark cycle, with lights on at 07.00 and off at 19.00. Environmental conditions were maintained at a temperature of 19 °C–23 °C and a relative humidity of 55% (±10%). Animals were group-housed with appropriate environmental enrichment. Wild-type (WT) C57BL/6 J mice were purchased from Charles River Laboratories. CD45.1^56^, ACKR2-/-^57^, iREP^39^, and CCR3-/-^58^ mice were bred in the specific pathogen-free facility of the University of Glasgow Wolfson Research Facility (WRF). To generate Ackr2flox mice, a conditional allele of Ackr2 was created by inserting loxP sites flanking exon 3 of the Ackr2 locus using CRISPR/Cas9-mediated genome editing in C57BL/6 N zygotes. Mouse production was performed by Biocytogen. Founder mice were genotyped by PCR and bred to establish the Ackr2flox line. This line was subsequently backcrossed to C57BL/6 J mice for more than five generations before use in experiments. To investigate the role of ACKR2 in myeloid cells, Ackr2flox/flox mice were crossed with Lyz2-Cre transgenic mice (kindly provided by Prof. Kevin Ryan, CRUK Scotland Institute). ACKR2 flox x LysM cre mice were bred with littermate controls. Other strains were bred as homozygous colonies and age and sex matched with appropriate WT controls.

### Flow cytometry

Flow cytometry and cell sorting experiments were carried out according to established guidelines^59^. Dead cells were excluded using Fixable Viability Dye eFluor 506 (Thermo Fisher Scientific). Cell suspensions were blocked with TruStain FcX (Biolegend) at 1:200. Antibodies were used at a dilution of 1 in 200, with the exception of NK1.1 (S17016D), which was used at 1 in 100, in FACS buffer (PBS, 1% foetal bovine serum (FBS), and 2 mM EDTA (Thermo Fisher Scientific)). Anti-mouse antibodies of the following clones were used on various fluorochromes: CD45 (30-F11), CD11b (M1/70), F4/80 (BM8), Siglec F (S17007L), Ly6C (HK1·4), CD11c (N418), MHCII (M5/114·15·2), CD206 (C068C2), CD5 (53-7.3), CD23 (B3B4), CD45.1 (A20), CD45.2 (104), CD80 (2D10), Sca1 (D7), c-kit (2B8), CD34 (MEC14.7), TGF-β1 (TW7-16B4), CD226 (TX42.1), MerTK (CB10C42), NK1.1 (S17016D), TCR γ/δ (GL3), CD41 (MWReg30) and CD61 (2C9.G2) were obtained from Biolegend. Ly6G (1A8), CD8 (53-6.7), CD4 (GK1.5), CD19 (1D3) were obtained from BD Biosciences. Lyve-1- Alexa Fluor 488 (223322) was obtained from R&D Systems, and ALOX15 Alexa Fluor 488 (AA 581–662) from Antibodies online. Cell suspensions were washed with FACS buffer and fixed with 2% PFA. Intracellular staining was carried out using the Cyto Fast Fix Perm buffer set (Biolegend), as described in the manufacturer’s instructions. Flow cytometry was performed using a Fortessa or Celesta (BD Biosciences) and analysed using FlowJo V10. FACS sorting was carried out using a FACSaria III (BD Biosciences). For depletion

### Cell isolation

Peritoneal lavage was carried out by injecting 10 ml PBS containing EDTA into the peritoneal cavity of euthanized mice and collecting the fluid with a syringe. Lavage of the pleural cavity space was carried out by injecting 2 ml PBS containing 2 mM EDTA. Bone marrow cells were obtained by dissecting the hind limbs, removing the muscle, cutting the femur and tibia bones and flushing with 1 ml PBS containing EDTA. Red blood cells (RBC) were lysed in 1 mL RBC lysis buffer (Invitrogen) for 1 min, and then quenched in 10 ml PBS. Blood was collected by cardiac puncture into a 1 ml syringe containing 50 µl 0.5 M EDTA. Cells were washed in FACS buffer and analysed by flow cytometry.

### Tissue digestion

Lungs were removed and chopped finely, before digestion with 0.8 mg/mL Dispase II, 0.2 mg/mL Collagenase P, and 0.1 mg/mL DNase I (Roche) in HBSS shaken at 800 rpm, at 37 °C for 45 min. Lungs were filtered through a 40 µm EASYstrainer mesh (Greiner Bio-One), and RBC were lysed. Peritoneal cavity wall was excised and chopped finely, before digestion with 1 ml of Liberase at 0.44 Wunch units per sample, for 1 h at 37 °C, 1000 rpm using a Thermoshaker. Cell suspension was filtered through a 100 µm EASYstrainer. Mice were shaved and an approx. 5 cm^2^ piece of dorsal skin was excised and chopped finely, before digestion with HBSS containing collagenase D (1 mg/mL Roche), Dispase II (0.5 mg/mL, Roche) and DNase I (0.1 mg/mL, Roche). Cell suspensions were obtained from the spleen by filtering through a 70 µm EASYstrainer mesh (Greiner One). The omentum was removed and chopped finely before digestion with 2 mg/ml Collagenase type I (Roche) and 4% BSA (Invitrogen) in DMEM. Samples were shaken at 800 rpm, 37 °C for 1 h. The omentum was then passed through a 40 µm EASYstrainer mesh (Greiner One). For each tissue, RBC were lysed in 1 mL RBC lysis buffer (Invitrogen) for 1 min, and washed with 10 ml PBS.

### BMDM generation

Bone marrow cells were isolated from the femurs of at least 4 WT and ACKR2-/- mice. Cells were grown in media consisting of 433 ml GMEM (Glasgow Minimum Essential Medium), 50 ml FBS (foetal bovine serum), 20 ng/ml mouse M-CSF Recombinant Protein (Peprotech), 5 ml non-essential amino acids (Gibco), 5 ml L-glutamine (Sigma-Aldrich), 5 ml sodium pyruvate (Sigma-Aldrich), 0.1 mM β-mercaptoethanol (Gibco) and 1 ml of Primocin (Invivogen). Bone marrow was collected, unwanted debris removed using a 70 μm filter, and centrifuged at 300 g for 5 min. The supernatant was discarded, the cell pellet was resuspended in 1 ml of ACK lysis buffer (Thermo Fisher Scientific) and incubated at room temperature for 1 min. Next, cells were centrifuged at 300 g for 5 min and resuspended in 1 ml of BMDM media. Cell numbers were determined using 1:1 dilution with Trypan blue, and seeded at a density of 5 x 10^5^ in 35 mm Petri dishes with BMDM media. On day 3, media was replaced. BMDMs were stimulated on day 5 with either IL-4 (Biolegend) at 20 ng/ml, or LPS at 10 ng/ml (eBioscience). In some experiments, 20,000 FACS-sorted eosinophils were added on day 0 at a ratio of 1:25 (eosinophil: monocyte).

### Monocyte and eosinophil co-culture

Monocytes and eosinophils were purified by FACS sorting using the FACSAria III (BD Biosciences), and cultured together for 18 h at a ratio of 10:1. Millicell 96 well Plate with 0.4 µm pore size (Millipore, Sigma) was used to assess whether gene expression changes were contact dependent. After 18 h cells were stained and assessed by flow cytometry.

### Peritoneal bacterial infection

Mice between 7 and 9 weeks of age were injected intraperitoneally (i.p.) with 5 × 10^6^ CFU of *E*. *coli* (UPEC isolate CFT073)^60^. Bacteria were grown overnight in Luria-Bertani (LB) medium, then sub-cultured the following day and grown to log phase for injection (OD_600_ = 0·5, 5 × 10^8^ CFU/ml). Mice were monitored for weight loss and clinical signs, culled 18 h later, and lavages were collected. For CFU analysis, 1 ml of the lavage fluid was centrifuged at 13,000 g and resuspended in 50 µl for serial dilution. To determine CFU values from the spleen, peritoneal cavity wall and liver, tissue was disrupted using stainless steel beads and a Tissuelyser LT (Qiagen) for 10 min at a rate of 50 s^−1^. CFU were determined by plating serial dilutions on LB agar. For macrophage depletion, 150 µl Clophosome Liposomes containing either PBS or Clodronate (Stratech) were injected i.p into mice on day 0. On day 7 mice were injected i.p with 5 × 10^6^ CFU of *E*. *coli* (UPEC isolate CFT073). 18 h later mice were culled and peritoneal lavage was caried out.

### Sterile inflammation of the peritoneal cavity

Mice were injected i.p. with either 200 µl PBS or 200 µg of pHRODO Bioparticles in 200ul PBS (*E.coli* K-12 strain, Thermo Fisher Scientific). After 18 h, mice were culled, peritoneal lavage was collected and processed for cellular analysis.

### Adoptive transfer experiments

Monocytes were purified from bone marrow cells isolated from four mice, by magnetic sorting using the EasySep™ Mouse Monocyte Isolation Kit (Stem Cell Technologies), as described in the manufacturer’s instructions. 1 × 105 monocytes were injected i.p. into mice, and cells were collected 2 days later by peritoneal lavage.

### Macrophage depletion

150 µl Clophosome Liposomes containing either PBS or clodronate (Stratech) were injected intraperitoneally into mice on day 0. Mice were culled, and peritoneal lavage was carried out after 1 and 4 weeks.

### Eosinophil depletion

WT mice were injected i.p with 20 µg anti-mouse Siglec-F (238047) or isotype control (rat IgG2a, 54447) (Biotechne) three times, every 2 days^61^. Mice were culled on day 5 and peritoneal lavage was performed.

### Imaging

Bone marrow from iREP mice were visualised at 20 x magnification on an Zeiss Axioimager M2.

Samples were prepared using a tape strip methodology developed by Dr Tadafumi Kawamoto^62^. The peritoneal wall was fixed using 4% PFA overnight, then placed into 30% sucrose overnight before being frozen in OCT. ACKR2 expression in the peritoneal wall was detected by RNAscope in situ hybridisation using a specific probe, as described in the manufacturer’s instructions^16,17^. Co-staining with Lyve-1 Alexa Fluor 488-labelled antibody (R&D systems) was carried out at 1:200 dilution in 1% BSA in PBS containing 0.1% Tween-20 (Sigma), overnight at 4 °C.

### Protein samples

Peritoneal wall tissue was frozen in liquid nitrogen, crushed using a mortar and pestle, and resuspended in dH_2_O containing protease inhibitors (Pierce). Plasma was obtained from blood samples collected in 50 µl 0.5 M EDTA, and centrifuged at 1500 g for 10 min at 4 °C. Peritoneal lavage cells were centrifuged at 400 g and supernatants were removed for analysis. Cell pellets from the peritoneal lavage and bone marrow were lysed using Lysis 2 buffer (R&D systems), containing HALT protease inhibitors (Thermo Fisher Scientific). Protein concentrations were determined using a Magnetic Luminex Multiplex assay (custom designed, R&D Systems), as described in the manufacturer’s instructions, and read using a Bio-Rad Luminex-200 machine. CCL13 was detected using an ELISA (Antibodies Online), as described in the manufacturer’s instructions. Data was normalised to the total protein concentration of tissue samples, as determined by a BCA assay (Pierce).

### CCL22 uptake assay

ACKR2 expression was determined by comparing uptake of Alexa Fluor 647 (AF647) labelled CCL22 by WT and ACKR2-/- cells^17,19,30^. In vivo ACKR2 expression was assessed by injecting either PBS or 0.5 µg of AF647 CCL22 i.p. into WT and ACKR2-/- animals. 1 h later, cells were collected by peritoneal lavage stained and analysed by flow cytometry. In vitro uptake assays were performed by incubating WT and ACKR2-/- cells with 0.25 µg/ml AF647 CCL22, at 37 °C and 5% CO_2_ for 1 h. Cells were then stained at 4 °C and analysed by flow cytometry.

### Transcriptional analysis

Monocytes from the bone marrow and SPMs from the peritoneal cavity were FACS sorted using a FACSAria III (BD Biosciences) directly into RLT buffer (Qiagen). For each individual sample, cells were sorted from 5 mice. RNA was extracted using an RNeasy micro kit with on column DNase treatment (Qiagen) as described in the manufacturer’s instructions. RNA sequencing was carried out by Novogene for RNA QC, mRNA library preparation (poly-A enrichment) and Illumina Sequencing (PE150) with SMARTer sequence amplification. Bioinformatic analysis was carried out using Searchlight 2 (v2.0.3)^63^. Quantitative real time PCR analysis was carried out using primers for ACKR2, forward: AGGAAGGATGCAGTGGTGTC and reverse: CGGAGCAAGACCATGAGAAG and GAPDH forward: ATGTGTCCGTCGTGGATCTGACG and reverse: GTTGCTGTTGAAGTCGCAGGAG^31^.

### Statistical analysis

Data were analysed using GraphPad Prism version 10.6.0. Normality was assessed using Shapiro–Wilk and Kolmogorov–Smirnov tests. Normally distributed data was analysed using two-tailed, unpaired t-tests or ordinary one way ANOVA with Bonferroni correction. Where data was not normally distributed, Mann–Whitney tests or Kruskal-Wallis tests with Dunn’s correction were used. Significance was defined as *, *p* ≤ 0.05, **, *p* ≤ 0.01, ***, *p* ≤ 0.001, ****, *p* ≤ 0.0001). Error bars indicate standard error of the mean (SEM).

### Reporting summary

Further information on research design is available in the Nature Portfolio Reporting Summary linked to this article.

## Supplementary information

Supplementary Information

Reporting Summary

Transparent Peer Review file

## Source data

Source Data

Source Data - Supplementary

## Data Availability

All data supporting the findings of this study are available within the paper and the Supplementary Information. RNA sequencing data is available on Genbank, GEO accession number GSE334547. Source data is available with this paper. Source data are provided with this paper.

## Code availability

No custom code has been used in this study.
