# Supplementary material for: ACKR2 aids the regulation of bone marrow eosinophils to promote monocyte differentiation and anti-bacterial immunity
Source: Nat Commun. 2026 Jul 22;17:8954. doi: 10.1038/s41467-026-75964-z (PMC13503710; doi:10.1038/s41467-026-75964-z)
Supplement: Supplementary file 1 [file 41467_2026_75964_MOESM1_ESM.pdf]

Supplementary Figures

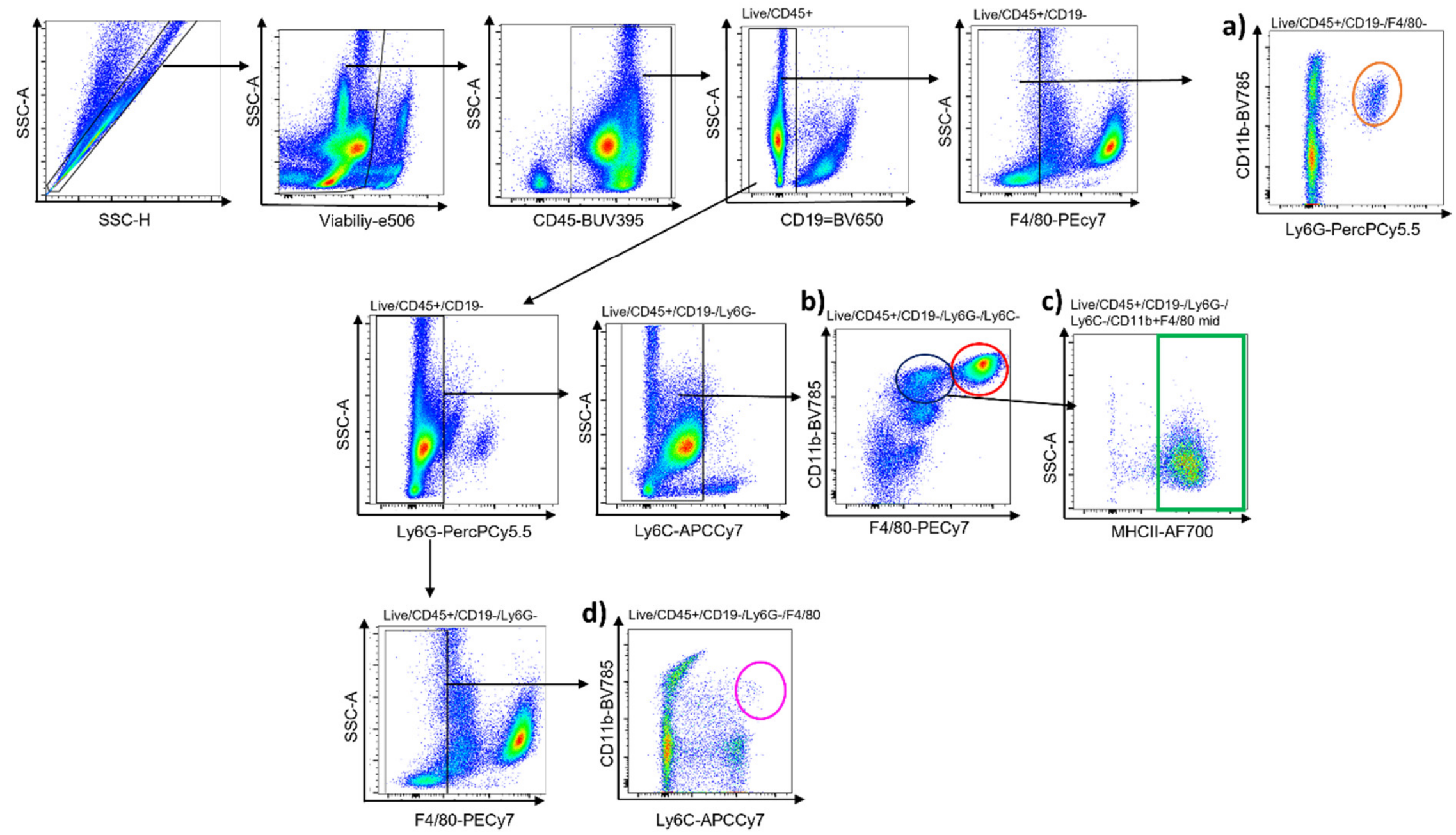

Supplementary Figure 1: Gating strategy to define cell types in the peritoneal cavity. Flow cytometry was carried out to identify cell types in the peritoneal cavity. Single cells were gated, dead cells were excluded, and CD45+ and CD19- cells were gated. **a)** Neutrophils were identified by further gating on F4/80- cells and defined as CD11b+, Ly6G+ (orange). **b)** Resident peritoneal macrophages, LPMs were defined as CD11b+, F4/80 high (red). **c)** Monocyte derived SPMs were further gated as F4/80 mid and MHCII positive (green). **d)** Monocytes were defined as Ly6G-, F4/80-, CD11b+, Ly6C+ (pink).

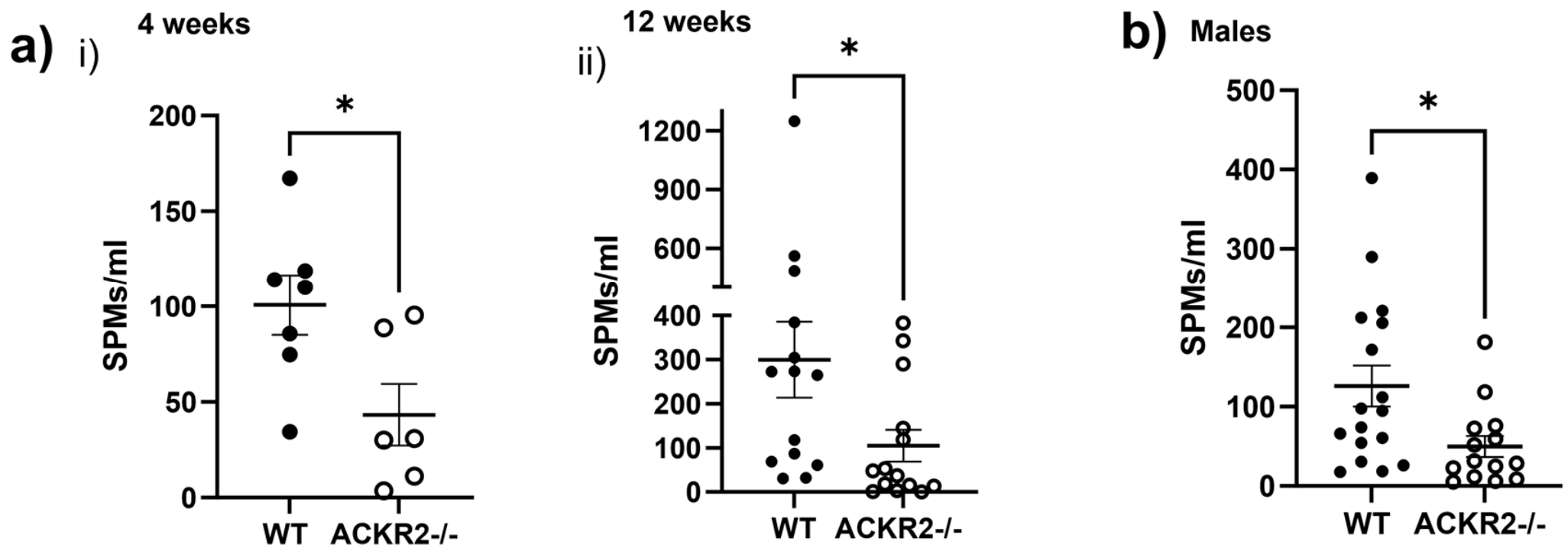

Supplementary Figure 2: SPMs are reduced at different ages and in male mice.
a) Flow cytometry was carried out to determine the number of SPMs in WT (black circles) and ACKR2-/- (white circles) female **i)** 4 week old (WT, *n*=7, ACKR2-/-, *n*=6), unpaired, two-tailed *t* test, $p = 0.0271$ and **ii)** 12 week old mice (*n*=14, per group), two-tailed Mann-Whitney, $p = 0.0186$. **b)** 8 week old males (WT, *n*=17, ACKR2-/-, *n*=14), two-tailed Mann-Whitney, $p = 0.0147$. Significantly different results are indicated (*, $p < 0.05$). Error bars represent SEM.

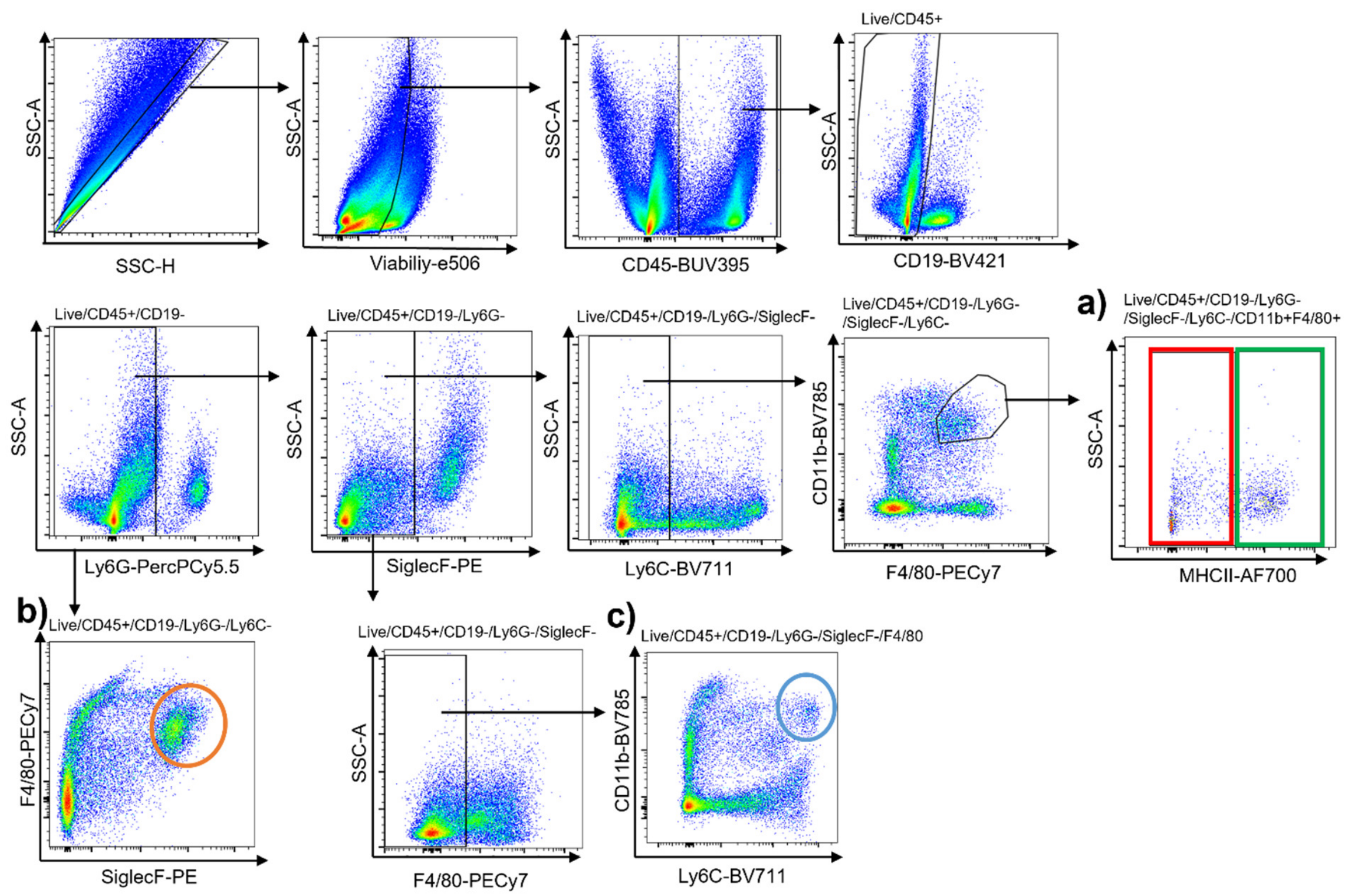

Supplementary Figure 3: Gating strategy for cell types in the lung. Flow cytometry was carried out to identify cell types in the lung. Single cells were gated, dead cells were excluded, and CD45+, CD19- cells and Ly6G- were gated. **a)** Interstitial macrophages were defined as SiglecF-, Ly6C-, CD11b+ and F4/80+, and further divided into MHCII negative (red) and positive (green) populations. **b)** Resident alveolar macrophages were defined as F4/80 low SiglecF+ (orange). **c)** Monocytes were defined as SiglecF-, F4/80-, CD11b+, Ly6C+ (blue).

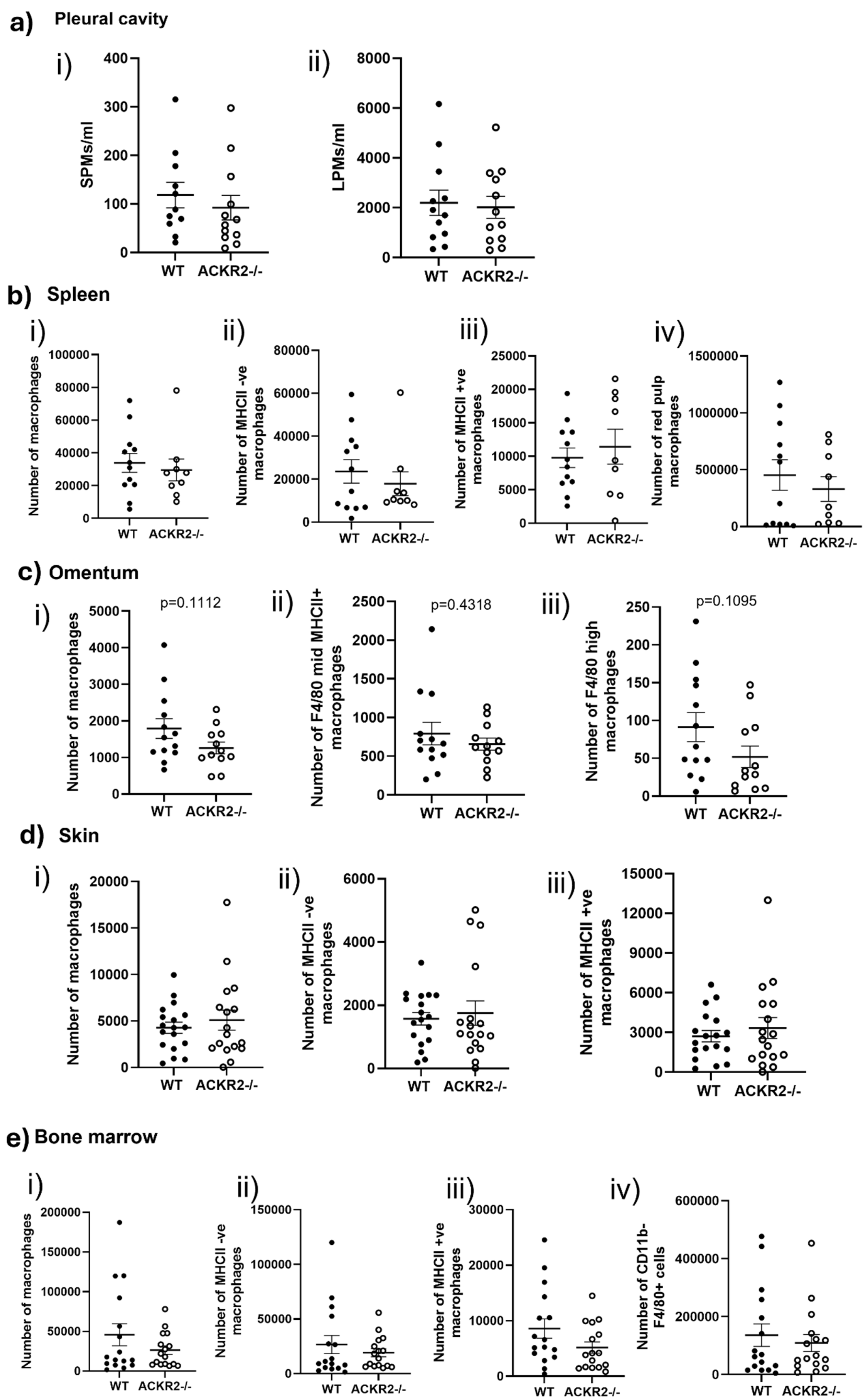
a) Pleural cavity
i)
SPMs/ml
0
100
200
300
400
WT
ACKR2-/-
ii)
LPMs/ml
0
2000
4000
6000
8000
WT
ACKR2-/-
b) Spleen
i)
Number of macrophages
0
20000
40000
60000
80000
100000
WT
ACKR2-/-
ii)
Number of MHCII -ve macrophages
0
20000
40000
60000
80000
WT
ACKR2-/-
iii)
Number of MHCII +ve macrophages
0
5000
10000
15000
20000
25000
WT
ACKR2-/-
iv)
Number of red pulp macrophages
0
500000
1000000
1500000
WT
ACKR2-/-
c) Omentum
i)
p=0.1112
Number of macrophages
0
1000
2000
3000
4000
5000
WT
ACKR2-/-
ii)
p=0.4318
Number of F4/80 mid MHCII+ macrophages
0
500
1000
1500
2000
2500
WT
ACKR2-/-
iii)
p=0.1095
Number of F4/80 high macrophages
0
50
100
150
200
250
WT
ACKR2-/-
d) Skin
i)
Number of macrophages
0
5000
10000
15000
20000
WT
ACKR2-/-
ii)
Number of MHCII -ve macrophages
0
2000
4000
6000
WT
ACKR2-/-
iii)
Number of MHCII +ve macrophages
0
3000
6000
9000
12000
15000
WT
ACKR2-/-
e) Bone marrow
i)
Number of macrophages
0
50000
100000
150000
200000
WT
ACKR2-/-
ii)
Number of MHCII -ve macrophages
0
50000
100000
150000
WT
ACKR2-/-
iii)
Number of MHCII +ve macrophages
0
10000
20000
30000
WT
ACKR2-/-
iv)
Number of CD11b- F4/80+ cells
0
200000
400000
600000
WT
ACKR2-/-

Supplementary Figure 4: Macrophage numbers are not significantly altered in the absence of ACKR2 in other tissues within the body. Flow cytometry was used to determine the number of macrophages within the single cell, live, CD45+, CD19-, Ly6G-, Ly6C- populations in various tissues. **a)** pleural cavity, **i)** SPMs, and **ii)** LPMs, WT, *n*=11 (black circles), ACKR2-/-, *n*=12 (white circles), were gated as in the peritoneal cavity (Supplementary Figure 1). **b)** Spleen macrophages were defined as **i)** CD11b+ F4/80+ and further divided into **ii)** MHCII negative and **iii)** MHCII positive. **iv)** Resident red pulp macrophages were defined as CD11b-F4/80+, WT, *n*=12, ACKR2-/-, *n*=9. **c)** In the omentum macrophages were defined as **i)** CD11b+ F4/80+, **ii)** CD11b+F4/80 mid MHCII positive, and **iii)** CD11b+F4/80 high, WT *n*=13, and ACKR2-/- *n*=12. **d)** Skin macrophages were defined as **i)** CD11b+ F4/80+ and further divided into **ii)** MHCII negative and **iii)** MHCII positive, WT *n*=18, and ACKR2-/- *n*=17. **e)** Bone marrow macrophages were defined as **i)** CD11b+ F4/80+ i**i)** MHCII negative and **iii)** MHCII positive, **iv)** CD11b-F4/80+, *n*=16 per group.

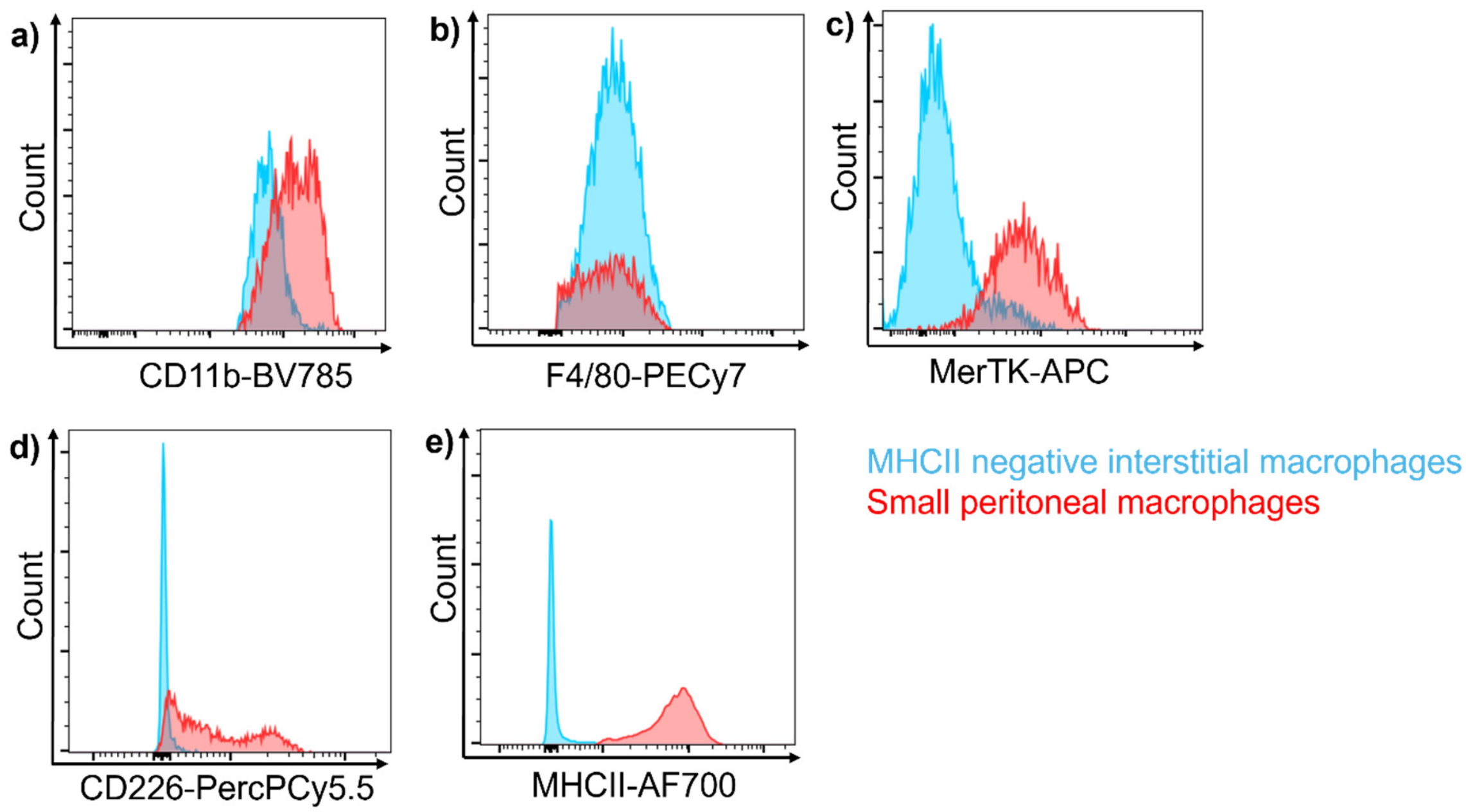

Supplementary Figure 5: Surface marker expression is distinct between ACKR2 controlled macrophage populations. Flow cytometry was used to determine the expression of macrophage surface markers **a)** CD11b, **b)** F4/80, **c)** MerTK, **d)** CD226, and **e)** MHCII. Representative plots of MHCII negative interstitial macrophages are shown in blue and small peritoneal macrophages in red.

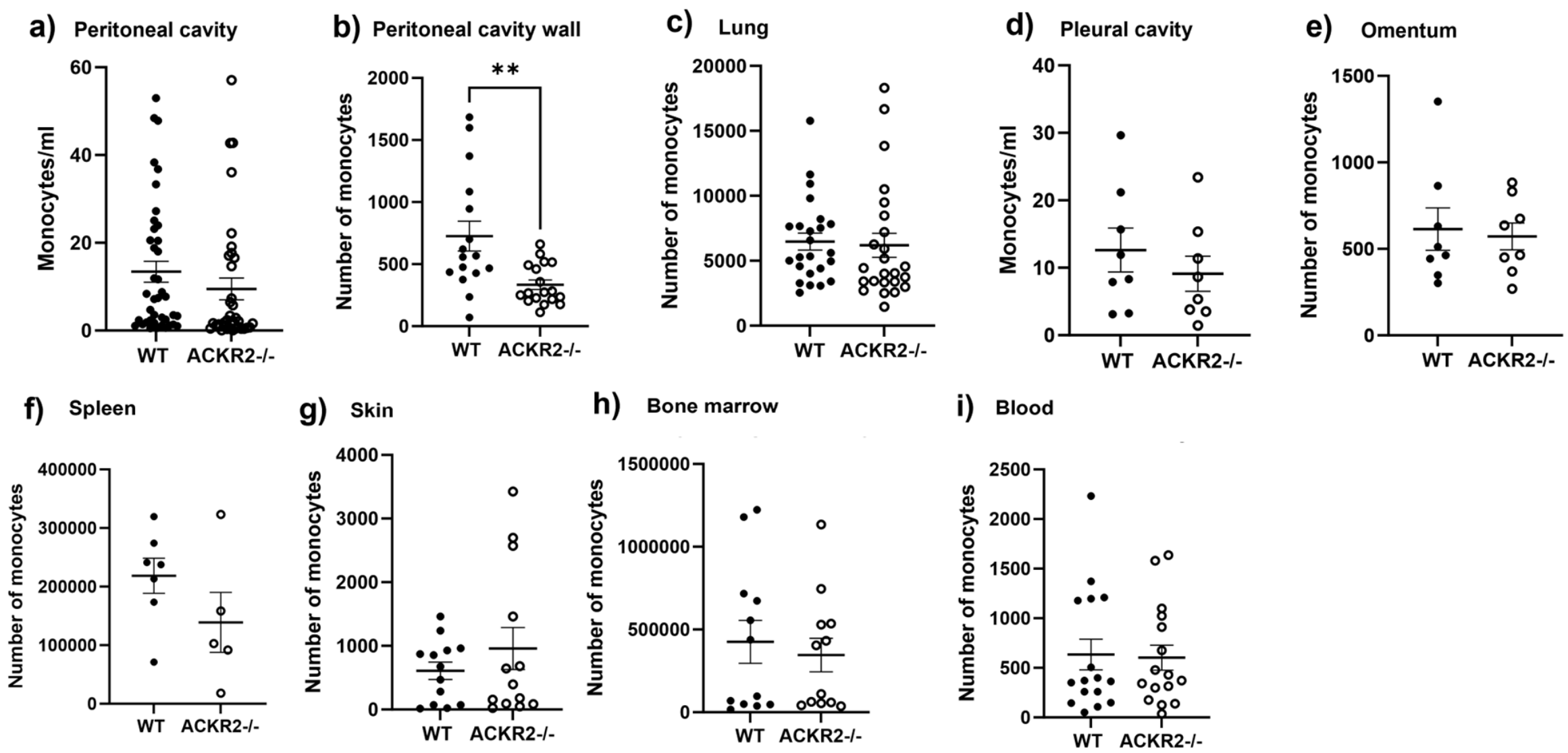

Supplementary Figure 6: Monocyte numbers throughout the body. Monocytes were defined by flow cytometry as single, live, CD45+, CD19-, Ly6G-, CD11b+ and Ly6C+ cells. Numbers were determined in WT (denoted by black circles) and ACKR2-/- (white circles) mice, in the **a)** peritoneal cavity (WT, *n*=40, ACKR2-/-, *n*=35), **b)** peritoneal cavity wall (WT, *n*=16, ACKR2-/-, *n*=18), unpaired, two-tailed *t* test, p = 0.0044. **c)** lung (*n*=24, per group), **d)** pleural cavity, (*n*=8, per group) **e)** omentum (*n*=8, per group), **f)** spleen (WT, *n*=7, ACKR2-/-, *n*=5), **g)** skin (*n*=13, per group), **h)** bone marrow (*n*=12, per group), and **i)** blood (*n*=16, per group). Significantly different results are indicated (*, $p < 0.05$). Error bars represent SEM.

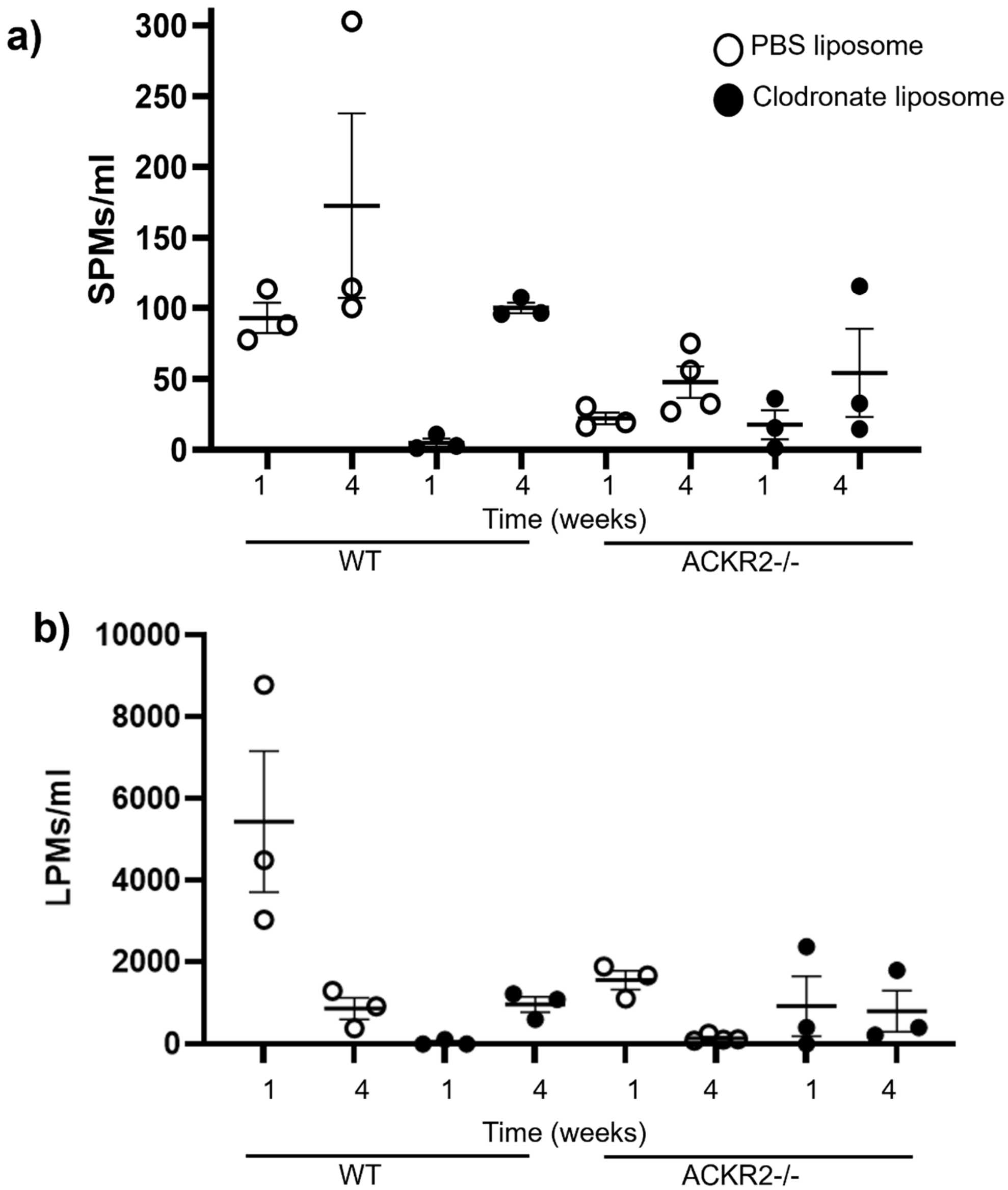

Supplementary Figure 7: Clodronate liposome depletion of SPMs and LPMs. Flow cytometry was carried out to determine the number of **a)** SPMs and **b)** LPMs in the peritoneal cavity 1 and 4 weeks after i.p. injection of 150 µl PBS (white circles) or clodronate (black circles) liposomes into WT and ACKR2-/- mice. *n*=3 per group, except PBS liposome injected ACKR2-/- mice after 4 weeks, *n*=4.

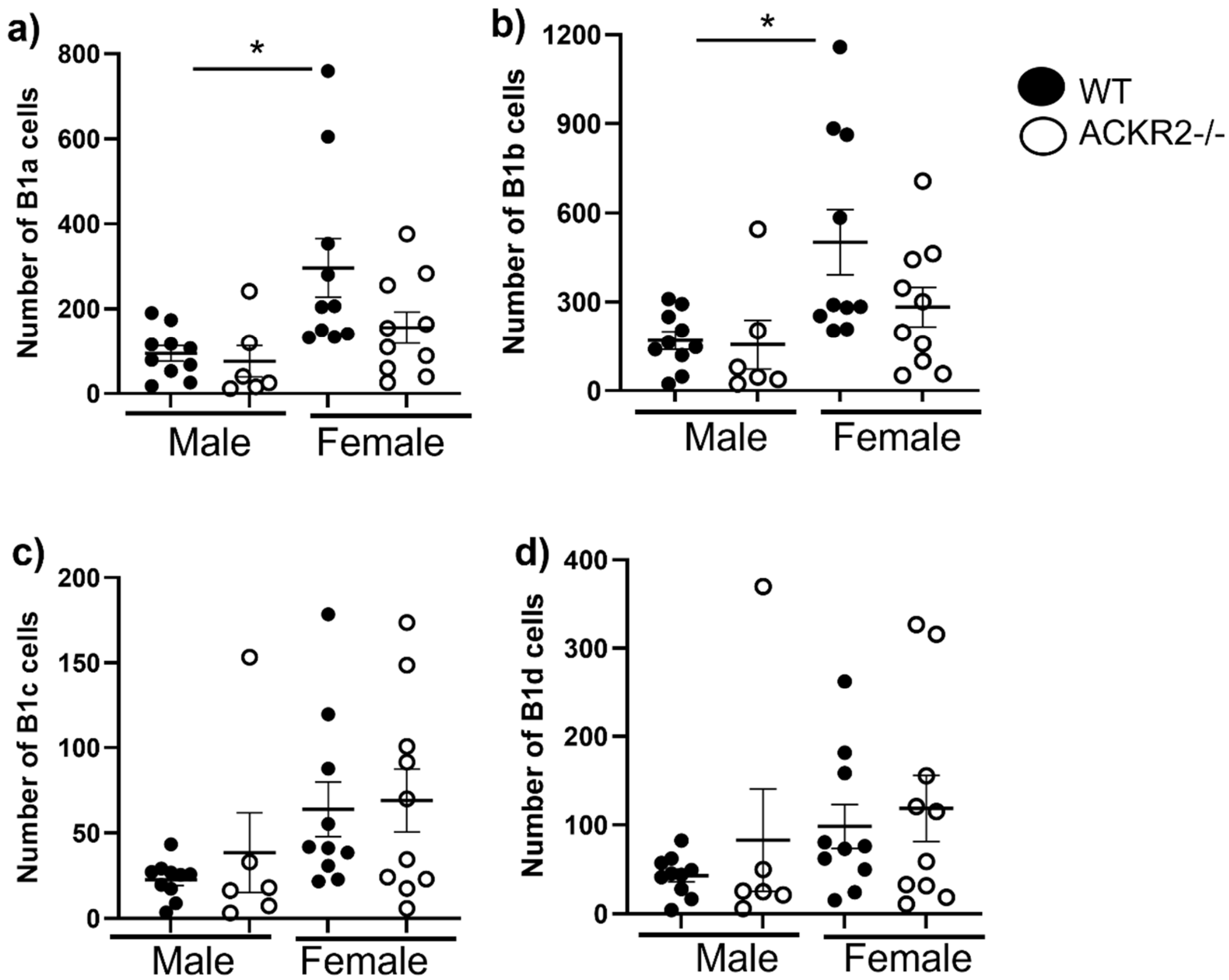

Supplementary Figure 8: B1 cell numbers are higher in male than female WT mice. Flow cytometric analysis was carried out to determine the number of B1 cells., **a)** B1a, Kruskal Wallis test with Dunn's correction, p = 0.019 **b)** B1b, Kruskal Wallis test with Dunn's correction p = 0.0462, **c)** B1c and **d)** B1d cells in the peritoneum of male and female, WT and ACKR2-/- mice. WT male and female n=10, ACKR2-/- male, n=6 and female, n=10). Significantly different results are indicated (*, $p < 0.05$). Error bars represent SEM.

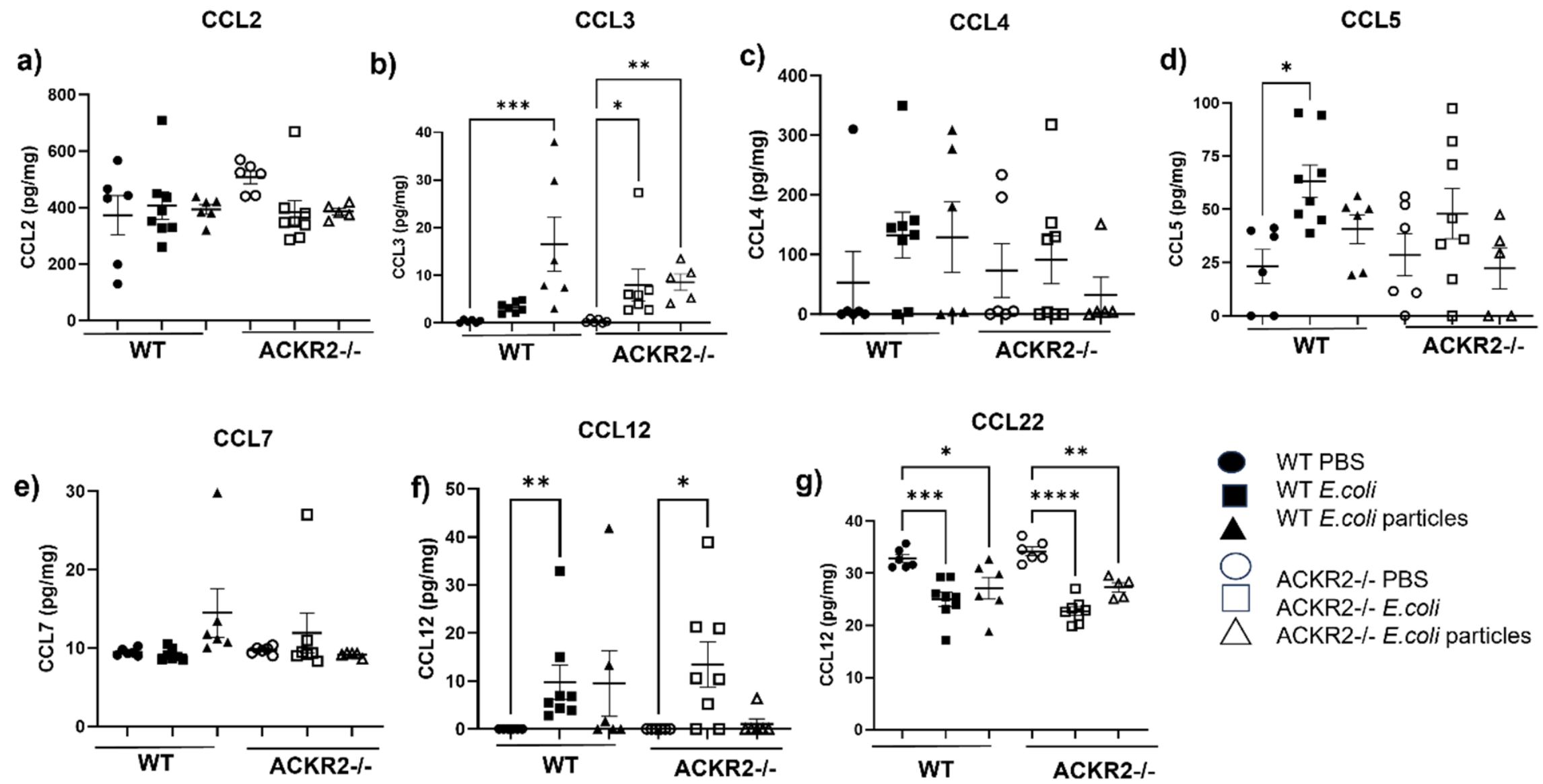

Supplementary Figure 9: ACKR2 does not scavenge ligands in the peritoneal cavity during infection and inflammation. Protein levels were determined by Luminex, after i.p. challenge of WT (denoted by black shapes) and ACKR2-/- (white shapes) mice, with either PBS (circles), 5 × 10^6 CFU *E. coli* strain CFT073 (squares), or 200 µg *E.coli* particles (triangles) for 18 h (PBS, *n*=6 per group, WT *E.coli*, *n* = 8, WT *E.coli* particles, *n*= 6, ACKR2-/- *E.coli*, *n* = 8, ACKR2-/- *E.coli* particles, *n*= 5). Concentrations of the ACKR2 ligands were determined: **a)** CCL2, **b)** CCL3, Kruskal Wallis test with Dunn's correction, WT PBS vs *E.coli* particles, *p* = 0.0004, ACKR2-/- PBS vs *E.coli*, *p* = 0.0167, ACKR2-/- PBS vs *E.coli*, *p* = 0.0031. **c)** CCL4, **d)** CCL5, Kruskal Wallis test with Dunn's correction, WT PBS vs *E.coli*, *p* = 0.0209. **e)** CCL7, **f)** CCL12, Kruskal Wallis test with Dunn's correction, WT PBS vs *E.coli*, *p* = 0.0067, ACKR2-/- PBS vs *E.coli*, *p* = 0.0137 and **g)** CCL22, ordinary one-way ANOVA with Bonferroni correction, WT PBS vs *E.coli*, *p* = 0.0002, WT PBS vs *E.coli* particles, *p* = 0.0146, ACKR2-/- PBS vs *E.coli*, *p* = < 0.0001, ACKR2-/- PBS vs *E.coli* particles, *p* = 0.0037. Significantly different results are indicated (*, *p* ≤ 0.05, **, *p ≤ 0.01*, ***, *p* ≤ 0.001, ****, *p* ≤ 0.0001). Error bars represent SEM.

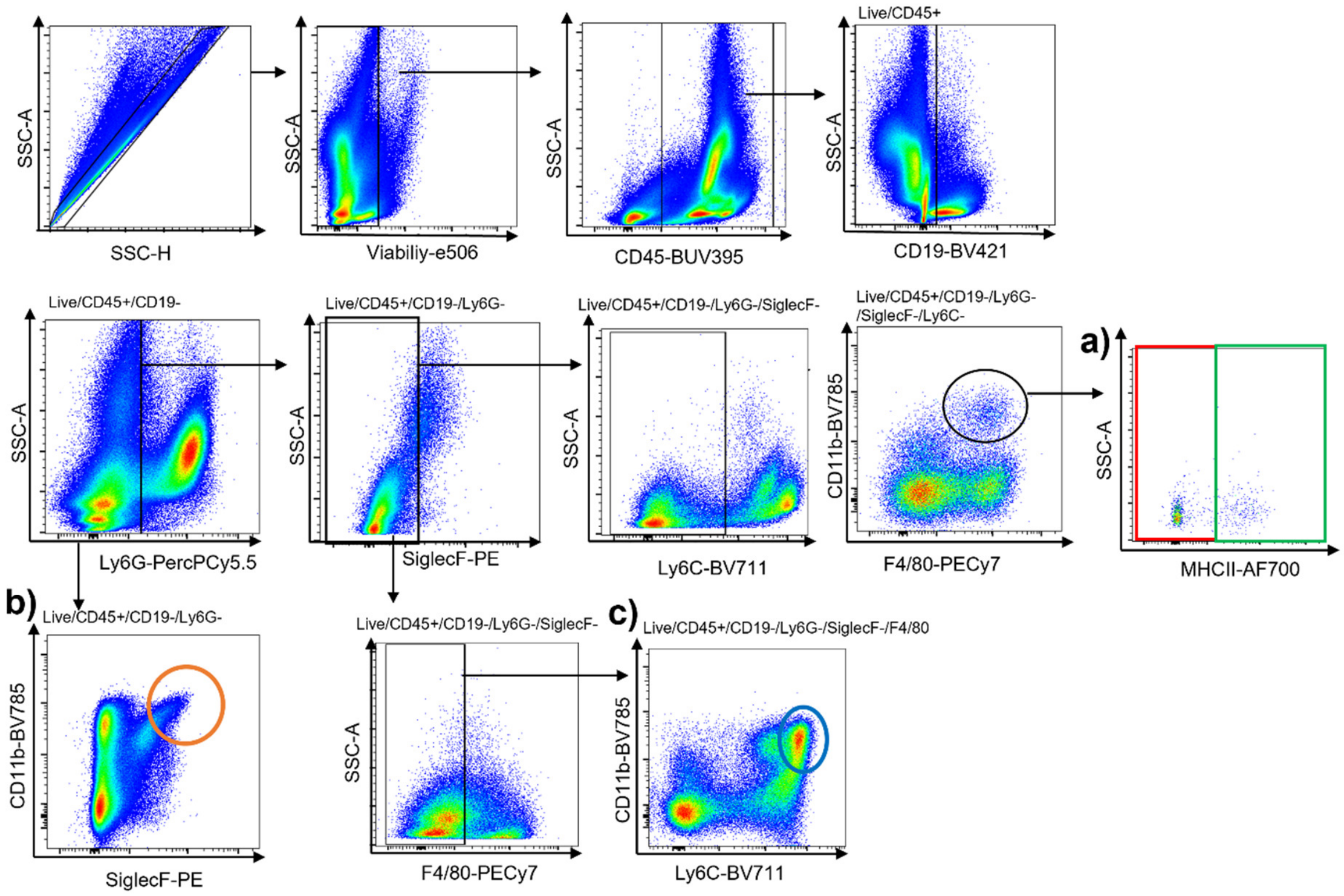

Supplementary Figure 10: Gating strategy to define cell types in the bone marrow. Flow cytometry was carried out to identify cell types in the bone marrow. Single cells were gated, dead cells were excluded, and CD45+, CD19-, and Ly6G- cells were gated. **a)** CD11b+ F4/80+ macrophages were further defined as MHCII negative (boxed in red) and MHCII positive (green). **b)** Eosinophils were gated as CD11b+ and SiglecF+ (orange). **c)** Monocytes were defined as Ly6G-, SiglecF-, F4/80-, CD11b+, Ly6C+ (blue).

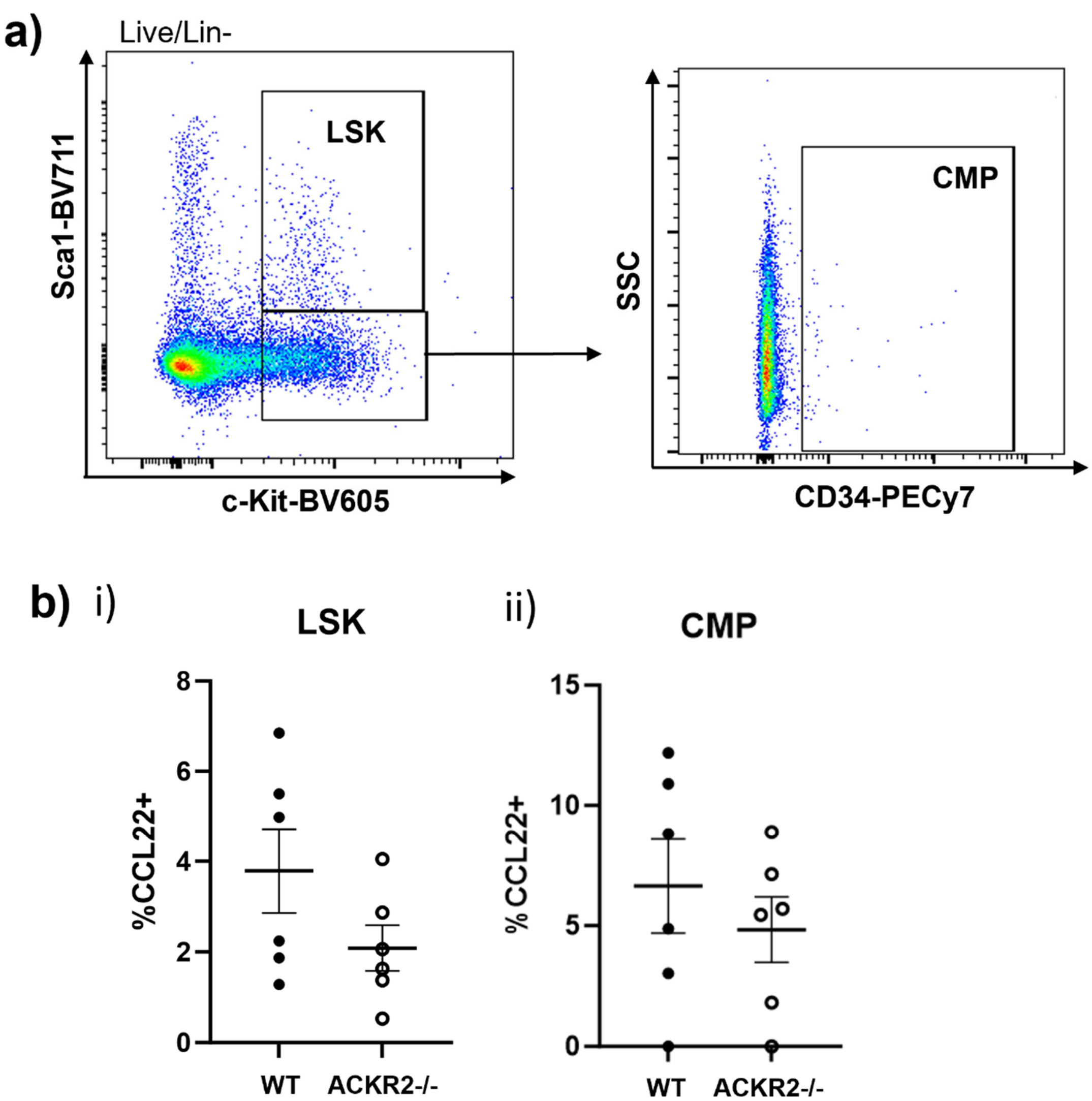

Supplementary Figure 11: ACKR2 is not expressed by haematopoietic precursors in the bone marrow. a) Flow cytometry gating to define LSK (Lin-Sca1+ c-Kit+) cells, and common myeloid progenitors (CMP, Lin-/Sca1−/c-Kit+/CD34+) within the bone marrow. **b)** ACKR2 expression was determined by comparing CCL22-AF647 uptake by **i)** LSK and **ii)** CMP from WT (black circles) and ACKR2-/- (white circles) mice, *n*=6 per group.

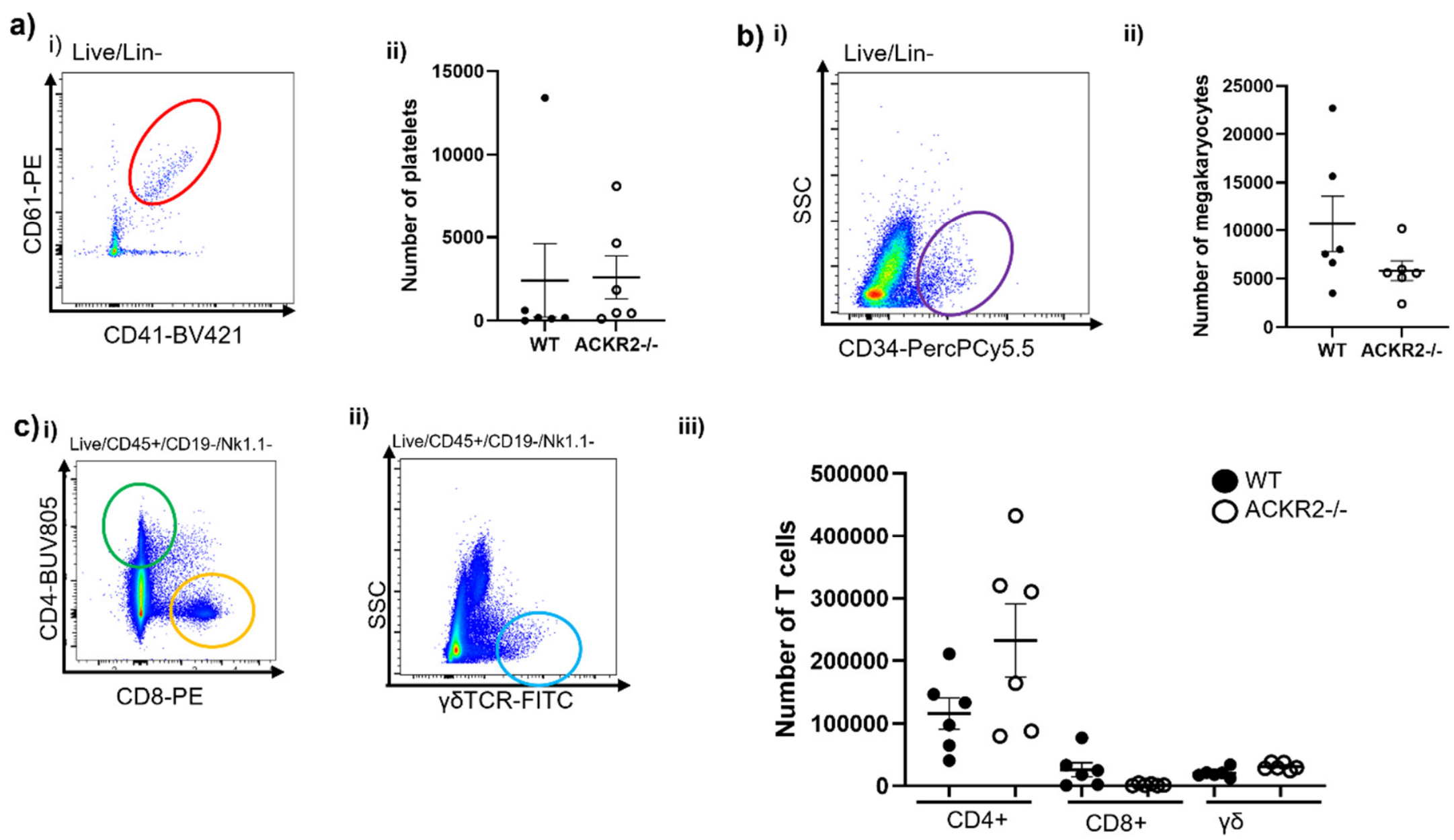

Supplementary Figure 12: T cell, megakaryocyte and platelet numbers are unaffected by ACKR2 deficiency. a) i) Flow cytometry gating strategy to define the **ii)** number of Lin-, CD41 and CD61 positive platelets (circled in red) in blood. **b) i)** gating to define the **ii)** number of Lin-, CD34 positive megakaryocytes (circled in purple) in bone marrow. **c) i)** CD4+ (circled in green), CD8+ (circled in yellow) and **ii)** γδ T cell gating strategy (circled in blue) to define the **iii)** number of T cells in bone marrow between WT (black circles) and ACKR2-/- (white circles) mice. *n*=6, per group.

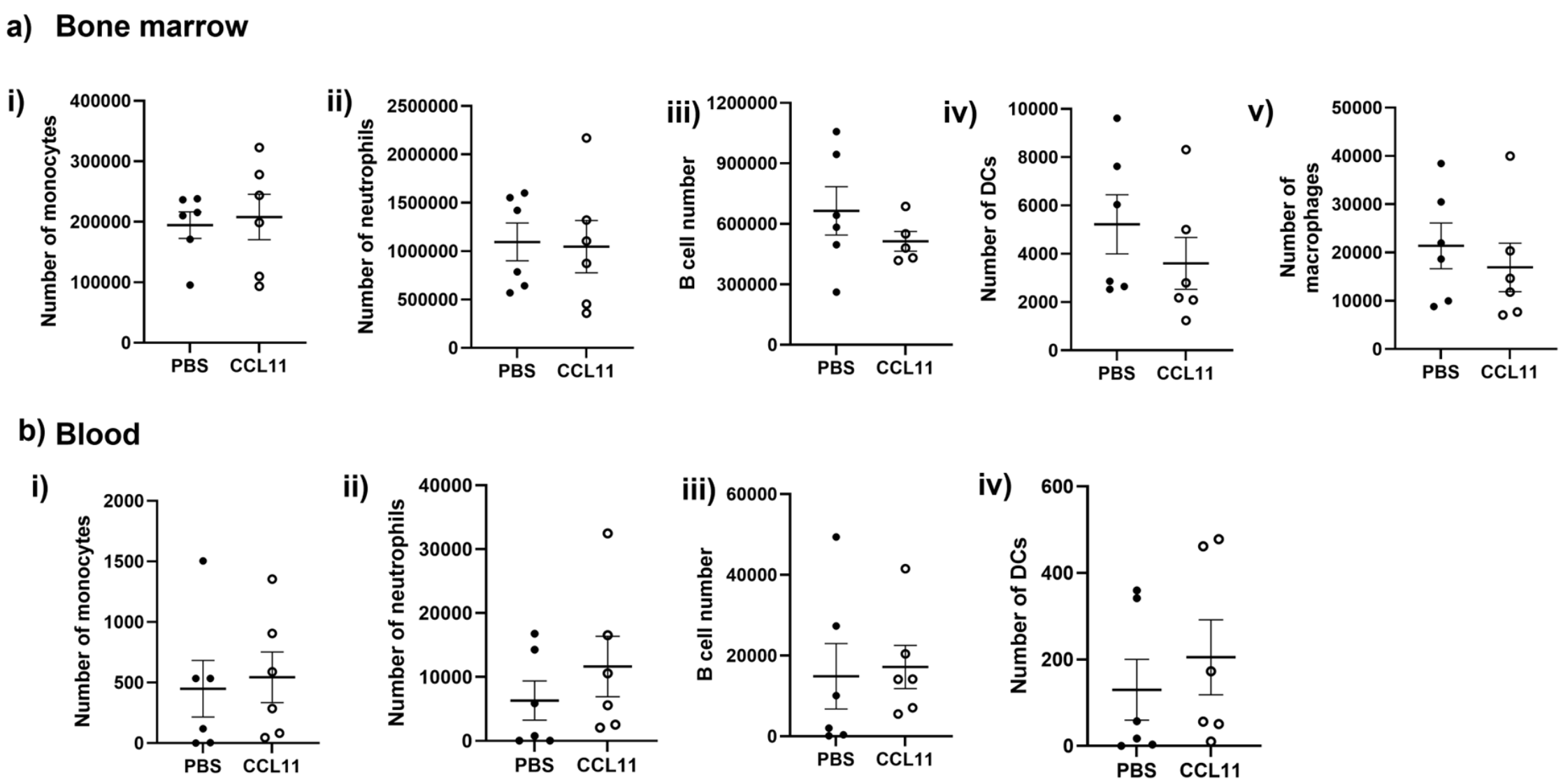

Supplementary Figure 13: Cell numbers after CCL11 injection. Flow cytometry was used to determine the number of **i)** monocytes, **ii)** neutrophils, **iii)** B cells, **iv)** dendritic cells and **v)** macrophages within the **a)** bone marrow and **b)** blood, 1 day after injection of PBS or 1 µg CCL11 (*n*=6, per group).

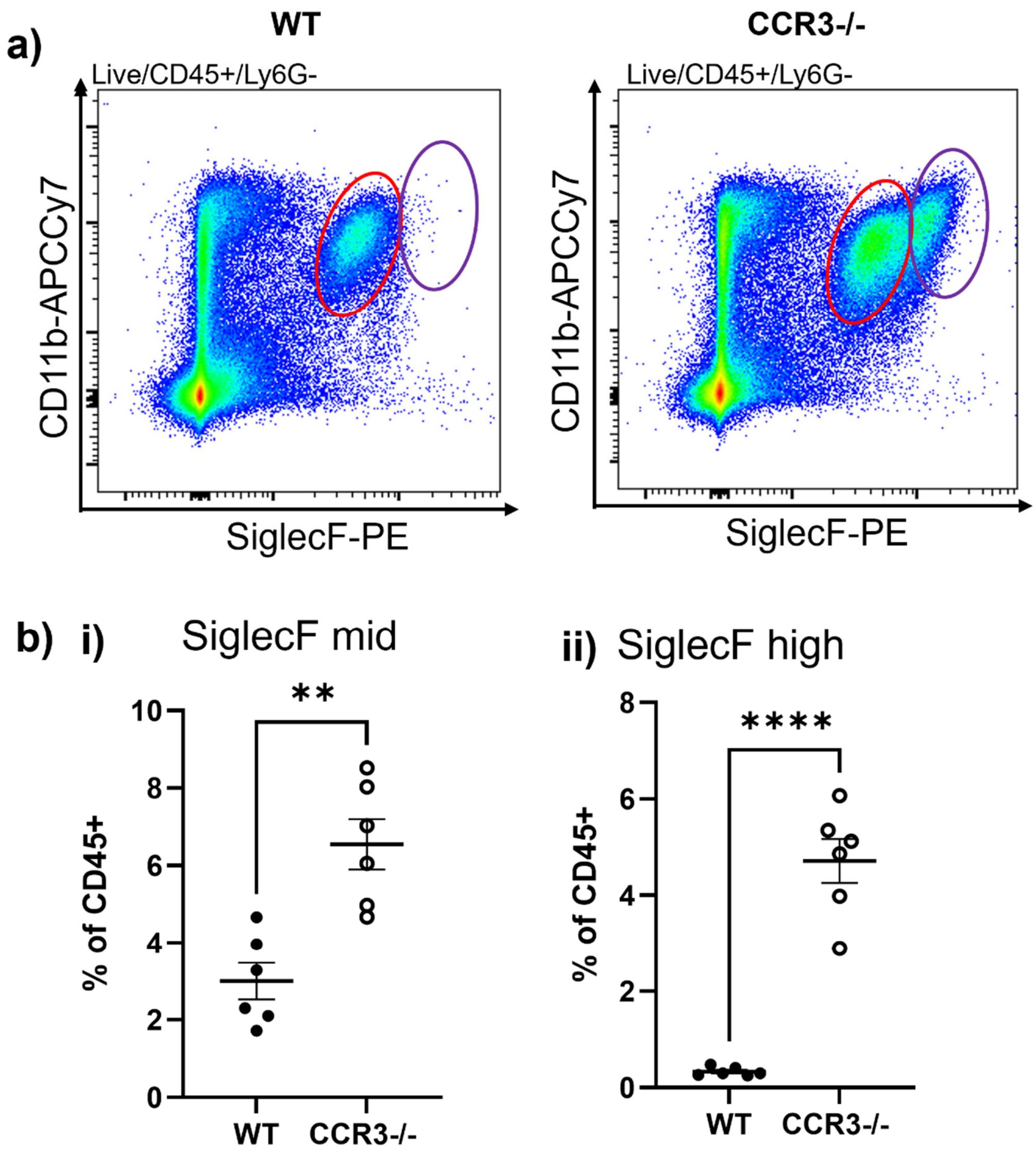

Supplementary Figure 14: Eosinophils accumulate in the bone marrow in the absence of CCR3. a) Flow cytometry was used to determine the **b)** percentage of **i)** SiglecF mid eosinophils and **ii)** SiglecF high eosinophils within the bone marrow of WT and CCR3-/- animals (*n*=6, per group). Unpaired, two-tailed *t* tests, SiglecF mid, $p = 0.0013$ and SiglecF high, $p < 0.0001$. Significantly different results are indicated (*, $p \leq 0.05$, **, $p \leq 0.01$, ***, $p \leq 0.001$, ****, $p \leq 0.0001$). Error bars represent SEM.

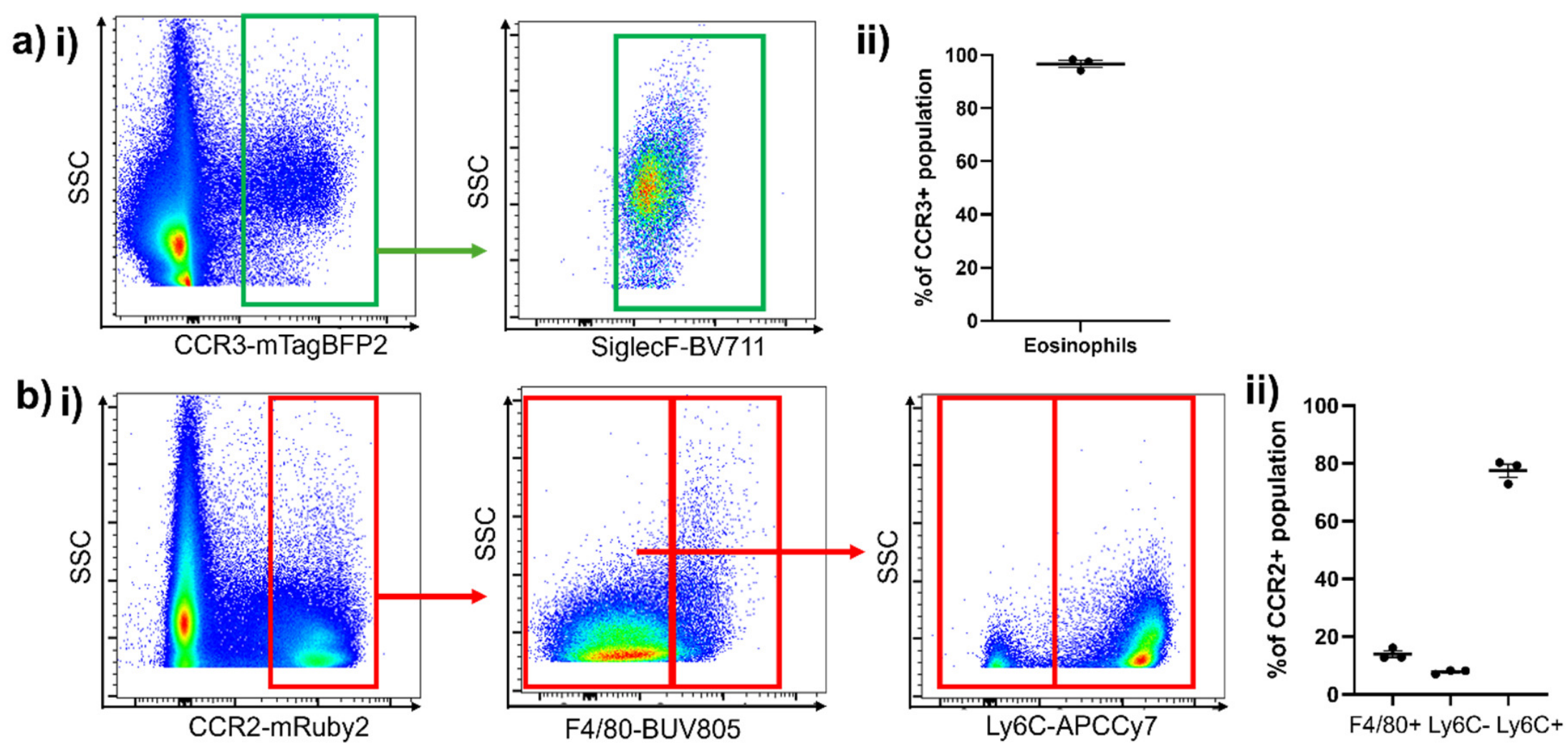

Supplementary Figure 15: CCR2 and CCR3 expression in the bone marrow.

Flow cytometry was used to determine cell types which are **a) i)** CCR3 positive, gated in green, **ii)** Percentage of CCR3+ population which are SiglecF+ eosinophils, and **b) i)** CCR2 positive, gated in red, **ii)** Percentage of CCR2+ population which are F4/80+, Ly6C-, and Ly6C+ cells within the bone marrow of iREP mice (*n*=3).
