# Supplementary material for: ACKR2 aids the regulation of bone marrow eosinophils to promote monocyte differentiation and anti-bacterial immunity
Source: Nat Commun. 2026 Jul 22;17:8954. doi: 10.1038/s41467-026-75964-z (PMC13503710; doi:10.1038/s41467-026-75964-z)
Supplement: Supplementary file 2 [file 41467_2026_75964_MOESM2_ESM.pdf]

nature portfolio

Corresponding author(s): Gillian J Wilson

Last updated by author(s): Jun 23, 2026

Reporting Summary

Nature Portfolio wishes to improve the reproducibility of the work that we publish. This form provides structure for consistency and transparency in reporting. For further information on Nature Portfolio policies, see our Editorial Policies and the Editorial Policy Checklist.

Statistics

For all statistical analyses, confirm that the following items are present in the figure legend, table legend, main text, or Methods section.

n/a	Confirmed	
☐	☒	The exact sample size (*n*) for each experimental group/condition, given as a discrete number and unit of measurement
☐	☒	A statement on whether measurements were taken from distinct samples or whether the same sample was measured repeatedly
☐	☒	The statistical test(s) used AND whether they are one- or two-sided<br>*Only common tests should be described solely by name; describe more complex techniques in the Methods section.*
☒	☐	A description of all covariates tested
☐	☒	A description of any assumptions or corrections, such as tests of normality and adjustment for multiple comparisons
☐	☒	A full description of the statistical parameters including central tendency (e.g. means) or other basic estimates (e.g. regression coefficient) AND variation (e.g. standard deviation) or associated estimates of uncertainty (e.g. confidence intervals)
☐	☒	For null hypothesis testing, the test statistic (e.g. *F*, *t*, *r*) with confidence intervals, effect sizes, degrees of freedom and *P* value noted<br>*Give P values as exact values whenever suitable.*
☒	☐	For Bayesian analysis, information on the choice of priors and Markov chain Monte Carlo settings
☒	☐	For hierarchical and complex designs, identification of the appropriate level for tests and full reporting of outcomes
☒	☐	Estimates of effect sizes (e.g. Cohen's *d*, Pearson's *r*), indicating how they were calculated

Our web collection on statistics for biologists contains articles on many of the points above.

Software and code

Policy information about availability of computer code

Data collection	Flow cytometry data was collected and cells were sorted using BD FACSDIVA software (BD Biosciences).<br>qRT-PCR data collected via QuantStudio v1.3.<br>Magnetic Luminex Multiplex assay (R&D Systems) data was collected using a Bio-Rad Luminex-200 machine.<br>Images were taken using a Zeiss Axioimager M2.
Data analysis	Data was analysed using FlowJo V10 and GraphPad Prism version 10.6.0.

For manuscripts utilizing custom algorithms or software that are central to the research but not yet described in published literature, software must be made available to editors and reviewers. We strongly encourage code deposition in a community repository (e.g. GitHub). See the Nature Portfolio guidelines for submitting code & software for further information.

Data

Policy information about availability of data

All manuscripts must include a data availability statement. This statement should provide the following information, where applicable:

- Accession codes, unique identifiers, or web links for publicly available datasets
- A description of any restrictions on data availability
- For clinical datasets or third party data, please ensure that the statement adheres to our policy

All data supporting the findings of this study are available within the paper and the Supplementary Information. All source data and accession numbers are available. RNA sequencing data is available on Genbank, GEO accession number GSE334547.

Research involving human participants, their data, or biological material

Policy information about studies with human participants or human data. See also policy information about sex, gender (identity/presentation), and sexual orientation and race, ethnicity and racism.

Reporting on sex and gender	N/A
Reporting on race, ethnicity, or other socially relevant groupings	N/A
Population characteristics	N/A
Recruitment	N/A
Ethics oversight	N/A

Note that full information on the approval of the study protocol must also be provided in the manuscript.

Field-specific reporting

Please select the one below that is the best fit for your research. If you are not sure, read the appropriate sections before making your selection.

☒ Life sciences ☐ Behavioural & social sciences ☐ Ecological, evolutionary & environmental sciences

For a reference copy of the document with all sections, see nature.com/documents/nr-reporting-summary-flat.pdf

Life sciences study design

All studies must disclose on these points even when the disclosure is negative.

Sample size	Required sample sizes were not statistically predetermined but were based on many years of previous similar studies.
Data exclusions	For peritoneal lavage analysis, data were excluded where excessive blood contamination was recorded (Fig1a, Fig 2c) or lavage was unable to be performed due to a tear in the cavity wall. Dependent on context, Grubbs test was used to help determine whether a data point was an outlier.
Replication	Key experiments were carried out at least twice. Replication attempts were successful.
Randomization	Samples were not formally randomized. Mice were housed in the same room of the animal facility. For each experiment mice were allocated to age- and sex-matched groups. ACKR2 flox x LysM cre mice were bred as litter-mates to mitigate cage specific effects.
Blinding	Blinding was carried out for counts of image analysis. Researchers were not blinded during animal experiments due to monitoring requirements and for cage identification when collecting animal tissues.

Reporting for specific materials, systems and methods

We require information from authors about some types of materials, experimental systems and methods used in many studies. Here, indicate whether each material, system or method listed is relevant to your study. If you are not sure if a list item applies to your research, read the appropriate section before selecting a response.

Materials & experimental systems

n/a	Involved in the study
☐	☒ Antibodies
☒	☐ Eukaryotic cell lines
☒	☐ Palaeontology and archaeology
☐	☒ Animals and other organisms
☒	☐ Clinical data
☒	☐ Dual use research of concern
☒	☐ Plants

Methods

n/a	Involved in the study
☒	☐ ChIP-seq
☐	☒ Flow cytometry
☒	☐ MRI-based neuroimaging

Antibodies

Antibodies used: Anti-mouse antibodies: CD45 (30-F11), CD11b (M1/70), F4/80 (BM8), Siglec F (S17007L), Ly6C (HK1·4), CD11c (N418), MHCII (M5/114·15·2), CD206 (C068C2), CD5 (53-7.3), CD23 (B3B4), CD45.1 (A20), CD45.2 (104), CD80 (2D10), Sca1 (D7), c-kit (2B8), CD34 (MEC14.7), TGF-β1 (TW7-16B4), CD226 (TX42.1), MerTK (CB10C42), NK1.1 (S17016D),TCR γ/δ (GL3), CD41 (MWReg30) and CD61 (2C9.G2) were obtained from Biolegend. Ly6G (1A8), CD8 (53-6.7), CD4 (GK1.5), CD19 (1D3) were obtained from BD Biosciences. Lyve-1- Alexa Fluor 488 (223322) was obtained from R&D Systems, and ALOX15 Alexa Fluor 488 (AA 581-662) from Antibodies online. Antibodies were used at a dilution of 1 in 200, with the exception of NK1.1 (S17016D), which was used at 1 in 100, in FACS buffer. For the depletion experiment, 20 μg anti-mouse Siglec-F (238047) or isotype control (rat IgG2a, 54447) (Biotechne) was injected i.p every 2 days, 3 times.

Validation: All antibodies were obtained from commercial companies and have been validated and used at 1 in 200 dilution (except for NK1.1, 1 in 100): Biolegend, BD Biosciences, R&D Systems, and Antibodies Online.

Animals and other research organisms

Policy information about studies involving animals; ARRIVE guidelines recommended for reporting animal research, and Sex and Gender in Research

Laboratory animals: Wild-type (WT) C57BL/6J mice were purchased from Charles River Laboratories. CD45.1, ACKR2-/- , iREP and CCR3-/- mice were bred in the specific pathogen-free facility of the University of Glasgow Wolfson Research Facility (WRF). Males and females were used and age was 8-10 weeks unless stated otherwise.

Wild animals: N/A

Reporting on sex: Males and female animals were used. Numbers and sex have been described.

Field-collected samples: N/A

Ethics oversight: Animal experiments were carried out in accordance with UK Home Office regulations (Project Licenses PP6655603 and PP1443954). All experiments conformed to animal care and welfare protocols, approved by the Animal Welfare Ethical Review Body at the University of Glasgow. All mice were housed in individually ventilated cages (IVCs) within a controlled animal facility. Animals were maintained on a 12h light/dark cycle, with lights on at 07.00 and off at 19.00. Environmental conditions were maintained at a temperature of 19–23C and a relative humidity of 55% (±10%). Animals were group-housed with appropriate environmental enrichment.

Note that full information on the approval of the study protocol must also be provided in the manuscript.

Plants

Seed stocks: N/A

Novel plant genotypes: N/A

Authentication: N/A

Flow Cytometry

Plots

Confirm that:

☒ The axis labels state the marker and fluorochrome used (e.g. CD4-FITC).

☒ The axis scales are clearly visible. Include numbers along axes only for bottom left plot of group (a 'group' is an analysis of identical markers).

☒ All plots are contour plots with outliers or pseudocolor plots.

☒ A numerical value for number of cells or percentage (with statistics) is provided.

Methodology

Sample preparation	Source of cells and tissue processing has been described in the manuscript.<br><br>Dead cells were excluded using Fixable Viability Dye eFluor 506 (Thermo Fisher Scientific). Cell suspensions were blocked with TruStain FcX (Biolegend) at 1:200. Antibodies were used at a dilution of 1 in 200, with the exception of NK1.1 (S17016D), which was used at 1 in 100, in FACS buffer (PBS, 1% foetal bovine serum (FBS), and 2 mM EDTA (Thermo Fisher Scientific)). Cell suspensions were washed with FACS buffer and fixed with 2% PFA. Intracellular staining was carried out using the Cyto Fast Fix Perm buffer set (Biolegend), as described in the manufacturer's instructions. Flow cytometry was performed using a Fortessa or Celesta (BD Biosciences) and analysed using FlowJo V10. FACS sorting was carried out using a FACSaria III (BD Biosciences).
Instrument	Flow cytometry was performed using a Fortessa or Celesta (BD Biosciences). FACS sorting was carried out using a FACSaria III (BD Biosciences).
Software	Data was analysed using FlowJo V10 and GraphPad Prism version 10.6.0.
Cell population abundance	Cell abundance has been described throughout the manuscript.
Gating strategy	Gating strategies have been included in Supplementary Figures 1, 3 and 10.

☒ Tick this box to confirm that a figure exemplifying the gating strategy is provided in the Supplementary Information.
