# Supplementary material for: ACKR2 aids the regulation of bone marrow eosinophils to promote monocyte differentiation and anti-bacterial immunity
Source: Nat Commun. 2026 Jul 22;17:8954. doi: 10.1038/s41467-026-75964-z (PMC13503710; doi:10.1038/s41467-026-75964-z)
Supplement: Supplementary file 3 [file 41467_2026_75964_MOESM3_ESM.pdf]

nature portfolio

# Peer Review File

# ACKR2 aids the regulation of bone marrow eosinophils to promote monocyte differentiation and anti-bacterial immunity

Corresponding Author: Dr Gillian Wilson

**This file contains all reviewer reports in order by version, followed by all author rebuttals in order by version.**

Version 0:

Reviewer comments:

Reviewer #1

(Remarks to the Author)
This paper delineates the effect of the atypical chemokine receptor Ackr2 on macrophage differentiation. The authors find a very narrow effect on a subpopulation of small peritoneal macrophages (SPMs). They show that an E. coli challenge to the peritoneal cavity is not normally controlled possibly due to the SPM deficit. They show that ACKR2 deficiency is unlikely to act locally in the PC to cause SPM deficiency but rather remotely in the bone marrow where they document small changes in chemokine and eosinophil levels. They provide some data supporting eosinophil modulation of SPM differentiation ex vivo. Overall, the results are creative and novel and integrate events at the molecular, cellular and immunologic/clinical levels. The authors show a major effect of ACKR2 deficiency on monocyte differentiation at the transcriptome level, so that the effect limited to SPM that they found is somewhat surprising. Possibly additional investigations would reveal other important abnoralities in myeloid cells. The paper would be strengthened by direct tests of eosinophil or CCL11 modulation of SPM differentiation and distribution in vivo.

1. Figure 1: What do SPM in peritoneal cavity and MHCII neg interstitial macrophages in lung share that other macrophage populations tested do not share that might explain their shared dependence on ACKR2 function to attain normal levels in these organs?
2. Line 152-154: the increase at 18 hours could just reflect the increased number of both SPM and LPM in ko vs wt as shown in Figure 2c iii and iv.
3. Figure 3f: the results in vitro in part f are not consistent with the results in vivo in part e, neither at the level of % ECP+ cells which differ by more than an order of magnitude, nor by the ACKR2 dependence which is positive in f and negative in e. I don't see how the in vitro results explain the in vivo findings.
4. Figure 3: if ACKR2 functions as a chemokine scavenger in vivo, the levels of ACKR2 ligands should differ between wt and ko mice in vivo. That they don't in the peritoneal cavity may mean that ACKR2 expression is so low as to be trivial in that compartment. The in vivo results with the CCL22 uptake assay suggest that may be the case since the receptor is not expressed on macrophages and is only 1-20% of B1 cell subtypes. B1a and d cells have the most by %. What proportion of PC B cells are B1a and d?
5. Figure 4: why do the wt monocytes appear to generate more SPMs in the ko background? What are the results for both adoptive transfers for LBMs?
6. Did ACKR2 ko affect monocyte levels in blood and bone marrow? What were the levels of CCR2 on wt vs ko monocytes? The marked difference in CXCR4 between wt and ko might anticipate a major difference in monocyte distribution that would affect all monocyte-derived cell populations, not the highly selective macrophage abnormality that was observed.
7. Figure 6aiii: this evidence for ACKR2 expression on BM macrophages and B1b cells is not convincing. Results are noisy for macrophages, and the small difference for ko vs wt among cells with a very low signal in the assay is of uncertain significance. Do these cells express ACKR2 RNA?
8. Figure 6b: the authors focus on CCL11, but there are also other differences that are not mentioned including CCL5, which is also an eosinophil chemoattractant acting through the same receptor, and CXCL12, which is an important regulator of distribution for most types of leukocytes. The net result of all these changes on any one cell type are therefore complex to define and not defined in this study. Moreover, all of the differences are quite small in magnitude and not all in the same direction so of uncertain significane. Injection of 1 microgram of CCL11 is a pharmacologic challenge that may not inform the impact of such small changes in CCL11 in the bone marrow.
9. Figure 7: A direct test of the physiologic relevance of CCL11 to SPM differentiation and localization in PC would be to evaluate SPM in peritoneal cavity in CCL11 and/or CCR3 ko mice. A direct in vivo test of the importance of eosinophils in

SPM differentiation would be to evaluate SPM in peritoneal cavity in eosinophil-deficient mice such as the PHIL and dbGATA lines, or in mice depleted of eosinophils with anti-CCR3.
10. The results in Figures 6 and 7 show indirect and in vitro results with small effect sizes that are of uncertain causative significance. In panel b, there is no unequivocal effect of eosinophils on CD206 MFI. In panel c, the experiment is underpowered with n=4. The eosinophil wt and ko results are both lower than the control and ko results are greater than wt as for the control. Hard to conclude anything solid from this. In panel d, the effect size is very small. The purity of the population for eosinophils should be stated in the figure legend. Can any other cell type increase these markers, e.g. neutrophils?

Reviewer #2

(Remarks to the Author)
I co-reviewed this manuscript with one of the reviewers who provided the listed reports. This is part of the Nature Communications initiative to facilitate training in peer review and to provide appropriate recognition for Early Career Researchers who co-review manuscripts.

Reviewer #3

(Remarks to the Author)
In this manuscript by Wilson et al. the authors describe data from mice deficient in the atypical chemokine receptor, ACKR2, that suggests its role in regulation monocyte function indirectly via eosinophil location/mobilization. First, they find that mice lacking this receptor have reduced numbers of monocyte-derived macrophages in the lung and peritoneal cavity, while observing normal resident macrophages, suggesting an impairment in recruited macrophages. They go on to show that scavenging of the chemokine CCL11 is blocked in ACKR2-/0 The authors provide a compelling data set in support of their overall conclusions, although there are several points that the authors are suggested to consider and address.

1. Supplementary Figure 3 is intended to support the idea that the small peritoneal macrophages (SPMs) are reduced and that their replacement is poor (upon clodronate depletion) in the ACKR2 KOs relative to wildtype controls. However, there are no statistical analyses on these data to support this conclusion and a fair amount of spread in the data. Notably, whereas depletion via clodronate is apparent in the wildtype mice it appears that clodronate-mediated depletion is not effective in the ACKR2 KO mice, perhaps because their ability to take up and liberate/digest the liposomes and release clodronate (to cause cell death) is impaired? Nonetheless, the authors conclusion from these data are not well supported.

2. The authors claim that there were no changes in macrophages in various tissues (line 100 and Supp. Fig. 5), however there are strong trends for reduced macrophages in each tissue. If the macrophages are not subdivided into MHC+/- and total macrophages were examined, are there reductions? Similarly, there are some differences in monocytes that the are also not discussed (ie, monocytes are increased in the blood).

3. Adoptive transfer experiments in Figure 4a-ii are underpowered. There are two distinct populations in the ACKR2-/- group and with just 4 mice in this group/experiment, the conclusion that there's no difference is not convincing.

4. Figure 5 is confusing and there are some issues with labeling that made it hard to follow and interpret. (1) The authors show wt followed by ACKR2-/- in most figures and panels but then reverse this in panel (e) showing KO and then WT (unless these labels are wrong?). (2) In panel (e) the graphs depicting SPMs and LPMs (i and iv) should be clearly labeled. (3) In panels within (e) the circles for PBS and E.coli are reversed. It's also unclear what is even being measured in the PBS group? Why would anything come up in this group as positive? It would be an important control, for staining, but likely is not relevant or needed in the graphs. (4) The data in panel (f) do not match the text and labeling provided (WT are labeled as open triangle and ACKR2-/- labeled with closed triangle) and text describes the data opposite to what is shown. Again, there is no conclusion from these data.

5. The authors conclude that CCL11 is responsible for regulating eosinophil retention and mobilization from the bone marrow. They show that increased CCL11 in the bone marrow correlates with reduce eosinophils in this tissue and recombinant CCL11 can cause increased blood eosinophils (Fig. 6. d). However, the percentage by flow is really quite low- can the authors also perform CBCs to show an absolute increase in eosinophils? These data are not convincing and do not show the impact of CCL11 on other cell types. Panel (e) in Figure 6 should be more clearly labeled as bone marrow. It's unclear why the PBS ACKR2-/- group (only 2 mice) is so different from data shown in c.) i).

6. Data in Figure 7 are quite interesting, but additional methods for identifying monocytes and eosinophils, besides chemokine receptor expression would strengthen the conclusion that the monocytes and eosinophils are interacting and in close proximity. The monocytes appear to be surrounding eosinophils- is this expected? Using this mouse and manipulating CCL11 levels would be quite interesting, to determine how CCL11 impacts these contacts. It could help corroborate the data shown in Figure 6.

Minor:

1. Figure legend titles should more accurately reflect the key findings. For example, in Figure 1- the authors should state

which macrophage population is decreased in ACKR2-deficient mice as the LPMs are unchanged.
2. Concluding statements are sometimes missing from the results and the authors main point is not always clear. Especially as some data are not terribly clear or convincing. For example, regarding the first results section, the authors look at many different tissues to examine monocytes and macrophages. In the statement on 101, it's not clear why the authors write "Importantly"- what is important about the observation? That monocyte recruitment is the same? It just needs to be explained more clearly.
3. The authors do not comment on whether B1 cells are similar in number between the wildtype and ACKR2 KOs. It seems important and relevant to also show ACKR2 expression on macrophages.
4. In Supplemental Figure 9 the authors define common myeloid progenitors (CMPs) as being gated through the LSK population, however, CMPs are defined as being Sca-1-negative, so these data are incorrect.

Version 1:

Reviewer comments:

Reviewer #1

(Remarks to the Author)
This manuscript requires a lot of clarifications as detailed below. Also, I'm not satisfied with the authors response to point 9 in my original review requesting in vivo evidence that eosinophils affect monocyte to macrophage differentiation (and E. coli burden in the peritoneal cavity). Overall, the associations and in vitro studies performed are of interest but don't provide sufficient in vivo evidence for the main conclusions captured in the title of the paper.

1. Figure 2: Figure 2B-D, need to indicate the timepoint of analysis in the figure legend. The SPM and LPM dynamics are associations and do not and cannot causally explain the increased bacterial burden in ko mice. The authors describe this appropriately conservatively. However, their description would be improved by drawing attention to the following: there is a 10X increased E. coli burden in ko vs wt PC, yet no difference in burden in ko and wt PCW where the burden is 100-1000-fold greater than PC despite the fact that SPM content in PCW is lower in ko than wt at baseline and SPM exceeds LPM in PCW. Perhaps this is why there is no weight change difference for wt vs ko, i.e. the increase in burden in PC contributes little to the overall burden in the peritoneal compartment (wall plus cavity).
2. In panels 2ciii and 2di for the peritoneal cavity and cavity wall PBS controls there is no difference in SPM number between WT and KO in either case. This does not agree with the results in Figure 1aii and 1bii. The absolute numbers of SPM in the PBS control condition for cavity wall in 2di are on average 3-fold lower than shown in Figure 1bii and the absolute numbers for SPM/PBS in the peritoneal cavity are ~10-fold higher than for baseline conditions shown in Figure 1. What would be the mechanism to explain such a large and differential PBS effect on SPM content that effaces the difference between wt and ko found at baseline in Figure 1?
3. Figure 2E: how is it possible for there to be ~80% ECP+ SPM in both ko and wt at 1 hour (panel 2ei) AND there to be ~200 and 100 SPM/ml at baseline in wt and ko PC, respectively (Figure 1), YET only 5 and 1 ECP+ SPM in wt and ko PC at this timepoint (panel 2eii)? Does the PC content of SPM decline that quickly, and to a greater extent for ko than wt at 1 hour (in contrast to 18 hours)? Can't tell from the rest of the figure, where the timepoint is apparently 18 hours. Also, need to clarify in the figure legend whether the data are for PC or PCW or both. The numbers don't align for LPM either comparing 2civ and 2evi.
4. Figure 2f: clarify that the cells are from naïve mice (not infected). Also, for 2fiii the timepoint for BMDM is not specified in the text or the figure legend, unlike the other 2f panels. Point out that there is a massive difference in %+ SPM from PC in vitro vs in vivo. Why would that be? Are the cells more efficient in vitro at breaking down PHRODO? It's not clear what you are concluding from these PHRODO studies. If the ACKR2 ko cells have a phagocytic defect, would that not also be relevant in the PCW where there is no difference in bacterial burden between ko and wt?
5. Figure 3d: the symbols are not defined.
6. Figure 4 legend needs more detail. E.g. panel b doesn't indicate what cells were cultured (sorted BM monocytes?); panel bii y-axis indicates the unit but not the parameter (MHCII expression). Panel c doesn't indicate what cell type is treated with IL-4 and lps and for how long and doesn't specify what the symbols refer to (wt and ko but which is which?).
7. Line 268: there is nothing in the Introduction or the Results to this point that would explain the motivation for testing CD80 and CD206 or the significance of the results obtained. This should be clarified here.
8. Figure 6av and 6bi and ii: the open symbols are defined as ACKR2, not ACKR2 ko.
9. Figure 6: panels i and ii are not described or called out in the Results section. The evidence for ACKR2 expression on BM macrophages and B1b cells is not convincing. For B1 cells, there is a very small effect size for decreased % CCL22 uptake positive B1b cells in ko compared to wt shown in panel 6av. And only 1-2% of B1b's are positive in this assay. Moreover, B1a and B1c cells have much higher levels of uptake positive cells than B1b's without any decline in kos and without any data shown for RNA in panel 6avi, raising questions as to whether the B1b signal is really just noise. Likewise, the positive macrophages represent only ~12% of cells and the specific signal is extremely small. Can the authors show MFI data that would more convincingly demonstrate a signal against the noise in these few 'specific' uptake positive cells?
10. Figure 6b: likewise, the CCL11 expression signal in BM is quite small, a mean difference of 1 vs 2 pg/mg in bone marrow with high variance. CCR3 is known to have about 12 chemokine ligands. Can the authors find one whose expression is robustly affected by ACKR2 ko?
11. Figure 6d: what is the effect of injecting CCL11 into ACKR2 ko mice?
12. Figure 6e legend: indicate how many hours after PBS injection the eosinophil levels were assessed. It's hard to imagine that PBS injection would have sustained effects on eosinophil levels in BM only in the ko whereas E. coli infection would not. The authors speculate the changes are due to effects on egress. What are the associated blood eosinophil levels?

13. Line 399-400: this overstates the evidence in the figure. BM eosinophil levels appear to be ACKR2-dependent and CCL11-mobilizable. The precise mechanism for altered eosinophil levels in ACKR2 ko BM is still unclear.
14. Figure 7: while the effect of CCL11 on interacting cells is of interest, of greater interest is how the density of interacting cells is affected by ACKR2 ko. And while the in vitro studies are of interest, of greater interest is whether animals lacking or overconcentrating eosinophils in bone marrow would have abnormal monocyte to macrophage differentiation in vivo, resulting in altered levels of SPM in PC and PCW. For example, how are PC and PCW SPM and LPM levels and E. coli burden affected in CCL11 ko mice, CCR3 ko mice, PHIL mice, dbGATA mice? I don't understand the logic for the response to point 9 in the original review.

Reviewer #3

(Remarks to the Author)
Gillian et al. responded to the reviewer comments by providing new data and modifying some conclusions and statements. Overall, the editorial changes and corrections and additions improved the manuscript, though there are still some issues that should be addressed.
The key findings of this manuscript are that eosinophils and monocytes interact in the bone marrow and that these interactions impact monocytes in such a way that they are functionally and numerically different in specific sites like the peritoneal cavity. The concept is quite interesting, but the biological significance is not clearly articulated.

1. There are some trends that are obvious, and although the changes don't reach significance, the authors conclusions are not convincing. For example, there appear to be changes in the omentum that are dismissed and overlooked. There are also striking sex differences in B1 cells that are not discussed.
2. The authors rightfully focus on chemokines, as the ACKR2 receptor is known for scavenging chemokines, but they ignore the role of other factors that are likely going to be impacted by a change in chemokine availability. The authors do not discuss why CCL5 production would be decreased in the ACKR2-/- mice aside from a role of monocytes which are not likely the main source of CCL5 in the bone marrow. Did the authors examine megakaryocytes and T cells in the bone marrow? Did they assess platelets in circulation?
3. The imaging studies that demonstrate interactions between CCR3+ and CCR2+ cells in the bone marrow are interesting though not entirely convincing. There appear to be interactions in the CCL11 treated mice as well and there also seem to be more CCR2+ cells in these images? Would that suggest that there are more monocytes? They do not show data for this, only eosinophils.
4. Why did the authors not deplete eosinophils and/or use models to investigate how increasing eosinophils would impact monocytes to more directly assess the in vivo role of eosinophils in modulating monocytes in the bone marrow?
5. It is unclear if the receptor is intrinsically required by monocytes for the observed changes. The adoptive transfer experiments were a good approach to try to test this, but monocytes were obtained from wildtype and complete knockout animals. Mixed chimeric mice would have helped to address this question (using both WT and ACKR2-deficient hosts). As ACKR2 is thought to be expressed primarily by non-hematopoietic cells, these studies would have helped to directly address the role of ACKR2 in monocytes themselves. As there is a fundamental change in monocytes in the knockouts that results in impaired differentiation, it seems important to address how ACKR2-deficiency itself impairs differentiation programs. Are there changes in expression of MCSFR on ACKR2-/- monocytes? Are there differences in M-CSF in the knockouts?

Version 2:

Reviewer comments:

Reviewer #1

(Remarks to the Author)
The authors have clarified some points and have added important macrophage specific Ackr2 ko data; however, the mechanistic claims made still exceed the strength of the data supporting them, particularly with regard to in vivo evidence, as follows:

Lines 125-126 indicate that in Figure 2 the authors 'investigated whether reduced macrophage numbers as a result of ACKR2 deficiency had functional consequences during peritoneal infection with E.coli'. However, the data are not convincing. First, more cell types than macrophages have abnormal numbers in the peritoneal cavity of the total kos, including neutrophils and monocytes as shown in figure 2. However, the experiments are not actually designed to determine which of these alterations, if any, account for the increased E. coli burden in the kos. Infections of LyseM cre X floxed Ackr2 mice would at least allow specific statements of the role of macrophage Ackr2 on E. coli control in the model. Second, unlike in naïve animals, baseline macrophage content after PBS injection is not different for either SPM or LPM in either peritoneal cavity or cavity wall in ACKR2 ko vs WT animals. The authors speculate that this is ''due to damage at the injection site, leading to a disappearance reaction in WT mice, again not observed in ACKR2-/- animals''. However, in the same sites putatively damaged by PBS injection there is also no difference in neutrophil or monocyte content, i.e. no convincing evidence of significant damage. A defect in the same macrophage disappearance reaction is invoked to explain why SPM and LPM content is not decreased but instead is increased (i.e. less disappearance) in E. coli-injected ACKR2 ko vs WT animals in the peritoneal cavity, and SPM content is not different in cavity wall of E. coli injected wt vs ko animals. Yet, in the

same animals LPM content is decreased in the cavity wall of E.coli injected kos compared to wts, and this is associated with no difference in E. coli burden in the site. Very confusing and inconsistent, and allows for statements about associations, not causality about the functional consequences of reduced macrophage numbers in this infection model.

Line 350: the claim that ‘ACKR2 scavenges CCL11 to regulate eosinophils in the bone marrow and blood’ is based on associations of very low levels of expression of Ackr2 in macrophages with very small changes in CCL11 and larger changes in eosinophil content in kos, and is not convincing. The levels of CCL11 measured in wt bone marrow are in the femtomolar range, well below the kd for binding to the CCL11 receptor CCR3, and the ko results in only a small increase in that level, still in the femtomolar range. Moreover, there are even smaller increases in higher levels of CCL5, a second important CCR3 ligand. There are no data to define the individual or net causal effect of these associated small and opposed expression changes compared to wt on eosinophil content in BM. Moreover, the Ccl22 scavenging assay has a very high background as defined in ko macrophages relative to the very low levels of 2-3% +ve macrophages and MFI in wt macrophages. It is not clear that this small difference in CCL11 protein expression in wt vs ko bone marrow is from scavenging differences attributable to the small observed differences in Ackr2 wt vs ko macrophages as opposed to some other mechanism such as small changes in CCL11 gene expression, among other possibilities. Nor is it clear, the CCL11 challenge data notwithstanding, that this miniscule difference in endogenous CCL11 content in ko vs wt accounts for the difference in eosinophil content.

Line 478: what is the timepoint for these data? How was a region of interest defined?

Line 437: The claim that ‘Eosinophils promote monocyte to macrophage differentiation’ is not fully supported. First, Figure 7 does not establish whether the macrophage-interacting eosinophils occur at random. At least, what is the percentage of macrophages that form interactions with eosinophils and vice versa? The distribution of red and blue cells in Figure 7b show very few that are adjacent, maybe just random and of unknown significance. Second, CCL11 injection is known to mobilize eosinophils from bone marrow (figure 6), without affecting macrophages, so it is not surprising that the density of apparent macrophage-interacting eosinophils would decrease after CCL11 injection, even if the apparent interactions occurred at random. Third, figure 7c shows that eosinophils per se have no effect on CD206 expression (as a marker of macrophage differentiation) on cocultured BMDM in vitro and only slightly boost the large increase of CD206 induced by recombinant IL-4. Fourth, eosinophil coculture had no effect on ECP phagocytosis by cocultured wt BMDM and only a minor effect on this parameter for cocultured ko BMDM. It isn’t established that BMDM produced this way express Ackr2 or whether Ccl11 or any other Ackr2 or eosinophil activating chemokine would be present in this system to play a role. As a clerical point, the figure shows 8 data points for each condition, whereas the figure legend indicates n=4. On line 671, it isn’t clear what the numerator and denominator are in the 1:25 ratio. This should be indicated in the figure legend as well along with the duration of the coculture before assay. Fifth, in figure 7e, there is a small effect size of eosinophil cocultivation with monocytes with regard to expression of Alox15 and TGFbeta; however the induction is not dependent on monocyte-eosinophil contact. As a clerical point, the figure legend indicates n=7 for the control condition, which is correct for Alox 15 but not TGFbeta. The figure does not specify whether the monocytes are from wt or ko mice or why only one genotype was tested. For all the in vitro experiments, it isn’t clear whether each data point indicates cells from a different mouse or from one mouse, nor whether the data are summary data from multiple experiments or one experiment that is representative of other experiments. Therefore, the data as presented are not clearly reproducible. Given the above, as mentioned previously, the absence of in vivo evidence that eosinophils promote macrophage differentiation remains a significant limitation of the paper.

Reviewer #3

(Remarks to the Author)
Overall, these studies provide new insight into the complex regulatory mechanisms that control myeloid cell function. The observation that eosinophils interact with monocytes and regulate their function is intriguing and may help to understand diseases where eosinophils are more abundant. The authors provide additional data to support their claims and have qualified a number of their conclusions with discussion points and context. While the methodology is sound, it is difficult to appreciate the biological significance as manipulating these cell types in vivo has various caveats (as the authors note).

Version 3:

Reviewer comments:

Reviewer #1

(Remarks to the Author)
This revision answers most of the questions posed previously. The paper has a high density of interesting and novel findings; however, the Ccl11-centric mechanistic conclusions of how Ackr2 regulates SPM content in peritoneal cavity and function are still not persuasive. There remain a large number of points requiring clarification, including missing controls for the newly added experiments.

Major points:

1. There is no statement indicating that the experiments were done using littermate controls. This is important since

differences in microbiome between strains, which can markedly influence immune responses and disease susceptibility, are generally erased by using littermates, and should be standard practice.
2. Figure 2e, lines 157-159. The description of this experiment is inaccurate. First, supplemental Figure 7 should be described here, not until line 187. Second, although the WT/clodronate results may be consistent with SPMs being important in control of E. coli in peritoneal cavity, their relative contribution cannot be assessed since no clodronate depletion data are shown for LPMs in Supplemental figure 7. Moreover, the data are very noisy and appear to be from one experiment with 7-8 mice, so not robust. Third, the KO/clodronate results are uninterpretable; this is a non-experiment, since SPMs were not depleted in the KO peritoneal cavity.
3. The section starting on line 361 and the corresponding Figure 6 have the following issues:
a. Line 361: this title overstates the data. The mechanism by which Ccl11 is modestly increased in bone marrow is not established. It is consistent with the scavenging function of Ackr2 among other possibilities (increased transcription, translation, producing cells, all of which might be affected by microbiome differences, etc), just as the decrease in Ccl5 levels might result from multiple indirect mechanisms as discussed for Ccl5 by the authors. They measured Ccl5 mRNA and found a decrease. What about Ccl11 mRNA? Whether the small difference in level from WT is enough to account for the differences in eosinophil content in bone marrow and blood is not defined.
b. Figure 6ai and ii are not called out in the text
c. Figure 6aiii, iv and vi: the background signal for KO is ~50% of the signal for WT making any statements about specific functional Ackr2 expression on these cells unreliable.
d. Line 366 and Figure 6avi: need to point out that the mostly negative data for Ackr2 on B1b cells in bone marrow are opposite to those shown in Figure 3 for B1b cells from peritoneal cavity. Also, for panel vi, same problem regarding poor signal to background ratio, particularly since WT signals for B1a and B1c are much higher than for B1b yet are non-specific (not reduced in KO). Not robust or convincing regardless of the RNA data, which can differ from protein data as the authors found for HSCs. Are the B1 RNA data from B1b or total B1?
e. The figure 6a legend is not grammatically correct and is confusing as written. Needs to be rewritten.
f. Figure 6bi and ii: background in KO is very high compared to signal in WT. Not reliable results.
g. Figure 6di: Luminex chemokine levels are described for bone marrow cell lysates, which measure total bone marrow content, not the relevant extracellular levels. Are there differences between WT and KO chemokine levels in the more relevant bone marrow extracellular fluid, particularly for Ccl11? Also, why would evidence of chemokine scavenging, however little, be detectable for only two ligands for Ackr2, Ccl11 and 12?
h. Line 400 and Figure 6diii: given the kd for Ccl11 binding to Ccr3, it is a stretch to conclude that a 0.3 pM difference in Ccl11 concentration in wt vs ko serum has any biological significance. Pharmacologic Ccl11 injection results do not help clarify this, as acknowledged on line 418. Is Ccl11 increased in bone marrow from the conditional mac-specific Ackr2 KOs?
i. Line 423: Do Ccr3 ko mice have normal or increased SPM numbers in peritoneal cavity?
4. Figure 7f: what is the level of eosinophil depletion by this protocol in bone marrow? Also, since the proposed mechanism relates to monocyte-derived macrophages, why would resident, monocyte-independent LPMs be so strongly affected by eosinophil depletion?

Minor points:

1. Line 86: given the differences between SPM and LPM, it's important to add here that LPM derive from embryonic yolk sac/fetal liver, not monocytes.
2. Line 98-99: are these all resident, non-monocyte derived macrophages?
3. The symbol code for PBS and E. coli needs to be inserted into Figure 2C
4. Line 278: the call out of 'Figure 4c iv and v' should be Figure 4a iv and v.
5. Lines 336-337: One obvious point to address is whether the decrease in Ccr5 might explain decreased levels of SPMs in PC.

Version 4:

Reviewer comments:

Reviewer #1

(Remarks to the Author)
Reviewer #1 (Remarks to the Author):
In their response, the authors indicate the following:
1. that the study was conducted using non-littermates with confirmation in Figure 6 of the main phenotype (reduced peritoneal cavity macrophages, reduced bone marrow eosinophils) using macrophage specific knockout of Ackr2 and littermate controls.
2. They agree with the criticism raised that 'The mechanism by which Ccl11 is modestly increased in bone marrow is not established. It is consistent with the scavenging function of Ackr2 among other possibilities (increased transcription, translation, producing cells, all of which might be affected by microbiome differences, etc),...
3. that the evidence for 'specific functional Ackr2 expression on' bone marrow macrophages is suggestive, and not definitive.
4. that it would be unethical to rederive their conditional Ackr2 kos to ascertain whether they phenocopy the increased Ccl11 protein in bone marrow observed in total kos, since this was not originally requested and is therefore a 'supplemental' request.
5. in response to my skepticism about the biological significance of 'a 0.3 pM difference in Ccl11 concentration in wt vs ko serum, the authors respond that 'These concentrations have been expressed as pg/mg of tissue and as such the molarity

cannot be measured.
6. in response to the minor point raised 'One obvious point to address is whether the decrease in Ccr5 might explain decreased levels of SPMs in PC.' The authors respond 'We do not present data on a CCR5 reduction in the peritoneal cavity in the manuscript.'

Overall, the authors have provided adequate caveats in the Results section to address the limitations in the data that limit the Conclusions that can be drawn. However, they have not done this in the Title, Abstract or Discussion, which still overstate the findings for the following reasons:

1. Regarding point 1, the use of non-cohoused, non-littermate wt controls in studies of genetically modified mice is notorious for the introduction of potential artefacts due to genetic drift between the wt and ko strains. This has been substantially mitigated by use of macrophage-specific Ackr2 ko mice, and RNA and some functional evidence that Ackr2 is functionally expressed in macrophages.

2. The use of the word egress to describe the effect of ACKR2 deficiency on absolute eosinophil levels in the bone marrow is not supported by the data, which just present levels at a single timepoint. Experiments to justify attributing the changes to dynamic effects on egress would include effects of Ackr2 blocking agents on mobilization and ideally direct imaging of eosinophil movement. There are multiple other possible mechanisms for why eosinophil levels may be lower in ko mice that were not addressed. Thus, to attribute the differences to an egress mechanism is not justified by the Ccl11 data, including the Ccl11 challenge data which are not really relevant to the conditions these mice find themselves in (low levels of Ccl11 in bone marrow and blood, small differences in those levels between ko and wt).

3. The transcriptomic data do not address whether the differences found between wt and ko macrophages are due directly to eosinophils. The data in Figure 7 only address a couple of macrophage proteins and show very small effects in vitro when macs are exposed to eosinophils.

4. Regarding point 2 above, despite the agreement the authors still attribute the changes they measured for Ccl11 in bone marrow to scavenging in the abstract and discussion. Without measurement of extracellular Ccl11 levels in bone marrow from wt and straight ko, without any measurement of Ccl11 in flox and LysM cre mouse bone marrow, and given the very small changes observed in the straight ko vs wt for Ccl11 levels in bone marrow and for Ccl22 uptake by macrophages, attributing the decreased eosinophil content of the ko bone marrow to the loss of Ackr2 scavenging of Ccl11 is unjustified.

5. Regarding point 5 above, the authors have not answered the question, which was about the minor difference in molarity of Ccl11 that they reported for wt vs straight ko in serum. I indicated a 0.3 pM difference but it's actually more like 20 pM. The authors apparently misread this because there response addresses measurements of tissue in pg/mg. The biological significance of such a small difference in serum is uncertain.

6. Regarding point 6 above, I incorrectly mistakenly wrote Ccr5 when the relevant molecule in the paper was Ccl5.

As mentioned in previous reviews of this paper, there is a high density of interesting and novel findings. That remains true; however, the mechanistic conclusions in this revision remain overstated.

**Open Access** This Peer Review File is licensed under a Creative Commons Attribution 4.0 International License, which permits use, sharing, adaptation, distribution and reproduction in any medium or format, as long as you give appropriate credit to the original author(s) and the source, provide a link to the Creative Commons license, and indicate if changes were made.
In cases where reviewers are anonymous, credit should be given to 'Anonymous Referee' and the source.
The images or other third party material in this Peer Review File are included in the article's Creative Commons license, unless indicated otherwise in a credit line to the material. If material is not included in the article's Creative Commons license and your intended use is not permitted by statutory regulation or exceeds the permitted use, you will need to obtain permission directly from the copyright holder.
To view a copy of this license, visit https://creativecommons.org/licenses/by/4.0/

NCOMMS-24-78775

REVIEWER COMMENTS

***Reviewer #1 (Remarks to the Author):***

***This paper delineates the effect of the atypical chemokine receptor Ackr2 on macrophage differentiation. The authors find a very narrow effect on a subpopulation of small peritoneal macrophages (SPMs). They show that an E. coli challenge to the peritoneal cavity is not normally controlled possibly due to the SPM deficit. They show that ACKR2 deficiency is unlikely to act locally in the PC to cause SPM deficiency but rather remotely in the bone marrow where they document small changes in chemokine and eosinophil levels. They provide some data supporting eosinophil modulation of SPM differentiation ex vivo. Overall, the results are creative and novel and integrate events at the molecular, cellular and immunologic/clinical levels. The authors show a major effect of ACKR2 deficiency on monocyte differentiation at the transcriptome level, so that the effect limited to SPM that they found is somewhat surprising. Possibly additional investigations would reveal other important abnoralities in myeloid cells. The paper would be strengthened by direct tests of eosinophil or CCL11 modulation of SPM differentiation and distribution in vivo.***

1. ***Figure 1: What do SPM in peritoneal cavity and MHCII neg interstitial macrophages in lung share that other macrophage populations tested do not share that might explain their shared dependence on ACKR2 function to attain normal levels in these organs?***

The reviewer(s) raise an interesting point. Surprisingly, SPMs and MHCII negative lung macrophages do not appear to be similar. They have distinct expression of many macrophage cell surface markers as shown in new Supplementary Figure 5. They express different levels of CD11b, MerTK, CD226 and MHCII but share low expression of F4/80.

We also carried out RNA sequencing of MHCII negative interstitial macrophages from WT and ACKR2-/- mice to investigate whether there are shared commonalities

in differentially expressed genes between SPMs and MHCII negative lung macrophages. Again, unexpectedly, except for ACKR2, there are no differentially expressed genes in common between MHCII negative interstitial macrophages and SPMs from WT and ACKR2-/- mice. This data has been added to Figure 5c.

We have added discussion of these points in lines 98-103 and 299-306.

“Surface marker expression was distinct between the macrophage populations controlled by ACKR2 (Supplementary Figure 5). SPMs are $CD11b^{high}$, $MerTK^{+}$, $CD226^{+}$ and $MHCII^{+}$, whereas the interstitial macrophages are $CD11b^{low}$, $MerTK^{-}$, $CD226^{-}$, and $MHCII^{-}$ suggesting the cell types are not similar, despite their shared dependence on ACKR2 (Supplement Figure 5).”

“We also carried out RNA sequencing of FACS sorted MHCII negative macrophages from the lungs of WT and ACKR2-/- mice. Again, we observed a small number of specific transcriptional changes in mature macrophages, including 6 upregulated and 5 downregulated genes (Figure 5c). Except for ACKR2, there were no differentially expressed genes in common between MHCII negative lung macrophages and SPMs from WT and ACKR2-/- mice (Figure 5). This suggests that ACKR2 deficiency results in limited but specific differential gene regulation in macrophages in different tissues.”

***2. Line 152-154: the increase at 18 hours could just reflect the increased number of both SPM and LPM in ko vs wt as shown in Figure 2c iii and iv.***

We agree with the reviewer(s) and have added this statement (Lines 159-161):

“likely due to increased levels of SPMs and LPMs remaining in the peritoneal cavity of ACKR2-/- mice 18h after challenge (Figure 2ciii, vi)”

***3. Figure 3f: the results in vitro in part f are not consistent with the results in vivo in part e, neither at the level of % ECP+ cells which differ by more than an order of magnitude, nor by the ACKR2 dependence which is positive in f and negative in e. I don't see how the in vitro results explain the in vivo findings.***

*In vivo* experiments are likely to be affected by cell migration into the cavity and the macrophage disappearance/ adherence reaction, which occurs in response to inflammation and infection. We have shown cell numbers are altered between WT and ACKR2-/- mice during infection (Figure 2c). This makes it challenging to detect differences in ECP phagocytosis in macrophage populations from WT and ACKR2-/- mice in *vivo*. We believe this accounts for the difference in percentage uptake between our *in vitro* and *in vivo* results.

When cell numbers are constant *in vitro*, we observe an increased percentage of ECP positive macrophages from ACKR2- /- mice, due to deficient elimination of the particles. We have added clarification in lines 151-155, 164 and 168-170:

"suggesting there was no defect in initial binding or uptake between SPMs or LPMs from WT and ACKR2-/- mice *in vivo* (Figure 2ei, iv). However, cell migration and the macrophage disappearance/adherence reaction, which we have shown are altered in ACKR2-/- mice *in vivo* (Figure 2c)*,* are likely to affect this observation."

"When macrophage numbers are constant in vitro"

"When ECP particles are phagocytosed and destroyed by the macrophage, fluorescence is lost, therefore a higher percentage of ECP positive cells indicates deficient phagocytosis."

***4. Figure 3: if ACKR2 functions as a chemokine scavenger in vivo, the levels of ACKR2 ligands should differ between wt and ko mice in vivo. That they don't in the peritoneal cavity may mean that ACKR2 expression is so low as to be trivial in that compartment.***

We agree with these points and have added this clarification and discussion in lines 216-219, and 224-225:

“When ACKR2 actively scavenges *in vivo*, the levels of ACKR2 ligands are altered between WT and ACKR2-/- mice. That we have not observed any change in ACKR2 ligands, suggests that ACKR2 expression is low and not functionally relevant in the peritoneal cavity.”

“indicating that ACKR2 is not actively scavenging ligands during peritoneal cavity infection. These data imply that the SPM defect in ACKR2-/- mice relates to mechanisms operating outside the peritoneal cavity.”

***The in vivo results with the CCL22 uptake assay suggest that may be the case since the receptor is not expressed on macrophages and is only 1-20% of B1 cell subtypes. B1a and d cells have the most by %. What proportion of PC B cells are B1a and d?***

We have added more description of this data in lines 201-205:

“The highest ACKR2 expression *in vivo* was identified on B1a and B1d cells, with between 10 and 20% CCL22 uptake by these populations, which account for around 30% and 10% of peritoneal cavity B cells, respectively.”

***5. Figure 4: why do the wt monocytes appear to generate more SPMs in the ko background? What are the results for both adoptive transfers for LBMs?***

We have added additional data which confirm there is no difference between WT monocyte differentiation to SPMs in WT and KO backgrounds (Figure 4a ii). LPMs are considered to be resident macrophages, therefore there is little differentiation from monocytes to LPMs. This data has been added to Figure 4 civ and v, and described in lines 254-256.

***6. Did ACKR2 ko affect monocyte levels in blood and bone marrow?***

ACKR2 deficiency does not affect monocyte levels in the blood or bone marrow as shown in Supplementary Fig 6h and i . As *n* was low for blood monocytes, we have added additional data which confirm there is no change (Supplementary Figure 6i).

***6 (continued). What were the levels of CCR2 on wt vs ko monocytes?***

CCR2 transcription was unchanged between WT and ACKR2-/- monocytes. We have added description of this in lines 314-317.

"Transcription of the dominant monocyte chemokine receptor CCR2, was unchanged in bone marrow monocytes, SPMs or MHCII negative lung interstitial macrophages from WT and ACKR2-/- mice (Figure 5)."

***6 (continued). The marked difference in CXCR4 between wt and ko might anticipate a major difference in monocyte distribution that would affect all monocyte-derived cell populations, not the highly selective macrophage abnormality that was observed.***

CXCR4 is decreased in SPMs from ACKR2-/- mice but is not significantly different between WT and ACKR2-/- monocytes, or MHCII negative interstitial macrophages. We have added discussion of this in lines 310-314.

"Notably, CXCR4, a key receptor involved in monocyte distribution[38], is decreased in ACKR2-/- SPMs (Figure 5b). However, there was no significant difference in CXCR4 levels in monocytes or MHCII negative interstitial macrophages, suggesting this is not central to the observed phenotype (Figure 5a, c)."

***7. Figure 6aiii: this evidence for ACKR2 expression on BM macrophages and B1b cells is not convincing. Results are noisy for macrophages, and the small difference for ko vs wt among cells with a very low signal in the assay is of uncertain significance. Do these cells express ACKR2 RNA?***

We have added data showing ACKR2 transcripts are detected on sorted bone marrow macrophages and B1b cells (Figure 6aiv and vi), described in lines 336-338.

"We confirmed ACKR2 was transcribed by macrophages and B1b cells, by performing qRT-PCR analysis on FACS sorted bone marrow cells (Figure 6aiv and vi)."

***8. Figure 6b: the authors focus on CCL11, but there are also other differences that are not mentioned including CCL5, which is also an eosinophil chemoattractant acting through the same receptor, and CXCL12, which is an important regulator of distribution for most types of leukocytes. The net result of all these changes on any one cell type are therefore complex to define and not defined in this study.***

We concentrated on CCL11 as it is an ACKR2 ligand, and is detected at increased levels in ACKR2-/- bone marrow, indicative of defective scavenging. We agree with the reviewer(s) that we should have described the other ACKR2 ligands which are affected, and have added discussion in lines 345-355. We did not investigate CXCL12 in this study as it is not an ACKR2 ligand. We have reformatted this figure to increase the legibility of the labels (Figure 6).

“The ACKR2 ligand, CCL12 is increased in the plasma of ACKR2-/- mice, suggesting there is scavenging by vascular ACKR2 in WT mice (Figure 6b ii). However, there is no change in CCL12 levels within the bone marrow of ACKR2-/- mice (Figure 6bi), or in the number of monocytes which express the receptor for CCL12, CCR2, in the blood or bone marrow (Supplementary Figure 6h, i). CCL5 is also an ACKR2 ligand which is altered in the bone marrow (Figure 6bi), but decreased levels were detected in ACKR2-/- mice, indicating that ACKR2 is not scavenging CCL5 in this context. RNA sequencing revealed decreased levels of CCL5 transcription by monocytes in ACKR2-/- mice, which is likely to account for reduced levels of CCL5 protein in ACKR2-/- blood and bone marrow (Figure 5).”

***8 (continued). Moreover, all of the differences are quite small in magnitude and not all in the same direction so of uncertain significane.***

We have hopefully clarified this with the added description of this data (lines 345-355). We believe the key finding is that CCL11 is not scavenged, and is increased in the bone marrow and blood of ACKR2-/- mice. The Cohen’s d values of these data are 1.33 and 1.95, respectively, indicating a large effect size.

***8 (continued). Injection of 1 microgram of CCL11 is a pharmacologic challenge that may not inform the impact of such small changes in CCL11 in the bone marrow.***

We agree with the reviewer(s) and have added this caveat to our description of the results in lines 365-368.

"It is important to note that a high dose of CCL11 was used, due to the known short half-life of chemokines *in vivo* [41], however this may not inform the impact of small changes in CCL11 in the bone marrow."

***9. Figure 7: A direct test of the physiologic relevance of CCL11 to SPM differentiation and localization in PC would be to evaluate SPM in peritoneal cavity in CCL11 and/or CCR3 ko mice. A direct in vivo test of the importance of eosinophils in SPM differentiation would be to evaluate SPM in peritoneal cavity in eosinophil-deficient mice such as the PHIL and dbGATA lines, or in mice depleted of eosinophils with anti-CCR3.***

We obtained bone marrow from WT and CCR3-/- mice and found that in the absence of the CCR3 receptor, eosinophils accumulate in the bone marrow. In particular, a population of mature SiglecF high eosinophils were identified in CCR3-/- mice which are absent from WT bone marrow. We have added this data in Supplementary Figure 13 and discussion in lines 377-380. We have demonstrated the effect of increased egress of eosinophils and decreased monocyte/eosinophil interactions in ACKR2-/- mice, and the accumulation of eosinophils in CCR3-/- mice would not be expected to alter monocyte to macrophage differentiation.

***10. The results in Figures 6 and 7 show indirect and in vitro results with small effect sizes that are of uncertain causative significance. In panel b, there is no unequivocal effect of eosinophils on CD206 MFI.***

We thank the reviewer(s) for drawing our attention to our incomplete description of this data. Treatment with eosinophils alone does not alter CD206 expression on macrophages in vitro, however when combined with IL-4, treatment with eosinophils

increases CD206 expression in BMDMs from ACKR2-/- mice. We have altered our description in lines 416-418:

“Treatment with eosinophils alone did not alter CD206 expression on macrophages, however when stimulated with IL-4, eosinophil treated BMDMs from ACKR2-/- mice were able to respond to IL-4 at increased levels (Figure 7c).”

***10 (continued). In panel c, the experiment is underpowered with n=4.***

We agree with the reviewer(s) and have added additional data in Figure 7c and d.

***10 (continued). The eosinophil wt and ko results are both lower than the control and ko results are greater than wt as for the control.***

We again apologise for our incomplete description of this data and have added more detail in lines 419-423.

“ACKR2-/- BMDMs have a phagocytic defect which results in a higher percentage of ECP+ cells after challenge, due to incomplete breakdown of the pHRODO particles (Figure 2 fiii). Treatment with eosinophils on day 0 restored the ability of BMDMs from ACKR2-/- mice to destroy ECP after phagocytosis (Figure 7d).”

***10 (continued). Hard to conclude anything solid from this. In panel d, the effect size is very small.***

We have added additional data points. In panel d the effect size of the comparison between WT and ACKR2-/- ECP uptake, is 3.810,  and between untreated and eosinophil treated ACKR2-/- ECP uptake is 2.89, indicating a large effect size.

***10 (continued). The purity of the population for eosinophils should be stated in the figure legend.***

This has been added to line 440.

***10 (continued). Can any other cell type increase these markers, e.g. neutrophils?***

In this experiment we have isolated monocytes and eosinophils to directly study the interaction between these cell types in the absence of other cells, and do not have data on other cell types.

***Reviewer #2 (Remarks to the Author):***
***I co-reviewed this manuscript with one of the reviewers who provided the listed reports. This is part of the Nature Communications initiative to facilitate training in peer review and to provide appropriate recognition for Early Career Researchers who co-review manuscripts.***

***Reviewer #3 (Remarks to the Author):***
***In this manuscript by Wilson et al. the authors describe data from mice deficient in the atypical chemokine receptor, ACKR2, that suggests its role in regulation monocyte function indirectly via eosinophil location/mobilization. First, they find that mice lacking this receptor have reduced numbers of monocyte-derived macrophages in the lung and peritoneal cavity, while observing normal resident macrophages, suggesting an impairment in recruited macrophages. They go on to show that scavenging of the chemokine CCL11 is blocked in ACKR2-/0 The authors provide a compelling data set in support of their overall conclusions, although there are several points that the authors are suggested to consider and address.***

***1. Supplementary Figure 3 is intended to support the idea that the small peritoneal macrophages (SPMs) are reduced and that their replacement is poor (upon clodronate depletion) in the ACKR2 KOs relative to wildtype controls. However, there are no statistical analyses on these data to support this conclusion and a fair amount of spread in the data. Notably, whereas depletion via clodronate is apparent in the wildtype mice it appears that clodronate-mediated depletion is not effective in the ACKR2 KO mice, perhaps because***

***their ability to take up and liberate/digest the liposomes and release clodronate (to cause cell death) is impaired? Nonetheless, the authors conclusion from these data are not well supported.***

We thank the reviewer(s) for this excellent point. We have removed our original conclusion, and instead discussed this data in the context of deficient phagocytosis in lines 174-177.

"In addition, clodronate-mediated depletion of SPMs was effective in WT, but not ACKR2-/- mice, suggesting the ability of macrophages from ACKR2-/- mice to phagocytose, release clodronate and cause cell death is impaired (Supplementary Figure 7)."

***2. The authors claim that there were no changes in macrophages in various tissues (line 100 and Supp. Fig. 5), however there are strong trends for reduced macrophages in each tissue. If the macrophages are not subdivided into MHC+/- and total macrophages were examined, are there reductions? Similarly, there are some differences in monocytes that the are also not discussed (ie, monocytes are increased in the blood).***

We have added additional data points to strengthen these comparisons, and added analysis of CD11b+ F4/80+ macrophages, without subdividing on MHCII expression. We did not observe any additional macrophage populations which are significantly reduced in ACKR2-/- mice (Supplementary Figure 4). We have added additional data which confirms there is no change in blood monocytes in ACKR2-/- mice (Supplementary Figure 6i).

***3. Adoptive transfer experiments in Figure 4a-ii are underpowered. There are two distinct populations in the ACKR2-/- group and with just 4 mice in this group/experiment, the conclusion that there's no difference is not convincing.***

We agree with the reviewer(s) and have added more data points to Figure 4aii, which confirm there is no difference between WT monocyte to macrophage differentiation in WT and ACKR2-/- backgrounds.

***4. Figure 5 is confusing and there are some issues with labeling that made it hard to follow and interpret. (1) The authors show wt followed by ACKR2-/- in most figures and panels but then reverse this in panel (e) showing KO and then WT (unless these labels are wrong?).***

We apologise for our labelling errors throughout this Figure. These have now been corrected.

***(2) In panel (e) the graphs depicting SPMs and LPMs (i and iv) should be clearly labeled.***

Labels have been included.

***(3) In panels within (e) the circles for PBS and E.coli are reversed. It's also unclear what is even being measured in the PBS group? Why would anything come up in this group as positive? It would be an important control, for staining, but likely is not relevant or needed in the graphs.***

We agree that the PBS groups are not necessary in these comparisons and have removed them (Figure 2e). We apologise for our labelling errors throughout this figure, which have been corrected.

***(4) The data in panel (f) do not match the text and labeling provided (WT are labeled as open triangle and ACKR2-/- labeled with closed triangle) and text describes the data opposite to what is shown. Again, there is no conclusion from these data.***

We apologise for our labelling errors which are now corrected.

***5. The authors conclude that CCL11 is responsible for regulating eosinophil retention and mobilization from the bone marrow. They show that increased CCL11 in the bone marrow correlates with reduce eosinophils in this tissue and recombinant CCL11 can cause increased blood eosinophils (Fig. 6. d). However, the percentage by flow is really quite low- can the authors also perform CBCs to show an absolute increase in eosinophils? These data are not convincing and do not show the impact of CCL11 on other cell types.***

We have added additional data which shows the numbers of eosinophils in the bone marrow and blood after challenge with CCL11 (Figure 6d). There was no effect on other cell types in the bone marrow and blood (Supplementary Figure 12, lines 364-365).

***5 (continued). Panel (e) in Figure 6 should be more clearly labeled as bone marrow.***

Label has been added.

***5 (continued). It's unclear why the PBS ACKR2-/- group (only 2 mice) is so different from data shown in c.) i).***

We have added additional data from bone marrow samples from WT and ACKR2-/- naïve and PBS challenged mice (Figure 6e). In ACKR2-/- mice there is an increase in eosinophils in the bone marrow in response to an intra-peritoneal PBS injection alone. We believe this is due to exaggerated inflammation at the injection site in the absence of ACKR2 scavenging of inflammatory chemokines. We have discussed this in lines 373-376.

***6. Data in Figure 7 are quite interesting, but additional methods for identifying monocytes and eosinophils, besides chemokine receptor expression would strengthen the conclusion that the monocytes and eosinophils are interacting and in close proximity.***

We agree with the reviewer(s) and have carried out flow cytometry of the bone marrow from iREP mice, which reveals 80% of CCR2 positive cells are Ly6C+ and

97% CCR3+ are SiglecF+. These data are now shown in Supplementary Figure 14. We have modified our description in lines 403-407.

“Flow cytometric analysis of bone marrow cells of iREP mice revealed that 80% of CCR2 expressing cells are Ly6C+ monocytes, and 97% of CCR3 expressing cells are SiglecF+ eosinophils (Supplementary Figure 14). We were able to visualise CCR2+ and CCR3+ cells in close proximity within the bone marrow (Figure 7a).”

***6 (continued). The monocytes appear to be surrounding eosinophils- is this expected?***

We thank the reviewer(s) for drawing our attention to this. We believe that the region we have chosen for the inset image is not representative of the majority of the CCR2+/CCR3+ cell interactions in the bone marrow. It is likely that the cell surrounding the eosinophil is a macrophage expressing CCR2. We have chosen a different, more representative inset image (Figure 7a).

***6 (continued). Using this mouse and manipulating CCL11 levels would be quite interesting, to determine how CCL11 impacts these contacts. It could help corroborate the data shown in Figure 6.***

We thank the reviewer(s) for the suggestion and have carried out an experiment where CCL11 was injected intravenously, 5 h later bones were obtained for imaging analysis. We counted the number of CCR2+/CCR3+ cell interactions in defined regions of interest. These data have been quantified and added to Figure 7b and described in lines 408-413.

“To investigate whether the ACKR2 ligand, CCL11 affects interactions between CCR2+ and CCR3+ cells, 1 μg CCL11 was injected intravenously, and 5 h later bone marrow was obtained for imaging analysis. CCR2+/CCR3+ cell interactions were counted in defined regions of interest. When CCL11 was injected into the bloodstream, the number of CCR2+/CCR3+ cell interactions within the bone marrow decreased (Figure 7b).”

***Minor:***

***1. Figure legend titles should more accurately reflect the key findings. For example, in Figure 1- the authors should state which macrophage population is decreased in ACKR2-deficient mice as the LPMs are unchanged.***

We have updated the following Figure legend titles:

“Figure 1: Small peritoneal macrophages and MHCII negative interstitial macrophages are decreased in ACKR2-/- mice.”

“Figure 5: Monocytes and macrophages are transcriptionally distinct between WT and ACKR2-/- mice.”

***2. Concluding statements are sometimes missing from the results and the authors main point is not always clear. Especially as some data are not terribly clear or convincing. For example, regarding the first results section, the authors look at many different tissues to examine monocytes and macrophages. In the statement on 101, it’s not clear why the authors write “Importantly”- what is important about the observation? That monocyte recruitment is the same? It just needs to be explained more clearly.***

We thank the reviewer(s) for this observation and have added clarification at the end of this section, in lines 106-107.

“This reveals that ACKR2 deficiency does not affect monocyte migration to all tissues.”

***3. The authors do not comment on whether B1 cells are similar in number between the wildtype and ACKR2 KOs.***

We have added data which shows B1 cells were not significantly different between WT and ACKR2-/- mice. Data is shown in Supplementary Figure 8, lines 204-205.

***It seems important and relevant to also show ACKR2 expression on macrophages.***

We have added qRT-PCR analysis of sorted macrophages from the bone marrow which show that ACKR2 is transcribed by bone marrow macrophages (Figure 6a iv and vi). ACKR2 transcripts were also detected at low levels by RNA sequencing of SPMs and MHCII negative lung macrophages (Figure 5). We have described this in lines 336-338 and 308-310.

“We confirmed ACKR2 was transcribed by macrophages and B1b cells, by performing qRT-PCR analysis on FACS sorted bone marrow cells (Figure 6a iv and vi).”

“ACKR2 expression on SPMs and MHCII negative interstitial macrophages has not previously been detected using the functional CCL22 uptake assay [32], however RNA sequencing revealed low levels of transcription (Figure 5b, c).”

***4. In Supplemental Figure 9 the authors define common myeloid progenitors (CMPs) as being gated through the LSK population, however, CMPs are defined as being Sca-1-negative, so these data are incorrect.***

We apologise for our error, the arrow in the gating strategy was wrongly placed. This has now been corrected (Supplemental Figure 11).

Point to point Revision 2 Wilson *et al*

Reviewer #1 (Remarks to the Author):

*This manuscript requires a lot of clarifications as detailed below. Also, I'm not satisfied with the authors response to point 9 in my original review requesting in vivo evidence that eosinophils affect monocyte to macrophage differentiation (and E. coli burden in the peritoneal cavity). Overall, the associations and in vitro studies performed are of interest but don't provide sufficient in vivo evidence for the main conclusions captured in the title of the paper.*

We apologise for our insufficient explanation in our response to point 9 of the first review. We have fully considered the use of each of the mouse strains suggested to assess the effect of eosinophil deficiency in this model. Eosinophil deficient mice are known to exhibit gut dysbiosis and reduced recruitment of intestinal macrophages (Ignacio A *et al*, Immunity, 2022, 10.1016/j.immuni.2022.05.014), which will impact the peritoneal cavity at rest and during infection. For example, peritoneal macrophages have been shown to leave the peritoneum to contribute to the repair of intestinal inflammation (Honda M *et al*, Nature Communications, 2021, 10.1038/s41467-021-27614-9) thereby extending the immune dysfunction from the intestine to the peritoneal cavity. We are therefore concerned that data would be essentially uninterpretable, making experiments ethically unjustifiable.

Similarly, while we have shown that CCR3-/- mice exhibit an accumulation of eosinophils in the bone marrow, these animals are known to have reduced eosinophil numbers throughout the body, including the gut (Humbles AA, PNAS, 2002, 10.1073/pnas.261462598). The same will be true of anti-CCR3 antibody treated, and CCL11-/- mice in which eosinophil numbers are known to be reduced in the gut (Mishra A et al, JCI, 1999, 10.1172/JCI6560). Therefore, whilst we fully agree that it would be ideal to perform experiments in eosinophil deficient mice, we believe that these would be uninterpretable, which would breach our ethical responsibilities. We have added detailed discussion of these points in the revised text (lines 565-576).

We accept the reviewer's point that our title does not fully reflect our main conclusions and have changed it to: "ACKR2 limits eosinophil egress from the bone marrow to promote monocyte to macrophage differentiation and anti-bacterial immunity"

*1. Figure 2: Figure 2B-D, need to indicate the timepoint of analysis in the figure legend.*

Time points have been added to the figure legend.

*The SPM and LPM dynamics are associations and do not and cannot causally explain the increased bacterial burden in ko mice. The authors describe this appropriately conservatively. However, their description would be improved by drawing attention to the following: there is a 10X increased E. coli burden in ko vs wt PC, yet no difference in burden in ko and wt PCW where the burden is 100-1000-fold greater than PC despite the fact that SPM content in PCW is lower in ko than wt at baseline and SPM exceeds LPM in PCW. Perhaps this is why there is no weight change difference for wt vs ko, i.e. the increase in burden in PC contributes little to the overall burden in the peritoneal compartment (wall plus cavity).*

We agree with the reviewer's observations and have added discussion in lines 518-524.

"It is notable that there is a large increase in *E.coli* burden in the ACKR2-/- peritoneal cavity but not in the ACKR2-/- peritoneal cavity wall, a key site of bacterial clearance, which could explain why there is no difference in weight change between WT and ACKR2-/- animals during infection. This may suggest there is a functional difference between the phagocytic capacity of SPMs in the cavity wall and within the peritoneal cavity, or that macrophages phagocytose bacteria differently in the wall environment."

*2. In panels 2ciii and 2di for the peritoneal cavity and cavity wall PBS controls there is no difference in SPM number between WT and KO in either case. This does not agree with the results in Figure 1aii and 1bii. The absolute numbers of SPM in the PBS control condition for cavity wall in 2di are on average 3-fold lower than shown in Figure 1bii and the absolute numbers for SPM/PBS in the peritoneal cavity are ~10-fold higher than for baseline conditions shown in Figure 1. What would be the mechanism to explain such a large and differential PBS effect on SPM content that effaces the difference between wt and ko found at baseline in Figure 1?*

When WT naïve and PBS injected animals are compared there is a reduction in the number of SPMs in the cavity after injection, that we believe is due to damage at the injection site,

leading to an adherence/disappearance reaction (For Reviewer Figure 1). We do not observe the same reduction in ACKR2-/- animals which suggests the adherence/disappearance reaction is impaired. We have discussed this in the context of infection in Figure 2ciii, lines 140-142. The reviewer is correct that the same is happening, albeit to a lesser extent, in SPMs in response to PBS injection. We have discussed this observation in lines 143-6. We are happy to incorporate this figure in the manuscript if the reviewer thinks it is useful (data already presented in Figures 1 aii and 2ciii).

"When WT naïve and PBS injected animals are compared (Figure 1aii, and 2ciii, respectively), there is also a reduction in the number of SPMs in the peritoneum, after injection alone. We believe this is due to damage at the injection site, leading to a disappearance reaction in WT mice, again not observed in ACKR2-/- animals."

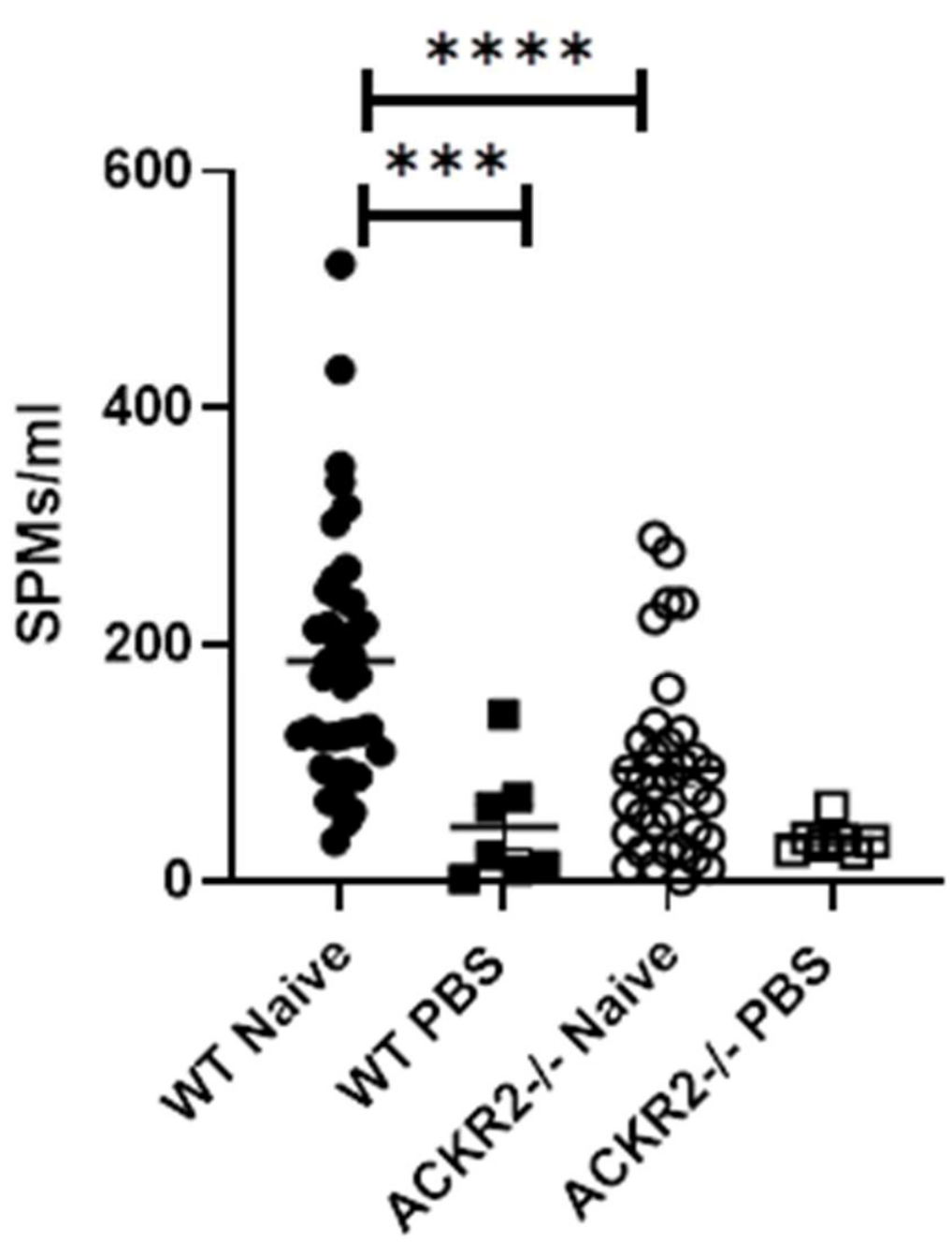


**For Reviewer Figure:** SPM numbers in WT and ACKR2-/- peritoneal cavity, in naïve and 18 h post PBS injection. WT naïve black circles, *n*= 40, WT PBS black squares, *n*=7, ACKR2-/- naïve, *n*=35, ACKR2-/- PBS, *n*=7. Data already presented in Figures 1 aii and 2ciii.

*3. Figure 2E: how is it possible for there to be ~80% ECP+ SPM in both ko and wt at 1 hour (panel 2ei) AND there to be ~200 and 100 SPM/ml at baseline in wt and ko PC, respectively (Figure 1), YET only 5 and 1 ECP+ SPM in wt and ko PC at this timepoint (panel 2eii)?*

*Does the PC content of SPM decline that quickly, and to a greater extent for ko than wt at 1 hour (in contrast to 18 hours)? Can't tell from the rest of the figure, where the timepoint is apparently 18 hours.*

We are grateful to the reviewer for noticing this. The cell counts for the 1 h timepoint were unusually low. We have now repeated this experiment and added new data for the 1 h timepoint (Figure 2ei-ii).

*Also, need to clarify in the figure legend whether the data are for PC or PCW or both.*

Labels have been added.

*The numbers don't align for LPM either comparing 2civ and 2evi.*

The cell counts in this experiment were unusually low. We have repeated this experiment and added the new data (Figure 2eiv-v).

*4. Figure 2f: clarify that the cells are from naïve mice (not infected). Also, for 2fiii the timepoint for BMDM is not specified in the text or the figure legend, unlike the other 2f panels.*

We apologise for our omission and have added this information.

*Point out that there is a massive difference in %+ SPM from PC in vitro vs in vivo. Why would that be? Are the cells more efficient in vitro at breaking down PHRODO? It's not clear what you are concluding from these PHRODO studies. If the ACKR2 ko cells have a phagocytic defect, would that not also be relevant in the PCW where there is no difference in bacterial burden between ko and wt?*

We agree with these points and have added discussion in lines 181-185, and 518-524.

“ECP particle breakdown by peritoneal macrophages appears to be more efficient *in vitro* than *in vivo* (Figure 2e-f), which may be due to a number of factors such as different culture conditions, enhanced proximity of ECP particles to the cell *in vitro,* and cell turnover *in vivo*.”

“It is notable that there is a large increase in *E.coli* burden in the ACKR2-/- peritoneal cavity but not in the ACKR2-/- peritoneal cavity wall, a key site of bacterial clearance, which could explain why there is no difference in weight change between WT and ACKR2-/- animals during infection. This may suggest there is a functional difference between the phagocytic capacity of SPMs in the cavity wall and within the peritoneal cavity, or that macrophages phagocytose bacteria differently in the wall environment”

*5. Figure 3d: the symbols are not defined.*

These symbols have now been defined.

*6. Figure 4 legend needs more detail. E.g. panel b doesn’t indicate what cells were cultured (sorted BM monocytes?); panel bii y-axis indicates the unit but not the parameter (MHCII expression). Panel c doesn’t indicate what cell type is treated with IL-4 and lps and for how long and doesn’t specify what the symbols refer to (wt and ko but which is which?).*

We apologise for the incomplete description of this Figure. The legend has been updated.

*7. Line 268: there is nothing in the Introduction or the Results to this point that would explain the motivation for testing CD80 and CD206 or the significance of the results obtained. This should be clarified here.*

We thank the reviewer for drawing our attention to this and have introduced this in lines 273-277.

“To investigate whether BMDMs generated from WT and ACKR2-/- mice are altered in their ability to become either pro or anti-inflammatory macrophages, they were stimulated with either LPS or IL-4 overnight on day 5 of culture, and expression of the classical activation marker, CD80 or the alternative activation marker, CD206, was assessed.”

*8. Figure 6av and 6bi and ii: the open symbols are defined as ACKR2, not ACKR2 ko.*

This has been corrected.

*9. Figure 6: panels i and ii are not described or called out in the Results section. The evidence for ACKR2 expression on BM macrophages and B1b cells is not convincing. For B1 cells, there is a very small effect size for decreased % CCL22 uptake positive B1b cells in ko compared to wt shown in panel 6av. And only 1-2% of B1b's are positive in this assay. Moreover, B1a and B1c cells have much higher levels of uptake positive cells than B1b's without any decline in kos and without any data shown for RNA in panel 6avi, raising questions as to whether the B1b signal is really just noise. Likewise, the positive macrophages represent only ~12% of cells and the specific signal is extremely small. Can the authors show MFI data that would more convincingly demonstrate a signal against the noise in these few 'specific' uptake positive cells?*

The reviewer is correct that the B1b expression is likely to be irrelevant. We have generated an ACKR2 flox mouse crossed with LysM Cre to delete ACKR2 specifically on myeloid cells. Data has been added to the manuscript which shows that CCL22 uptake and MFI is reduced in bone marrow macrophages obtained from these mice, compared with WT (Figure 6 bi-ii). Importantly, macrophage expressed ACKR2 is sufficient to maintain eosinophil levels in the bone marrow (Figure 6 eii). SPMs are also reduced in ACKR2 flox/LysM cre mice, revealing that ACKR2 expression on macrophages and not B1b cells in the bone marrow is required for the phenotype described in this manuscript (Figure 6c). We have updated the manuscript in lines 361-367 and 389-393, and the schematic in Figure 8, to reflect this.

"To test the role of ACKR2 specifically on macrophages, we generated an ACKR2 flox mouse crossed with LysM Cre to delete ACKR2 specifically on myeloid cells. Again, CCL22 uptake is reduced in bone marrow macrophages obtained from these mice, compared with ACKR2 flox control mice (Figure 6bii). Importantly, SPMs are also reduced in ACKR2 flox/LysM cre mice (Figure 6c), revealing that ACKR2 expression on macrophages and not B1b cells in the bone marrow is required for this phenotype."

*"*When ACKR2 is deleted specifically on macrophages, eosinophils are similarly reduced in the bone marrow, revealing that macrophage expressed ACKR2 is sufficient to maintain eosinophil levels in the bone marrow (Figure 6eii)."

*10. Figure 6b: likewise, the CCL11 expression signal in BM is quite small, a mean difference of 1 vs 2 pg/mg in bone marrow with high variance. CCR3 is known to have about 12 chemokine ligands. Can the authors find one whose expression is robustly affected by ACKR2 ko?*

We thank the reviewer for this suggestion. We have focused on the chemokine ligands shared between ACKR2 and CCR3; CCLs 4, 5, 7, 11 and 13. We carried out Luminex analysis of CCLs 4, 5, 7, and 11, but had not investigated CCL13. We have now added CCL13 ELISA data, which shows there is no difference in CCL13 protein concentration between WT and ACKR2-/- mice in the bone marrow or plasma (Figure 6dii, iv, lines 406-409)

"ACKR2 shares 5 ligands with the eosinophil receptor CCR3; CCLs 4, 5, 7, 11 and 13. We carried out Luminex analysis of CCLs 4, 5, 7, and 11, and ELISA of CCL13. Only CCL11 showed increased levels in bone marrow, consistent with deficient scavenging in ACKR2-/- mice."

*11. Figure 6d: what is the effect of injecting CCL11 into ACKR2 ko mice?*

We have added data which shows that i.v injection of CCL11 into ACKR2-/- mice does not further increase eosinophil egress from the bone marrow (Figure 6gi). Intriguingly, eosinophils are reduced in the bloodstream of ACKR2-/- mice after CCL11 injection suggesting there is increased recruitment of eosinophils elsewhere within the body (Figure 6gii, described in lines 399-403).

"CCL11 injection into ACKR2-/- mice does not further increase eosinophil egress from the bone marrow (Figure 6gi). Intriguingly, eosinophils are reduced in the bloodstream of ACKR2-/- mice after CCL11 injection suggesting there is increased recruitment of eosinophils elsewhere within the body (Figure 6gii)."

*12. Figure 6e legend: indicate how many hours after PBS injection the eosinophil levels were assessed. It's hard to imagine that PBS injection would have sustained effects on eosinophil*

*levels in BM only in the ko whereas E. coli infection would not. The authors speculate the changes are due to effects on egress. What are the associated blood eosinophil levels?*

We agree with the reviewer that this observation is incomplete. As these data are not central to our mechanism we have removed them.

*13. Line 399-400: this overstates the evidence in the figure. BM eosinophil levels appear to be ACKR2-dependent and CCL11-mobilizable. The precise mechanism for altered eosinophil levels in ACKR2 ko BM is still unclear.*

We agree and have altered our description in lines 438-439.

*14. Figure 7: while the effect of CCL11 on interacting cells is of interest, of greater interest is how the density of interacting cells is affected by ACKR2 ko.are  And while the in vitro studies are of interest, of greater interest is whether animals lacking or overconcentrating eosinophils in bone marrow would have abnormal monocyte to macrophage differentiation in vivo, resulting in altered levels of SPM in PC and PCW. For example, how are PC and PCW SPM and LPM levels and E. coli burden affected in CCL11 ko mice, CCR3 ko mice, PHIL mice, dbGATA mice? I don't understand the logic for the response to point 9 in the original review.*

Again we apologise for our insufficient explanation in our response to this concern in the first review. We agree that studying animals lacking or overconcentrating eosinophils in bone marrow would add valuable insight. We have carefully considered the use of each of the mouse strains suggested to directly assess the effect of eosinophil deficiency and accumulation in this model. However, each of the current models available to do this would cause gut dysbiosis and immune alterations in the peritoneal cavity, which would render our results uninterpretable, making experiments ethically unjustifiable. We have added detailed discussion of these points in the revised text (lines 565-576).

Reviewer #3 (Remarks to the Author):

*Gillian et al. responded to the reviewer comments by providing new data and modifying some conclusions and statements. Overall, the editorial changes and corrections and additions improved the manuscript, though there are still some issues that should be addressed. The key findings of this manuscript are that eosinophils and monocytes interact in the bone marrow and that these interactions impact monocytes in such a way that they are functionally and numerically different in specific sites like the peritoneal cavity. The concept is quite interesting, but the biological significance is not clearly articulated.*

We thank the reviewer for pointing this out and have added additional discussion of the biological significance in lines 577-585.

"This study demonstrates a novel mechanism whereby the atypical chemokine receptor, ACKR2 maintains a stable level of eosinophils in the bone marrow niche to facilitate key eosinophil and monocyte interactions. Without these interactions, monocytes are transcriptionally altered and are impaired in their ability to differentiate into macrophages. Upon differentiation, these macrophages are impaired in their ability to phagocytose bacteria during infection. Taken together, this study demonstrates for the first time that eosinophils play a key role in monocyte education in the bone marrow, which contributes to defence against bacterial infection."

*There are some trends that are obvious, and although the changes don't reach significance, the authors conclusions are not convincing. For example, there appear to be changes in the omentum that are dismissed and overlooked. There are also striking sex differences in B1 cells that are not discussed.*

We agree that there appear to be some trends in the macrophage data from the omentum, however they do not reach statistical significance. We have added p values to each of the omentum panels in Supplementary Figure 4.

*1. There are also striking sex differences in B1 cells that are not discussed.*

We apologise for our inaccurate description of this data. While there are no significant differences in the number of B1 cell subsets between WT and ACKR2-/- animals there are

striking differences between male and female WT B1a, B1b and B1c cells that are not observed in ACKR2-/- animals. We have added statistical analysis and described this observation in lines 214-6.

“however there was a striking increase in B1a, b and c cells between WT male and female mice, that was not observed in ACKR2-/- animals (Supplementary Figure 8).”

*2. The authors rightfully focus on chemokines, as the ACKR2 receptor is known for scavenging chemokines, but they ignore the role of other factors that are likely going to be impacted by a change in chemokine availability. The authors do not discuss why CCL5 production would be decreased in the ACKR2-/- mice aside from a role of monocytes which are not likely the main source of CCL5 in the bone marrow. Did the authors examine megakaryocytes and T cells in the bone marrow? Did they assess platelets in circulation?*

We have added data in new Supplementary Figure 12 which shows that the number of megakaryocytes and T cells were unchanged between WT and ACKR2-/- bone marrow. In addition the number of platelets in circulation were unaltered in the absence of ACKR2. This has been discussed in lines 381-384.

“As CCL5 is produced by T cells and megakaryocytes in the bone marrow and by platelets in the blood we compared the number of these cell types, and found no significant differences between WT and ACKR2-/- animals (Supplementary Figure 12).”

*The imaging studies that demonstrate interactions between CCR3+ and CCR2+ cells in the bone marrow are interesting though not entirely convincing. There appear to be interactions in the CCL11 treated mice as well….*

Unfortunately, due to the short half-life of chemokines *in vivo*, it was not possible to fully deplete eosinophils in the bone marrow (Erickson et al, J Pharmacol Exp Ther, 2014, 10.1124/jpet.114.213074). We have added this caveat in lines 452-453.

“Due to the short half-life of chemokines *in vivo*, it is not possible to fully deplete eosinophils in the bone marrow”

*and there also seem to be more CCR2+ cells in these images? Would that suggest that there are more monocytes? They do not show data for this, only eosinophils.*

In Figure 7biii we have counted only the interactions between CCR2 and CCR3 expressing cells. We carried out flow cytometry of the iREP mice after i.v injection of PBS or CCL11 which showed the number of cells expressing CCR2 were unchanged (data not shown). We agree that our PBS image appeared to have less CCR2 expressing cells and have included more representative images (Figure 7bi-ii).

*4. Why did the authors not deplete eosinophils and/or use models to investigate how increasing eosinophils would impact monocytes to more directly assess the in vivo role of eosinophils in modulating monocytes in the bone marrow?*

We agree that studying animals lacking or overconcentrating eosinophils in bone marrow would add valuable insight. We have carefully considered the use of each of the mouse strains suggested to directly assess the effect of eosinophil deficiency and accumulation in this model. However, each of the current models available to do this would cause gut dysbiosis and immune alterations in the peritoneal cavity, which would render our results uninterpretable, making experiments ethically unjustifiable. We have added detailed discussion of these points in the revised text (lines 565-578).

"We considered the use of eosinophil deficient mouse strains to assess the effect of eosinophil deficiency in this model. However, eosinophil deficient mice are known to exhibit gut dysbiosis and reduced recruitment of intestinal macrophages [56] which will impact the peritoneal cavity at rest and during infection. For example, peritoneal macrophages have been shown to leave the peritoneum to contribute to the repair of intestinal inflammation [57], thereby extending the immune dysfunction from the intestine to the peritoneal cavity. This would render the data uninterpretable. Similarly, while we have shown that CCR3-/- mice exhibit an accumulation of eosinophils in the bone marrow, these animals are known to have reduced eosinophil numbers throughout the body, including the gut [58]. The same would be true of anti-CCR3 antibody treated, and CCL11-/- mice in which eosinophil numbers are known to be reduced in the gut [59]."

*It is unclear if the receptor is intrinsically required by monocytes for the observed changes. The adoptive transfer experiments were a good approach to try to test this, but monocytes*

*were obtained from wildtype and complete knockout animals. Mixed chimeric mice would have helped to address this question (using both WT and ACKR2-deficient hosts). As ACKR2 is thought to be expressed primarily by non-hematopoietic cells, these studies would have helped to directly address the role of ACKR2 in monocytes themselves. As there is a fundamental change in monocytes in the knockouts that results in impaired differentiation, it seems important to address how ACKR2-deficiency itself impairs differentiation programs.*

ACKR2 is not expressed on monocytes themselves, but on macrophages in the bone marrow. We apologise for the confusion and have now stated this explicitly in line 308.

To test the role of ACKR2 specifically on macrophages, we generated an ACKR2 flox mouse crossed with LysM Cre to delete ACKR2 specifically on myeloid cells. Data has been added to Figure 6 eii which shows that macrophage expressed ACKR2 is sufficient to maintain eosinophil levels in the bone marrow. SPMs are reduced in the peritoneal cavity of ACKR2 flox/LysM cre mice, revealing that ACKR2 expression on macrophages and not B1b cells is required for the phenotype described in this manuscript (lines 361-367 and 389-391).

"To test the role of ACKR2 specifically on macrophages, we generated an ACKR2 flox mouse crossed with LysM Cre to delete ACKR2 specifically on myeloid cells. Again, CCL22 uptake is reduced in bone marrow macrophages obtained from these mice, compared with ACKR2 flox control mice (Figure 6bii). Importantly, SPMs are also reduced in ACKR2 flox/LysM cre mice (Figure 6c), revealing that ACKR2 expression on macrophages and not B1b cells in the bone marrow is required for this phenotype."

*"*When ACKR2 is deleted specifically on macrophages, eosinophils are similarly reduced in the bone marrow, revealing that macrophage expressed ACKR2 is sufficient to maintain eosinophil levels in the bone marrow (Figure 6eii)."

*Are there changes in expression of MCSFR on ACKR2-/- monocytes? Are there differences in M-CSF in the knockouts?*

There are no significant differences in transcription of *csf1* or *csf1r* between monocytes from WT and ACKR2-/- bone marrow.

Reviewer #1 (Remarks to the Author):

*The authors have clarified some points and have added important macrophage specific Ackr2 ko data; however, the mechanistic claims made still exceed the strength of the data supporting them, particularly with regard to in vivo evidence, as follows:*

*Lines 125-126 indicate that in Figure 2 the authors 'investigated whether reduced macrophage numbers as a result of ACKR2 deficiency had functional consequences during peritoneal infection with E.coli'. However, the data are not convincing. First, more cell types than macrophages have abnormal numbers in the peritoneal cavity of the total kos, including neutrophils and monocytes as shown in figure 2. However, the experiments are not actually designed to determine which of these alterations, if any, account for the increased E. coli burden in the kos. Infections of LyseM cre X floxed Ackr2 mice would at least allow specific statements of the role of macrophage Ackr2 on E. coli control in the model.*

We thank the reviewer for this suggestion and have added data showing that when macrophages were depleted using clodronate liposomes before *Escherichia coli* challenge, bacterial burden was increased in the peritoneal cavity of WT mice and unaffected in ACKR2-/- mice, indicating that macrophages are key to our observed phenotype (Figure 2e). We have described this data in lines 155-159 and the methodology in lines 705-708.

*Second, unlike in naïve animals, baseline macrophage content after PBS injection is not different for either SPM or LPM in either peritoneal cavity or cavity wall in ACKR2 ko vs WT animals. The authors speculate that this is ''due to damage at the injection site, leading to a disappearance reaction in WT mice, again not observed in ACKR2-/- animals". However, in the same sites putatively damaged by PBS injection there is also no difference in neutrophil or monocyte content, i.e. no convincing evidence of significant damage. A defect in the same macrophage disappearance reaction is invoked to explain why SPM and LPM content is not decreased but instead is increased (i.e. less disappearance) in E. coli-injected ACKR2 ko vs WT animals in the peritoneal cavity, and SPM content is not different in cavity wall of E. coli injected*

*wt vs ko animals. Yet, in the same animals LPM content is decreased in the cavity wall of E.coli injected kos compared to wts, and this is associated with no difference in E. coli burden in the site. Very confusing and inconsistent, and allows for statements about associations, not causality about the functional consequences of reduced macrophage numbers in this infection model.*

We agree that our description of this data was confusing and have modified our description (lines 145-146). We agree that it is a notable point that decreased LPM numbers in the cavity wall of ACKR2-/- does not lead to increased CFU. This suggests that LPMs are not the key cell type for bacterial clearance in the cavity wall. We have added description of this in lines 151-154.

*Line 350: the claim that 'ACKR2 scavenges CCL11 to regulate eosinophils in the bone marrow and blood' is based on associations of very low levels of expression of Ackr2 in macrophages with very small changes in CCL11 and larger changes in eosinophil content in kos, and is not convincing. The levels of CCL11 measured in wt bone marrow are in the femtomolar range, well below the kd for binding to the CCL11 receptor CCR3, and the ko results in only a small increase in that level, still in the femtomolar range.*

This data was normalised to the total protein concentration of tissue samples, as determined by a BCA assay (lines 743-744), therefore the molar concentration cannot be calculated. We agree that the amount of CCL11 per mg of total protein is low and have added this description in lines 380-381. We believe the key finding is that CCL11 is not scavenged, and is increased in the bone marrow and blood of ACKR2-/- mice. Although the pg/mg value of CCL11 is low the Cohen's *d* values of these data are 1.33 and 1.95, respectively, indicating a large effect size.

*Moreover, there are even smaller increases in higher levels of CCL5, a second important CCR3 ligand. There are no data to define the individual or net causal effect of these associated small and opposed expression changes compared to wt on eosinophil content in BM.*

ACKR2 is a scavenging receptor, therefore in ACKR2 deficient tissues elevated protein levels of chemokine ligands indicate defective scavenging. Decreased levels of CCL5 in ACKR2-/- mice are therefore not due to deficient scavenging by the receptor. We apologise for not describing this clearly and have added further description in lines 388-392. Monocytes from ACKR2 deficient mice have decreased transcription of CCL5 which we believe accounts for the decreased levels of CCL5 detected in ACKR2-/- bone marrow (lines 392-394, Figure 5).

*Moreover, the Ccl22 scavenging assay has a very high background as defined in ko macrophages relative to the very low levels of 2-3% +ve macrophages and MFI in wt macrophages. It is not clear that this small difference in CCL11 protein expression in wt vs ko bone marrow is from scavenging differences attributable to the small observed differences in Ackr2 wt vs ko macrophages as opposed to some other mechanism such as small changes in CCL11 gene expression, among other possibilities. Nor is it clear, the CCL11 challenge data notwithstanding, that this miniscule difference in endogenous CCL11 content in ko vs wt accounts for the difference in eosinophil content.*

We acknowledge there is background in the CCL22 binding assay and have confirmed ACKR2 is transcribed by FACS sorted bone marrow macrophages, by carrying out qRT-PCR (Figure 6av). Although the pg/mg value of CCL11 is low the Cohen's *d* value of the change is 1.33 indicating a large effect size.

*Line 478: what is the timepoint for these data? How was a region of interest defined?*

CCL11 was injected for 5 h before bones were collected as described in lines 462-463. We have added this information to the figure legend in line 496. All interactions between CCR2 and CCR3 expressing cells were counted in randomly chosen fields of view.

*Line 437: The claim that 'Eosinophils promote monocyte to macrophage differentiation' is not fully supported. First, Figure 7 does not establish whether the*

*macrophage-interacting eosinophils occur at random. At least, what is the percentage of macrophages that form interactions with eosinophils and vice versa? The distribution of red and blue cells in Figure 7b show very few that are adjacent, maybe just random and of unknown significance. Second, CCL11 injection is known to mobilize eosinophils from bone marrow (figure 6), without affecting macrophages, so it is not surprising that the density of apparent macrophage-interacting eosinophils would decrease after CCL11 injection, even if the apparent interactions occurred at random.*

We apologize that our description was unclear. We have not observed macrophage interactions with eosinophils in the bone marrow. Instead CCR2+ monocytes and CCR3+ eosinophils are located in close proximity in the bone marrow (Figure 7a, Supplementary Figure 15). We have added additional clarification in lines 459-460. We propose that monocytes and eosinophils interact through soluble mediator(s) in the bone marrow as described in lines 484-486 and 584-587.

*Third, figure 7c shows that eosinophils per se have no effect on CD206 expression (as a marker of macrophage differentiation) on cocultured BMDM in vitro and only slightly boost the large increase of CD206 induced by recombinant IL-4. Fourth, eosinophil coculture had no effect on ECP phagocytosis by cocultured wt BMDM and only a minor effect on this parameter for cocultured ko BMDM.*

In Figure 7c and d we did not expect eosinophils to increase phagocytotic activity or CD206 expression in WT BMDM culture. Rather, when the deficit in eosinophil numbers was restored in BMDM cultures from ACKR2-/- mice, we show that eosinophil co-culture rescues the ability of macrophages to break down ECP particles and express CD206 in response to IL-4 stimulation.

*It isn't established that BMDM produced this way express Ackr2 or whether Ccl11 or any other Ackr2 or eosinophil activating chemokine would be present in this system to play a role.*

We believe our phenotype arises from altered cell egress from the bone marrow *in vivo*. The *in vitro* BMDM cultures develop from cells present in the bone marrow at

the time of culture. Additional cell egress does not occur in this system, therefore the active expression of ACKR2 or chemokine ligands would not be expected to further affect the BMDM culture *in vitro*.

*As a clerical point, the figure shows 8 data points for each condition, whereas the figure legend indicates n=4. On line 671, it isn't clear what the numerator and denominator are in the 1:25 ratio. This should be indicated in the figure legend as well along with the duration of the coculture before assay.*

We apologise for these errors which have been corrected. The duration of the monocyte and eosinophil co-culture is 18h (line 504 and line 691).

*Fifth, in figure 7e, there is a small effect size of eosinophil cocultivation with monocytes with regard to expression of Alox15 and TGFbeta; however the induction is not dependent on monocyte-eosinophil contact.*

The Cohen's *d* values of these changes are 2.38 and 4.38 respectively, indicating a very large effect size. The reviewer is correct we do not believe physical contact is required and instead monocytes and eosinophils interact through soluble mediator(s) in the bone marrow as described in lines 484-486 and 584-587.

*As a clerical point, the figure legend indicates n=7 for the control condition, which is correct for Alox 15 but not TGFbeta. The figure does not specify whether the monocytes are from wt or ko mice or why only one genotype was tested.*

We apologise for these errors which have been corrected (lines 504-5). We were investigating whether monocyte and eosinophil co-culture altered gene transcription in WT mice and did not require ACKR2-/- animals.

*For all the in vitro experiments, it isn't clear whether each data point indicates cells from a different mouse or from one mouse, nor whether the data are summary data*

*from multiple experiments or one experiment that is representative of other experiments. Therefore, the data as presented are not clearly reproducible.*

We apologise for this omission which has been corrected (line 501-502).

*Given the above, as mentioned previously, the absence of in vivo evidence that eosinophils promote macrophage differentiation remains a significant limitation of the paper.*

We agree with the reviewer and have added data that shows when eosinophils were depleted using anti-SiglecF antibody treatment, SPMs and LPMs were reduced in the peritoneal cavity (Figure 7f). This data has been described in lines 486-491 and methodology in lines 719-722. The decrease in LPMs may be related to the gut dysbiosis known to be caused by eosinophil depletion. This caveat has been described in lines 588-594.

**Response to Reviewer**

*Reviewer #1 (Remarks to the Author):*

*This revision answers most of the questions posed previously. The paper has a high density of interesting and novel findings; however, the Ccl11-centric mechanistic conclusions of how Ackr2 regulates SPM content in peritoneal cavity and function are still not persuasive. There remain a large number of points requiring clarification, including missing controls for the newly added experiments.*

*Major points:*

*1. There is no statement indicating that the experiments were done using littermate controls. This is important since differences in microbiome between strains, which can markedly influence immune responses and disease susceptibility, are generally erased by using littermates, and should be standard practice.*

Statements on littermate controls have been added in lines 639-641. WT and ACKR2-/- animals were bred as homozygotes, however the macrophage specific ACKR2 deficient mice were bred as littermates, and the central phenotype was observed in both mouse strains.

*2. Figure 2e, lines 157-159. The description of this experiment is inaccurate. First, supplemental Figure 7 should be described here, not until line 187.*

We agree with the reviewer and have moved the description of this data to lines 160-163.

*Second, although the WT/clodronate results may be consistent with SPMs being important in control of E. coli in peritoneal cavity, their relative contribution cannot be assessed since no clodronate depletion data are shown for LPMs in Supplemental figure 7.*

These data have been added.

*Moreover, the data are very noisy and appear to be from one experiment with 7-8 mice, so not robust. Third, the KO/clodronate results are uninterpretable; this is a non-experiment, since SPMs were not depleted in the KO peritoneal cavity.*

We agree that the phagocytic defect of SPMs from ACKR2-/- animals affects clodronate uptake as well as *E.coli* phagocytosis. The question from the reviewer's previous review was which cell type was responsible for the increased bacterial burden in ACKR2-/- animals. This experiment shows that when macrophages were depleted in WT mice, bacterial burden increased to the level observed in ACKR2-/- animals, strongly suggesting that macrophages are the key cell type driving elimination of *E.coli* in this model.

*3. The section starting on line 361 and the corresponding Figure 6 have the following issues:*

*a. Line 361: this title overstates the data. The mechanism by which Ccl11 is modestly increased in bone marrow is not established. It is consistent with the scavenging function of Ackr2 among other possibilities (increased transcription, translation, producing cells, all of which might be affected by microbiome differences, etc), just as the decrease in Ccl5 levels might result from multiple indirect mechanisms as discussed for Ccl5 by the authors. They measured Ccl5 mRNA and found a decrease. What about Ccl11 mRNA? Whether the small difference in level from WT is enough to account for the differences in eosinophil content in bone marrow and blood is not defined.*

We agree with the reviewer and have amended the title in line 364. ACKR2 scavenges at the protein level therefore transcriptional changes may not be observed. We believe the CCL5 reduction is unrelated to deficient scavenging, but rather a fundamental difference due to altered monocyte education in the bone marrow. In the bone marrow, CCL11 has been shown to be produced by stromal cells such as fibroblasts (Zhong *et al*, Int J Biol Sci, 2024) and osteoblasts (Kindstedt *et al*, Scientific Reports, 2017). CCL11 transcription was not detected by RNA sequencing of purified monocytes in either WT or ACKR2-/- animals. We can include this discussion in the text if needed.

*b. Figure 6ai and ii are not called out in the text*

We apologise for this omission and have added the description in line 366.

*c. Figure 6aiii, iv and vi: the background signal for KO is ~50% of the signal for WT making any statements about specific functional Ackr2 expression on these cells unreliable.*

We have altered our description of this data. We have modified the statement from ‘indicating’ to ‘suggesting that ACKR2 is expressed’ in line 368. We have removed the word functional from line 372.

*d. Line 366 and Figure 6avi: need to point out that the mostly negative data for Ackr2 on B1b cells in bone marrow are opposite to those shown in Figure 3 for B1b cells from peritoneal cavity. Also, for panel vi, same problem regarding poor signal to background ratio, particularly since WT signals for B1a and B1c are much higher than for B1b yet are non-specific (not reduced in KO). Not robust or convincing regardless of the RNA data, which can differ from protein data as the authors found for HSCs. Are the B1 RNA data from B1b or total B1?*

It is notable that ACKR2 expression by B1 cells differs between the bone marrow and peritoneal cavity, which is likely to be context dependent. However, as we have described in lines 379-81, B1 cell expression of ACKR2 is likely irrelevant in this context, as macrophage specific ACKR2 deficiency recapitulates the central phenotype. The qRT-PCR data was obtained from B1b cells sorted from the bone marrow (line 369-371).

*e. The figure 6a legend is not grammatically correct and is confusing as written. Needs to be rewritten.*

We apologise for our grammatical errors which have been corrected.

*f. Figure 6bi and ii: background in KO is very high compared to signal in WT. Not reliable results.*

We acknowledge that chemokine uptake assays have high background. To address this we included qRT-PCR data of ACKR2 transcription by sorted bone marrow macrophages (Figure 6av).

*g. Figure 6di: Luminex chemokine levels are described for bone marrow cell lysates, which measure total bone marrow content, not the relevant extracellular levels. Are there differences between WT and KO chemokine levels in the more relevant bone marrow extracellular fluid, particularly for Ccl11?*

We have not looked at extracellular fluid in the bone marrow specifically. We agree that our data does not define whether CCL11 levels are extracellular or intracellular and have added this caveat in lines 386-7. As ACKR2 scavenges extracellularly, including intracellular CCL11 likely leads to an underestimation of the scavenging activity.

*Also, why would evidence of chemokine scavenging, however little, be detectable for only two ligands for Ackr2, Ccl11 and 12?*

ACKR2 has at least 12 chemokine ligands and scavenging is context and spatially dependent. For example, in the pubertal mammary gland CCL11, CCL7 and CCL12 are scavenged (Wilson *et al*, Development 2020), whereas in the lungs of tumour injected mice only CCL2 is scavenged (Albano F et al, J Immunother Cancer, 2025).

*h. Line 400 and Figure 6diii: given the kd for Ccl11 binding to Ccr3, it is a stretch to conclude that a 0.3 pM difference in Ccl11 concentration in wt vs ko serum has any biological significance. Pharmacologic Ccl11 injection results do not help clarify this, as acknowledged on line 418.*

These concentrations have been expressed as pg/mg of tissue and as such the molarity cannot be measured.

*Is Ccl11 increased in bone marrow from the conditional mac-specific Ackr2 KOs?*

Unfortunately we do not have this data and these mice are no longer breeding. We created these mice to answer the central question of whether macrophage specific ACKR2 expression was sufficient to alter SPM levels, and CCL11 data was not requested. From an animal use perspective, we believe it would not be ethical to rederive the colony now to address a supplemental question.

*i. Line 423: Do Ccr3 ko mice have normal or increased SPM numbers in peritoneal cavity?*

In our first revision we showed that eosinophils accumulate in the bone marrow of CCR3-/- mice (Supplementary Figure 14). Unfortunately we do not have data from the peritoneal cavity. As eosinophils are retained in the bone marrow of CCR3-/- mice, we do not believe the effect of CCR3 deficiency on the peritoneal cavity is key for our study or its conclusions. Importantly, while CCR3-/- mice exhibit an accumulation of eosinophils in the bone marrow, these animals are known to have reduced eosinophil numbers throughout the body, including the gut (Humbles AA, PNAS, 2002). Eosinophil deficient mice are known to exhibit gut dysbiosis (Ignacio A *et al*, Immunity, 2022). This impacts the peritoneal cavity as peritoneal macrophages leave the peritoneum to contribute to the repair of intestinal inflammation (Honda M *et al*, Nature Communications, 2021). Indeed, our anti-SiglecF antibody depletion experiments showed an additional, unexpected, reduction in LPMs likely to be due to this dysbiosis (Figure 7fiii). We have discussed this in lines 594-600.

*4. Figure 7f: what is the level of eosinophil depletion by this protocol in bone marrow?*

Anti-SiglecF antibody based depletion of eosinophils in the bone marrow was effective, as previously described in Chu, V.T. *et al*, Nature Immunology, 2011. Eosinophils levels in the bone marrow after depletion has been added to Figure 7fi.

*Also, since the proposed mechanism relates to monocyte-derived macrophages, why would resident, monocyte-independent LPMs be so strongly affected by eosinophil depletion?*

As discussed in our response to point 3i, our antibody depletion experiments showed an unexpected reduction in LPMs which is likely due to the gut dysbiosis resulting from eosinophil deficiency. LPMs are known to travel to the gut to repair damage caused by dysbiosis (Honda M et al, Nature Communications, 2021). This has been discussed in lines 594-600.

*Minor points:*

*1. Line 86: given the differences between SPM and LPM, it's important to add here that LPM derive from embryonic yolk sac/fetal liver, not monocytes.*

This information has been added.

*2. Line 98-99: are these all resident, non-monocyte derived macrophages?*

The macrophages described in lines 98-99 represent both monocyte derived and resident macrophages in these tissues. The text has been modified to include this information.

*3. The symbol code for PBS and E. coli needs to be inserted into Figure 2C*

These symbol codes have been added in lines 202-3.

*4. Line 278: the call out of 'Figure 4c iv and v' should be Figure 4a iv and v.*

Thank you for spotting this error which has been corrected.

*5. Lines 336-337: One obvious point to address is whether the decrease in Ccr5 might explain decreased levels of SPMs in PC.*

We do not present data on a CCR5 reduction in the peritoneal cavity in the manuscript.

**REVIEWER COMMENTS**

Reviewer #1 (Remarks to the Author):

*Reviewer #1 (Remarks to the Author):*

*In their response, the authors indicate the following:*

*1. that the study was conducted using non-littermates with confirmation in Figure 6 of the main phenotype (reduced peritoneal cavity macrophages, reduced bone marrow eosinophils) using macrophage specific knockout of Ackr2 and littermate controls.*

*2. They agree with the criticism raised that 'The mechanism by which Ccl11 is modestly increased in bone marrow is not established. It is consistent with the scavenging function of Ackr2 among other possibilities (increased transcription, translation, producing cells, all of which might be affected by microbiome differences, etc),...*

*3. that the evidence for 'specific functional Ackr2 expression on' bone marrow macrophages is suggestive, and not definitive.*

*4. that it would be unethical to rederive their conditional Ackr2 kos to ascertain whether they phenocopy the increased Ccl11 protein in bone marrow observed in total kos, since this was not originally requested and is therefore a 'supplemental' request.*

*5. in response to my skepticism about the biological significance of 'a 0.3 pM difference in Ccl11 concentration in wt vs ko serum, the authors respond that 'These concentrations have been expressed as pg/mg of tissue and as such the molarity cannot be measured.*

*6. in response to the minor point raised 'One obvious point to address is whether the decrease in Ccr5 might explain decreased levels of SPMs in PC.' The authors respond 'We do not present data on a CCR5 reduction in the peritoneal cavity in the manuscript.'*

*Overall, the authors have provided adequate caveats in the Results section to address the limitations in the data that limit the Conclusions that can be drawn. However, they have not done this in the Title, Abstract or Discussion, which still overstate the findings for the following reasons:*

*1. Regarding point 1, the use of non-cohoused, non-littermate wt controls in studies of genetically modified mice is notorious for the introduction of potential artefacts due to genetic drift between the wt and ko strains. This has been substantially mitigated by use of macrophage-specific Ackr2 ko mice, and RNA and some functional evidence that Ackr2 is functionally expressed in macrophages.*

*2. The use of the word egress to describe the effect of ACKR2 deficiency on absolute eosinophil levels in the bone marrow is not supported by the data, which just present levels at a single timepoint. Experiments to justify attributing the changes to dynamic effects on egress would include effects of Ackr2 blocking agents on mobilization and ideally direct imaging of eosinophil movement. There are multiple other possible mechanisms for why eosinophil levels may be lower in ko mice that were not addressed. Thus, to attribute the differences to an egress mechanism is not justified by the Ccl11 data, including the Ccl11 challenge data which are not really relevant to the conditions these mice find themselves in (low levels of Ccl11 in bone marrow and blood, small differences in those levels between ko and wt).*

We agree with the reviewer and have removed the word egress from the title and throughout the manuscript.

*3. The transcriptomic data do not address whether the differences found between wt and ko macrophages are due directly to eosinophils. The data in Figure 7 only address a couple of macrophage proteins and show very small effects in vitro when macs are exposed to eosoinophils.*

We agree with the reviewer and acknowledge that our transcriptomic data alone does not definitively show that eosinophils are responsible for the differences between WT and ACKR2-/- macrophages. Rather, what we have shown is that co-culture of eosinophil and monocytes led to altered expression of a limited number of key genes by monocytes, in keeping with our hypothesis. We have added discussion of the limitations of the transcriptomic data, and added a statement that the biological significance of the small effects observed are unclear in lines in 585-590.

In Figure 7 bone marrow derived macrophages (BMDMs) were not exposed to eosinophils. In Figure 7d eosinophils were added to total bone marrow cells on day 0 of culture, which after differentiation to BMDMs on day 5 were incubated for 1 h with *E.coli* particles. We apologise for the incomplete legend for Figure 7 d. It has now been amended in line 506-508. The addition of eosinophils on day 0 significantly increases the breakdown of *E.coli* particles by BMDMs from ACKR2-/- animals (Figure 7d).

*4. Regarding point 2 above, despite the agreement the authors still attribute the changes they measured for Ccl11 in bone marrow to scavenging in the abstract and discussion. Without measurement of extracellular Ccl11 levels in bone marrow from wt and straight ko, without any measurement of Ccl11 in flox and LysM cre mouse bone marrow, and given the very small changes observed in the straight ko vs wt for Ccl11 levels in bone marrow and for Ccl22 uptake by macrophages, attributing the decreased eosinophil content of the ko bone marrow to the loss of Ackr2 scavenging of Ccl11 is unjustified.*

We apologise for not making these changes in the last revision. We have removed the references to scavenging of CCL11 throughout the manuscript and toned down our description of the CCL11 data in lines 382-386 and 423-426.

*5. Regarding point 5 above, the authors have not answered the question, which was about the minor difference in molarity of Ccl11 that they reported for wt vs straight ko in serum. I indicated a 0.3 pM difference but it's actually more like 20 pM. The authors apparently misread this because there response addresses measurements of tissue in pg/mg. The biological significance of such a small difference in serum is uncertain.*

We apologise for our misunderstanding and have removed the references to scavenging of CCL11 throughout the manuscript and toned down our description of the data in lines 382-386.

*6. Regarding point 6 above, I incorrectly mistakenly wrote Ccr5 when the relevant molecule in the paper was Ccl5.*

We agree that the altered transcription of many genes including *CCL5* by monocytes from ACKR2-/- animals likely contribute to the reduced monocyte to macrophage differentiation and have added this in lines 606-608.

*As mentioned in previous reviews of this paper, there is a high density of interesting and novel findings. That remains true; however, the mechanistic conclusions in this revision remain overstated.*
